# Inter- and intra-island speciation and their morphological and ecological correlates in *Aeonium* (Crassulaceae), a species-rich Macaronesian radiation

**DOI:** 10.1093/aob/mcad033

**Published:** 2023-02-23

**Authors:** Thibaud F E Messerschmid, Stefan Abrahamczyk, Ángel Bañares-Baudet, Miguel A Brilhante, Urs Eggli, Philipp Hühn, Joachim W Kadereit, Patrícia dos Santos, Jurriaan M de Vos, Gudrun Kadereit

**Affiliations:** Botanischer Garten München-Nymphenburg, Staatliche Naturwissenschaftliche Sammlungen Bayerns, 80638 München, Germany; Prinzessin Therese von Bayern-Lehrstuhl für Systematik, Biodiversität & Evolution der Pflanzen, Ludwig-Maximilians-Universität München, 80638 München, Germany; Nees-Institut für Biodiversität der Pflanzen, Rheinische Friedrich-Wilhelms-Universität Bonn, 53115 Bonn, Germany; Abteilung Botanik, Staatliches Museum für Naturkunde Stuttgart, 70191 Stuttgart, Germany; Departamento de Botánica, Ecología y Fisiología Vegetal, Universidad de La Laguna, E-38200 La Laguna, Tenerife, Spain; Linking Landscape, Environment, Agriculture and Food (LEAF), Instituto Superior de Agronomia (ISA), Universidade de Lisboa, 1340-017 Lisboa, Portugal; Sukkulenten-Sammlung Zürich/Grün Stadt Zürich, 8002 Zürich, Switzerland; Institut für Organismische und Molekulare Evolutionsbiologie, Johannes Gutenberg-Universität Mainz, 55099 Mainz, Germany; Institut für Organismische und Molekulare Evolutionsbiologie, Johannes Gutenberg-Universität Mainz, 55099 Mainz, Germany; Centre for Ecology, Evolution and Environmental Changes (cE3c) and Global Change and Sustainability Institute (CHANGE), Faculdade de Ciências, Universidade de Lisboa, 1749-016 Lisboa, Portugal; Department of Environmental Sciences – Botany, University of Basel, 4056 Basel, Switzerland; Department of Environmental Sciences – Botany, University of Basel, 4056 Basel, Switzerland; Botanischer Garten München-Nymphenburg, Staatliche Naturwissenschaftliche Sammlungen Bayerns, 80638 München, Germany; Prinzessin Therese von Bayern-Lehrstuhl für Systematik, Biodiversität & Evolution der Pflanzen, Ludwig-Maximilians-Universität München, 80638 München, Germany

**Keywords:** *Aeonium*, ancestral area reconstruction, biogeographical stochastic mapping, Canary Islands, diversification, island biogeography, molecular dating

## Abstract

**Background and Aims:**

The most species-rich and ecologically diverse plant radiation on the Canary Islands is the *Aeonium* alliance (Crassulaceae). In island radiations like this, speciation can take place either within islands or following dispersal between islands. Aiming at quantifying intra- and inter-island speciation events in the evolution of *Aeonium*, and exploring their consequences, we hypothesized that (1) intra-island diversification resulted in stronger ecological divergence of sister lineages, and that (2) taxa on islands with a longer history of habitation by *Aeonium* show stronger ecological differentiation and produce fewer natural hybrids.

**Methods:**

We studied the biogeographical and ecological setting of diversification processes in *Aeonium* with a fully sampled and dated phylogeny inferred using a ddRADseq approach. Ancestral areas and biogeographical events were reconstructed in BioGeoBEARS. Eleven morphological characters and three habitat characteristics were taken into account to quantify the morphological and ecological divergence between sister lineages. A co-occurrence matrix of all *Aeonium* taxa is presented to assess the spatial separation of taxa on each island.

**Key Results:**

We found intra- and inter-island diversification events in almost equal numbers. In lineages that diversified within single islands, morphological and ecological divergence was more pronounced than in lineages derived from inter-island diversification, but only the difference in morphological divergence was significant. Those islands with the longest history of habitation by *Aeonium* had the lowest percentages of co-occurring and hybridizing taxon pairs compared with islands where *Aeonium* arrived later.

**Conclusions:**

Our findings illustrate the importance of both inter- and intra-island speciation, the latter of which is potentially sympatric speciation. Speciation on the same island entailed significantly higher levels of morphological divergence compared with inter-island speciation, but ecological divergence was not significantly different. Longer periods of shared island habitation resulted in the evolution of a higher degree of spatial separation and stronger reproductive barriers.

## INTRODUCTION

Oceanic islands and archipelagos have long been considered natural laboratories where evolutionary and biogeographical processes that are complex on a continental scale are simplified and easier to study (Whittaker and Fernández-Palacios, 2007). Patterns of biodiversity on islands have fostered ideas and theories about island biogeography (Carlquist, 1965, 1974; MacArthur and Wilson, 1967) and the theory of evolution itself (Darwin, 1859). Due to their geographical isolation and limited size, islands are characterized by a high degree of endemism but relatively low overall species numbers in comparison with continents (Whittaker and Fernández-Palacios, 2007). The floras and faunas of oceanic archipelagos are rich in monophyletic lineages that diversified into distinct species often endemic to one or few islands. These lineages are commonly referred to as island radiations (e.g. Silvertown *et al.*, 2005). Prominent examples of such island radiations are the Caribbean *Anolis* lizards (Losos *et al.*, 1998), Hawaiian honeycreepers (Lerner *et al.*, 2011), lobeliads (Givnish *et al.*, 2009) and silverswords (Baldwin, 2003), and the *Aeonium* alliance (Liu, 1989; Mort *et al.*, 2002) of the Macaronesian floristic region. All of the afore-cited publications have discussed the respective lineages as examples of adaptive radiation (Givnish, 1997; Sanderson, 1998; Gillespie *et al.*, 2020), and therefore as showcases of ecological and morphological diversification. The biogeographical correlates of speciation events in island radiations, i.e. within single islands (intra-island) or following dispersal between islands (inter-island), can be identified through ancestral area reconstruction (AAR) in combination with biogeographical stochastic mapping (BSM) (Phillips *et al.*, 2020; White *et al.*, 2020). Of these two biogeographical correlates of speciation, only intra-island speciation may or may not represent sympatric speciation and therefore potentially entails stronger ecological divergence of sister lineages (Gillespie *et al.*, 2020) than in the case of inter-island speciation, which is always allopatric.

**Fig. 1.**
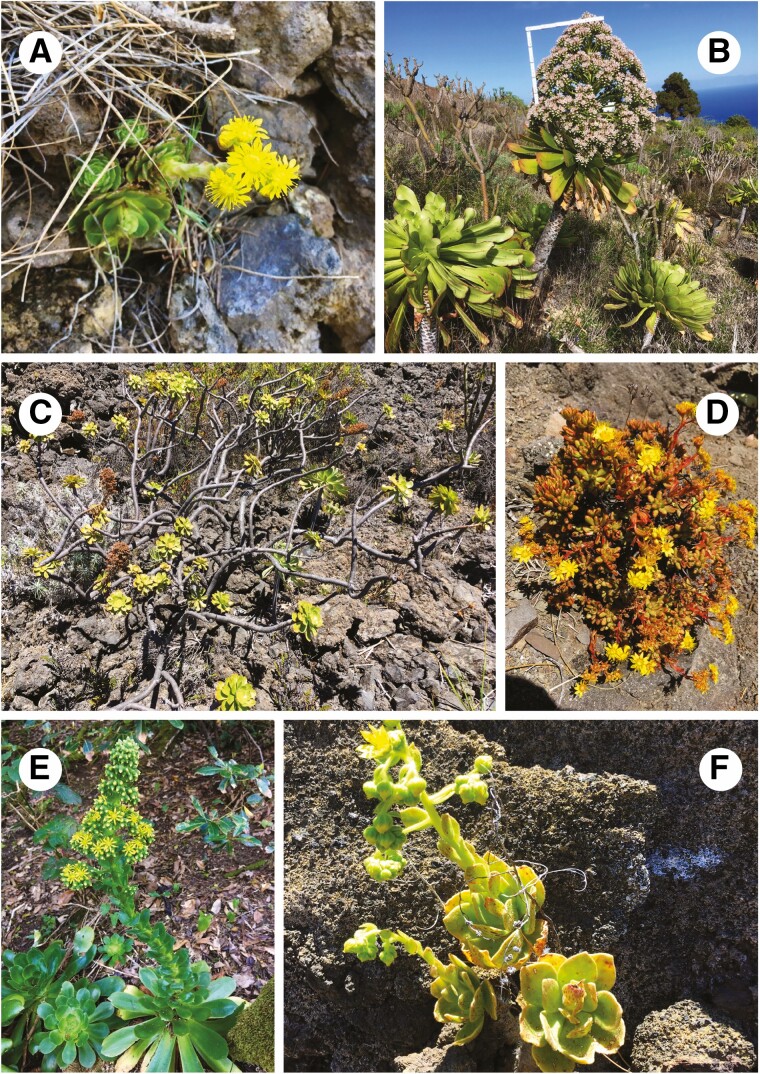
(A) Section *Greenovia*: *A. aizoon*, a small (up to 15 cm high) rosette plant endemic to Tenerife, with ±20-merous nectary-lacking flowers. (B) Section *Leuconium*: *A. urbicum* subsp. *meridionale*, a tall (1.5–2 m high) monocarpic plant with unbranched woody stem and hundreds of white-reddish flowers. Both subspecies of *A. urbicum* are endemic to Tenerife. (C) Section *Aeonium*: *A. arboreum* subsp. *holochrysum* var. *holochrysum*, a laxly branched, spacious and tall (up to 1.5 m high) shrub with yellow flowers appearing in winter, distributed across El Hierro, La Palma and Tenerife. (D) Section *Petrothamnium*: *A. sedifolium*, a small (up to 25 cm high), densely branched shrublet with little thick and clavate leaves, native to La Palma, La Gomera and Tenerife. (E) Section *Canariensia*: *A. cuneatum*, a sturdy, herbaceous rosette plant with distinctly glaucous leaves endemic to the laurel forest of northern Tenerife (Teno and Anaga). (F) Section *Chrysocome*: *A. smithii*, a shrublet with densely hairy branches and leaves featuring conspicuous reddish tannic stripes.

*Aeonium* (including *Greenovia*, Crassulaceae; Fig. 1) is an iconic group of succulent plants that enjoys high popularity in horticulture and botanical research (e.g. Praeger, 1932; Liu, 1989; Jorgensen and Olesen, 2001; Mort *et al.*, 2002). There are currently 40 accepted species of *Aeonium* (Mes, 1995; Nyffeler, 2003; Bañares Baudet, 2015*a*) with a centre of diversity in the Canary Islands (Table 1) which, together with the Azores, Madeiran archipelago, Salvage Islands and Cape Verde Islands, form a floristic region traditionally known as Macaronesia (e.g. Hansen and Sunding, 1993). They are perennial herbs or subshrubs, some of them unbranched and then monocarpic with a single rosette. *Aeonium* belongs to the most species-rich radiation of the Macaronesian flora (Jorgensen and Olesen, 2001), the *Aeonium* alliance (tribe Aeonieae of Crassulaceae subfamily Sempervivoideae), which also includes *Aichryson* (15 species) and *Monanthes* (11 species; Praeger, 1932; Mort *et al.*, 2002; Bañares Baudet, 2015*a*). The closest relatives of the *Aeonium* alliance are a grade of eight northwest African species of *Sedum* (Mes, 1995; Dürig, 2021), suggesting colonization of the Canary Islands from northwest Africa or the western Mediterranean region. The *Aeonium* alliance shows much diversity in growth form (Lems, 1960; Liu, 1989; Mes and ’t Hart, 1996), ecological niche and photosynthetic pathway (Tenhunen *et al.*, 1982; Lösch, 1990; Pilon-Smits *et al.*, 1992; Mort *et al.*, 2007), and has therefore been among the most important systems for the study of island plant radiations (Givnish, 2010; Thiv *et al.*, 2010*a*) and the evolution of insular woodiness (Mes *et al.*, 1996; van Huysduynen *et al.*, 2021). Within the *Aeonium* alliance, *Aichryson* and *Monanthes* are strictly endemic to the Macaronesian Islands (Bañares Baudet, 2015*a*). However, three species of *Aeonium* are found on the African continent (*A. korneliuslemsii*, *A. stuessyi* and *A. leucoblepharum*, the latter also in the Yemen). In addition to the 34 Canary Island species of *Aeonium*, two species are found on Madeira (i.e. *A. glandulosum* and *A. glutinosum*) and one on the Cape Verde Islands (*A. gorgoneum*; Table 1). Twenty-seven of the 37 insular species of *Aeonium* are single-island endemics (SIEs) while the remaining 10 species are multi-island endemics (MIEs). Four of these ten MIE species contain intraspecific taxa that occupy different islands. Similar to other lineages of Crassulaceae, e.g. *Echeveria* (Uhl, 1992) or *Kalanchoe* (Smith *et al.*, 2022), many species of *Aeonium* are interfertile and produce a number of naturally occurring hybrids (Bañares Baudet, 2015*a*; Arango Toro, 2017, 2019*a*, 2019*b*). However, little is known about the rate at which reproductive barriers evolve in distinct species of these genera and in plants in general (Baack *et al.*, 2015).

**Table 1.** Distribution, sectional affiliation and ploidy level of all currently accepted *Aeonium* species following Bañares Baudet (2015*a* and updated since) for Canary Island endemics (except *A. escobarii* and *A. hierrense*: Rebmann, 2013) and Mes (1995) for taxa distributed outside the Canary Islands. C, Gran Canaria; EA, East Africa (including the southwestern part of the Arabian Peninsula); F, Fuerteventura; G, La Gomera; H, El Hierro; L, Lanzarote; Mc, Morocco; Md, Madeira; P, La Palma; T, Tenerife; V, Cape Verde

Section	Species	Subspecies/variety	H	P	G	T	C	F	L	Mc	EA	Md	V	Ploidy
*Aeonium*	*A. arboreum* Webb & Berthel.	subsp. *arboreum*					X							2*x*^a^, 4*x*^b,c^
		subsp. *holochrysum* (H.Y.Liu) Bañares^d^	X	X	X	X								2*x*^a,b,e,f^, 4*x*^c^
	*A. balsamiferum* Webb & Berthel.							X	X					4*x*^a,b,c^
	*A. gorgoneum* J.A.Schmidt												X	2*x*^a^, 4*x*^f^
	*A. korneliuslemsii* H.Y.Liu									X				4*x*^a^
	*A. leucoblepharum* Webb ex A.Rich.										X			2*x*^a^, 4*x*^c^
	*A. simsii* (Sweet) Stearn						X							2*x*^b^, 3*x*^a,b^, 4*x*^b,c,f^
	*A. stuessyi* H.Y.Liu										X			Unknown
	*A. undulatum* Webb & Berthel.						X							4*x*^a,b,c^
*Canariensia*	*A. canariense* (L.) Webb & Berthel.	subsp. *canariense*				X								2*x*^a,b,e,f^
		subsp. *christii* (Burchard) Bañares	X	X										2*x*^a,b,e,f^
		subsp. *latifolium* (Burchard) Bañares			X									2*x*^a,b,f^
		subsp. *virgineum* (Webb) Bañares					X							2*x*^a,b,f^
	*A. cuneatum* Webb & Berthel.					X								2*x*^a,b,f^
	*A. tabuliforme* Webb & Berthel.					X								2*x*^a,b,e,f^
*Chrysocome*	*A. smithii* Webb & Berthel.					X								2*x*^a,b,e,f^
	*A. spathulatum* (Hornem.) Praeger		X	X	X	X	X							2*x*^a,b,e,f^
*Goochiae*	*A. goochiae* Webb & Berthel.			X										2*x*^a,b,e^
	*A. lindleyi* Webb & Berthel.	subsp. *lindleyi*				X								2*x*^a,b,e,f^
		subsp. *viscatum* (Bolle) Bañares			X									2*x*^a,b,f^
*Greenovia*	*A. aizoon* (Bolle) T.Mes					X								2*x*^f^
	*A. aureum* (C.Sm. ex Hornem.) T.Mes					X	X							2*x*^b,f^
	*A. diplocyclum* (Webb ex Bolle) T.Mes		X	X	X									2*x*^b,f^, 4*x*^b,c^
	*A. dodrantale* (Willd.) T.Mes					X								2*x*^b,f^
*Leuconium*	*A. appendiculatum* Bañares				X									2*x*^f^
	*A. castello-paivae* Bolle				X									2*x*^a,b,f^
	*A. ciliatum* Webb & Berthel.					X								2*x*^a,b,e,f^
	*A. davidbramwellii* H.Y.Liu			X										2*x*^a,f^
	*A. decorum* Webb ex Bolle	var. *alucense* Bañares & M.Marrero			X									Unknown
		var. *decorum*			X	X								2*x*^a,b,f^
	*A. escobarii* Rebmann & Malkm.-Huss.			X										Unknown
	*A. gomerense* (Praeger) Praeger				X									2*x*^a,b,f^
	*A. haworthii* Webb & Berthel.					X								4*x*^a,b,c,f^
	*A. hierrense* (Murray) Pit. & Proust		X											2*x*^a^
	*A. lancerottense* (Praeger) Praeger								X					2*x*^a,b^
	*A. mascaense* Bramwell^g^					X								Unknown
	*A. nobile* (Praeger) Praeger			X										2*x*^a,b,f^
	*A. percarneum* (Murray) Pit. & Proust						X							2*x*^a,b,f^
	*A. pseudurbicum* Bañares					X								2*x*^f^
	*A. urbicum* (C.Sm. ex Hornem.) Webb & Berthel.	subsp. *meridionale* (Bañares) Bañares				X								2*x*^f^
		subsp. *urbicum*				X								2*x*^a,b,e^, 4*x*^c^
	*A. valverdense* (Praeger) Praeger		X											2*x*^a^, 4*x*^c^
	*A. volkeri* E.Hern. & Bañares					X								4*x*^f^
*Patinaria*	*A. glandulosum* (Aiton) Webb & Berthel.											X		2*x*^a,f^
*Petrothamnium*	*A. saundersii* Bolle				X									2*x*^a,f^
	*A. sedifolium* (Webb ex Bolle) Pit. & Proust			X	X	X								2*x*^a,b,e,f^
*Pittonium*	*A. glutinosum* (Aiton) Webb & Berthel.											X		2*x*^a,b,f^

^a^ Liu (1989).

^b^ Uhl (1961).

^c^ Mes (1995).

^d^ *Aeonium arboreum* subsp. *holochrysum* is here considered geographically separated, with *A. arboreum* subsp. *holochrysum* var. *holochrysum* restricted to El Hierro, La Palma and Tenerife, and *A. arboreum* subsp. *holochrysum* var. *rubrolineatum* restricted to La Gomera. Although the former variety has been reported from La Gomera (e.g. Voggenreiter, 1999), the taxonomy of *A. arboreum* subsp. *holochrysum* on La Gomera is in need of revision and we here follow Bañares Baudet (2015*a*).

^e^ Suda *et al.* (2005).

^f^ Brilhante *et al.* (2021).

^g^ *Aeonium mascaense* is extinct in the wild and was only known from the Barranco de Masca (Tenerife). However, due to its very recent extinction in the 20th century, we consider this species present on Tenerife for the sake of accuracy of the biogeographical analysis.

In order to answer fundamental questions concerning biogeographical correlates of diversification events and ecological divergence following such diversification events, a fully resolved and dated phylogeny, AAR and analysis of detailed distributional, morphological and ecological data are needed. So far, molecular phylogenetic studies of *Aeonium* have been unsuccessful in resolving the phylogeny of the genus, mainly because data from restriction fragment length polymorphism (RFLP) analyses (Mes and ’t Hart, 1996) or sequences of only a few molecular markers (Mort *et al.*, 2002) are not sufficiently informative. Thanks to advances in next-generation sequencing methods, recent years have seen improvements in our phylogenetic and evolutionary knowledge of important Macaronesian plant radiations, e.g. *Tolpis* (Asteraceae; Mort *et al.*, 2015), *Micromeria* (Lamiaceae; Curto *et al.*, 2018), *Argyranthemum* (Asteraceae; White *et al.*, 2020) and *Helianthemum* (Cistaceae; Albaladejo *et al.*, 2021). These studies used high-throughput sequencing methods for reduced-representation libraries of genomic DNA, such as genotyping by sequencing (GBS; Elshire *et al.*, 2011) or restriction-site-associated DNA sequencing (RADseq; Baird *et al.*, 2008; Andrews *et al.*, 2016). However, due to the usually short sequence reads obtained with these methods, most of these studies based their phylogenetic analyses on datasets of concatenated DNA loci, which precludes consideration of independently evolving sequence markers potentially containing incongruent phylogenetic signal (Kubatko and Degnan, 2007; Pirie, 2015).

We here present a largely resolved and dated phylogeny of *Aeonium* sampling all currently accepted species and using a modified ddRADseq (double digest restriction-site associated DNA sequencing) library preparation protocol, with which long loci can be generated (Hühn *et al.*, 2022) and which allows us to deploy coalescent-based summary methods. Using this phylogeny in combination with detailed distributional, morphological and ecological data, the main aims of our biogeographical analysis are to identify migration between islands and out of the Canary Islands, and to quantify the relative contributions of inter- and intra-island speciation to the diversification in *Aeonium*. We hypothesize that (1) clades that diversified within single islands have evolved higher morphological and ecological diversity than clades that diversified by repeated dispersal between islands. Furthermore, based on the assumption that reproductive isolation between distinct species evolves with time, we hypothesize that (2) different *Aeonium* taxa show stronger ecological differentiation and produce fewer natural hybrids on islands that have been inhabited for a long time when compared with islands that were colonized by *Aeonium* more recently.

## MATERIALS AND METHODS

### Taxon sampling

All 40 species and all intraspecific taxa of *Aeonium* except for *A. decorum* var. *alucense* were sampled for DNA analysis. Of these, five species and one subspecies had never been included in a molecular phylogeny before. *Monanthes brachycaulos* and *M. muralis* were chosen as outgroup because *Monanthes* (excl. *M. icterica*) is sister to *Aeonium* (Mes, 1995; Mort *et al.*, 2002). Overall, 49 accessions of *Aeonium* and two accessions of *Monanthes* were sampled (Supplementary Data Table S1). With the exception of *A. leucoblepharum* and *A. sedifolium*, for which two different accessions were sampled, each taxon was represented by one sample. The majority of accessions were collected and rapidly silica-dried during field work between March and May 2019. The remaining accessions were taken from mostly well-documented collections of living plants or from further field work (see Acknowledgements section).

### Compilation of morphological, ecological and species occurrence data

Morphological characters commonly used for the identification of *Aeonium* species (i.e. growth form, indumentum of branches, leaves and inflorescences, scaliness of leaf scars, presence of cilia and tannic stripes on leaves, flower merism, petal colour and presence of carpel appendages and hypogynous nectar glands) were obtained from Liu (1989), Nyffeler (2003), Rebmann (2013) and Bañares Baudet (2015*a*). We followed Bañares Baudet’s (2015*a*) classification of growth forms and distinguished between acaulescent rosette plants, nanophanerophytes (branched or unbranched) and chamaephytes, with chamaephytes having vegetative buds <25 cm above ground. Climate zones of the Canary Islands were characterized in terms of the predominant thermotype and ombrotype (Rivas-Martínez *et al.*, 2017) of each area. Thermo- and ombrotype are ordinal categories defined by threshold values of the thermicity index (*I*_t_) and ombrothermic index (*I*_o_), respectively (Tables 1 and 2 in Rivas-Martínez *et al.*, 1997). The thermotypes relevant for our study range from infra-Mediterranean (*I*_t_ > 450) to supra-Mediterranean (210 ≥ *I*_t_ > 80) and ombrotypes from arid (0.9 ≥ *I*_o_ > 0.5) to hyperhumid (*I*_o_ > 12; Rivas-Martínez *et al.*, 1997). Thermo- and ombrotypes in the distribution range of each taxon (i.e. species, subspecies or variety) were taken from Bañares Baudet (2015*a*) for the Canary Island taxa, from Capelo *et al.* (2005) for species from Madeira, and from Rivas-Martínez *et al.* (2011) for *A. korneliuslemsii* from Morocco, without weighting the proportion of different thermo- and ombrotypes across the taxa’s distributional ranges. Information about the elevational range of taxa was compiled from Bañares Baudet (2015*a*) when available, and complemented with data by Voggenreiter (1974) for *A. ciliatum*, by Bramwell (1982) for *A. mascaense*, by Liu (1989) for *A. castello-paivae*, species of sect. *Chrysocome*, as well as for all species from outside the Canary Islands, by Bañares Baudet (1999) for *A. urbicum* subsp. *meridionale*, and by Nyffeler (2003) for *A. diplocyclum*. Using the Biodiversity Data Bank of the Canary Islands (www.biodiversidadcanarias.es) and, for the extra-Canarian species, the distribution maps provided by Liu (1989), we compiled a co-occurrence matrix for all taxa of *Aeonium* and noted the occurrence of published natural hybrids between each pair of co-occurring taxa (Praeger, 1929; Bañares Baudet, 2015*a*, 2015*b*; Mottram, 2015; Arango Toro, 2017, 2019*a*, 2019*b*). Co-occurrence between taxa was not quantified and was accepted even when the overlap of distribution areas was small. Rates of co-occurrence and hybridization between taxa were calculated individually for each of the Canary Islands, relative to the number of possible combinations of any two taxa occurring on each island.

### DNA extraction, library preparation and sequencing

DNA was extracted either from silica-dried material when this yielded DNA of sufficiently high quality and integrity, or from flash-frozen leaves of living plants. For the dried material, the DNeasy Plant Mini Kit (Qiagen, Hilden, Germany) was used following the manufacturer’s protocol but with an extended lysis time between 1 and 2 h using 50 % more of the lysis and binding buffers than recommended in the protocol. Flash-frozen whole leaves or leaf epidermal shavings (Supplementary Data Table S1) were ground manually in liquid nitrogen with small amounts of sea sand (Carl Roth, Karlsruhe, Germany). DNA was then isolated using the NucleoSpin Plant II kit (Macherey-Nagel, Düren, Germany) according to the manufacturer’s protocol for CTAB-based extraction. Here, the volumes of the lysis and binding buffers were doubled, and lysis time was extended to 1 h. For both extraction protocols, an extended time of 1 h was allowed for the elution of DNA from the column membrane to increase the yield. DNA quality was checked on 0.8 % agarose gels and DNA concentrations were quantified using a Qubit 3.0 fluorometer (Thermo Fisher Scientific, Waltham, MA, USA).

The ddRADseq libraries were prepared according to Hühn *et al.* (2022) with the following modifications. In order to include samples with a DNA concentration <40 ng µL^−1^, we performed several digestion reactions with 5 µL of DNA isolate per reaction, so that a total amount of 200 ng genomic DNA per sample could be used. For adapter ligation following digestion, 8 µL of each adapter solution was used per 200 ng of sample DNA in a total reaction volume of 50 µL. The optimal amount of adapters had been determined before by adapter titration. After multiplexing, purification and size selection of the pools, ten low-cycle PCRs per pool were carried out separately and were subsequently re-pooled. After final purification, the libraries were sequenced on an Illumina MiSeq (San Diego, CA, USA; Reagent Kit v3 600-cycle) with 300-bp paired-end reads, either at StarSEQ (Mainz, Germany) or at Macrogen (Seoul, Republic of Korea).

### Data treatment and sequence assembly

Sequence data quality was assessed using FastQC v0.11.4 (http://www.bioinformatics.babraham.ac.uk/projects/fastqc/). The sequencing quality of the reverse (R2) reads was consistently lower than that of the forward (R1) reads (Supplementary Data Files S1 and S2). The library was demultiplexed using ipyrad v0.9.52 (Eaton and Overcast, 2020), once for each restriction site of the two restriction enzymes used. Demultiplexed sequences were deposited in the NCBI Sequence Read Archive (www.ncbi.nlm.nih.gov/sra; BioProject ID PRJNA848764). Adapter sequences were removed using cutadapt v2.3 (Martin, 2011). Sequence data from diploid and potentially tetraploid samples (see Table 1 for information on the ploidy levels of all *Aeonium* taxa) were analysed separately throughout the first assembly steps, i.e. (1) clustering of reads within samples based on sequence similarity into putative loci (in-sample clustering, ISC) and (2) consensus calling of alleles. Information about chromosome numbers, genome size and inferred ploidy levels of the species sampled was taken from the literature (Uhl, 1961; Liu, 1989; Mes, 1995; Suda *et al.*, 2005; Brilhante *et al.*, 2021; Table 1). Samples from species without information on ploidy level were treated as tetraploid because this minimized the risk of merging different alleles into one. In order to find the most suitable clustering thresholds (CTs) for data assembly, we used an empirical approach based on the evaluation of several metrics of the ipyrad pipeline. First, with the ipyrad assembly steps 1–5 ISC was carried out, and the resulting number of clusters, read depth, number of flagged paralogues and heterozygosity were visualized in the form of box- and scatterplots as a function of the tested CT range (0.81–0.99) using SPSS Statistics v23 (IBM Corp., 2015). To detect signals of over- and undermerging of reads into clusters using these plots, the most appropriate CT value for ISC was chosen as described by Hühn *et al.* (2022), independently for diploid and tetraploid samples. The resulting assemblies of clusters within samples were then merged and in turn tested for the CT value most suitable for between-sample clustering (BSC, steps 6 and 7 of the *ipyrad* pipeline; values from 0.81 to 0.99 tested). In order to identify the CT above which loci are subject to undermerging (Paris *et al.*, 2017), the metrics corresponding to the resulting BSC runs were assessed with respect to the number of new polymorphic loci that were gained when the CT was increased by 0.01. Performing a BLASTN (ncbi-blast-2.2.28+; Camacho *et al.*, 2009) search of all loci against four Crassulaceae reference plastomes [see Hühn *et al.* (2022) for GenBank accession numbers], loci of the plastid genome were removed from the final assembly. Additionally, all loci containing zero parsimony-informative sites (PISs) were removed from the assembly prior to phylogenetic analyses.

### Phylogenetic analyses

We analysed the final assembly in four different ways using maximum likelihood (ML) implemented in RAxML-NG v0.9.0 (Kozlov *et al.*, 2019). (1) The full assembly of all loci was concatenated into a supermatrix using FASconCAT v1.11 (Kück and Meusemann, 2010), from which the phylogeny was inferred (all/RAxML). (2) Individual gene trees of all loci of the same dataset were inferred using RAxML-NG and were subsequently used for species tree inference in ASTRAL-III v5.7.4 (Zhang *et al.*, 2018; all/ASTRAL). Based on the uneven distribution of locus lengths and sample coverage throughout the locus length range (Supplementary Data File S3), we filtered loci from the assembly so that only loci with lengths between 320 and 500 nucleotides (nt) were retained, in the following referred to as the 320–500 dataset. The loci removed probably were the product of incorrect assembly resulting from the consistently lower quality of reverse reads compared with forward reads (Supplementary Data Files S1 and S2), leading to biased locus and sample coverage. (3) The resulting reduced dataset was in turn again concatenated into a supermatrix using FASconCAT for phylogenetic inference in RAxML (320–500/RAxML), and (4) individual gene trees were inferred for the same reduced dataset and used for species tree inference in ASTRAL-III (320–500/ASTRAL).

For each RAxML run, the GTR+Γ substitution model was used and 1000 bootstrap replicates were run. All ASTRAL runs were performed with default settings, and multilocus bootstrapping (Seo, 2008) was used in order to obtain branch support values for the resulting species trees.

### Molecular dating

We only used secondary age estimates for calibration of divergence time because no fossils with affinity to the Crassulaceae are known (Messerschmid *et al.*, 2020). We carried out two subsequent dating analyses. (1) A Crassulaceae-wide partitioned alignment of ITS sequences (Messerschmid *et al.*, 2020) was expanded by 11 additional accessions of *Aichryson*, *Monanthes* and *Aeonium* (Supplementary Data Table S1) in order to better represent the Macaronesian *Aeonium* alliance. (2) Using the age estimate for the split of *Aeonium* and *Monanthes* from this first dating analysis [i.e. 11.62 million years (myr); 95 % highest posterior density (HPD) = 6.56–17.1 myr] as the calibration point, divergence time estimation was carried out for our *Aeonium* phylogeny inferred from ddRADseq data.

The family-wide dating analysis (1) was performed using BEAST v2.4.8 (Bouckaert *et al.*, 2014) implemented in the CIPRES Science Gateway (Miller *et al.*, 2010). The most recently published estimates (Ramírez-Barahona *et al.*, 2020) of the stem (116.25 myr; 95 % HPD = 92.58–148.07 myr) and crown age (72.57 myr; 95 % HPD = 49.96–96.16 myr) of Crassulaceae were used as calibration points. Settings for the BEAST analyses were the same as described by Messerschmid *et al.* (2020). Four independent runs were performed and the resulting log files were checked for convergence using Tracer v1.5 (Rambaut and Drummond, 2007). Runs were combined using LogCombiner v2.4.5 (Bouckaert *et al.*, 2014) predefining a burn-in of 10 %, and a maximum clade credibility tree was constructed using TreeAnnotator v1.8.3 (Rambaut and Drummond, 2016) and inspected using FigTree v1.3.1 (Rambaut, 2009).

We used BEAST v2.6.4 (Bouckaert *et al.*, 2019) for the dating analysis of the *Aeonium* phylogeny (2). We predefined all those clades as monophyletic that had high bootstrap support (BS > 95) in all four phylogenetic analyses of *Aeonium* (see Phylogenetic analyses section above). Initial dating analyses using all loci of the 320–500 dataset resulted in unreasonably recent divergence times (e.g. 3.9–3.92 myr for the split between *Aeonium* and *Monanthes*, which was far off the range of the prior). A similar case of clade age underestimation had recently been reported for *Carex* sect. *Schoenoxiphium* and ascribed to high proportions of missing data (Villaverde *et al.*, 2021). Following these findings, we performed dating analyses with a dataset that contained sequence data for at least 20 taxa at every locus (called dataset min20tax in the following), corresponding to 42 % missingness across the dataset. Also, a log normal instead of a normal prior for the divergence time between *Aeonium* and *Monanthes* was used because of the potentially greater error associated with normal distribution for secondary calibration points (Schenk, 2016). The topologies inferred from the 320–500/RAxML as well as the 320–500/ASTRAL analyses were simultaneously used as starting trees. As for the family-wide dating analysis (see above), four independent runs were carried out with each of the two starting trees, and the four respective runs were checked for convergence and combined as described above.

### Biogeographical reconstruction of ancestral distribution

The dated maximum clade credibility (MCC) tree was used for an AAR analysis using the R package BioGeoBEARS (v.1.1.2; Matzke, 2014, 2018*a*), additionally requiring rexpokit (v0.26.6.7; Matzke *et al.*, 2020) and cladoRcpp (v0.15.1; Matzke, 2018*b*). The seven main islands of the Canarian archipelago as well as the Madeira Islands, the Cape Verde Islands, Morocco and East Africa (including the southwestern part of the Arabian Peninsula) were treated as separate geographical regions in the AAR analysis, resulting in 11 distinct regions. The analysis was time-stratified using four distinct time strata defined by the emergence of Madeira (5 myr ago; Geldmacher *et al.*, 2000), La Palma and El Hierro (1.7 and 1.1 myr ago, respectively; van den Bogaard, 2013). Extant and ancestral taxa were allowed to occupy a maximum number of five geographical regions because *A. spathulatum*, the taxon with the highest number of occupied regions, occurs on five of the Canary Islands. Subsequent to the completion of the AAR analysis, BSM (Matzke, 2016; Dupin *et al.*, 2016) as implemented in BioGeoBEARS was carried out on the model that received the highest log-likelihood (LnL) and corrected Akaike information criterion model weight (AICc_wt) scores (i.e. BAYAREALIKE+J, see Results section) in order to simulate and quantify stochastically mapped cladogenetic and anagenetic events in the evolution of *Aeonium*. Fifty individual biogeographical stochastic maps (BSMs) were simulated (default settings).

### Calculation of morphological and ecological divergence between sister lineages

**Fig. 2.**
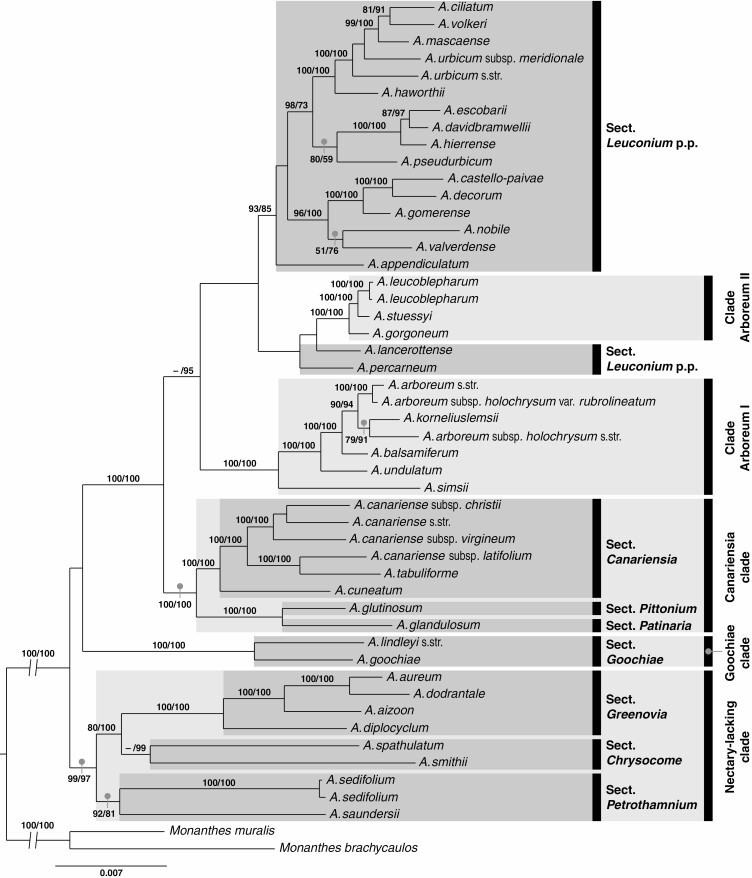
Maximum likelihood phylogeny of *Aeonium* inferred from concatenated supermatrices (concatML). Numbers above branches indicate bootstrap values (only shown if at least one of them is ≥75) obtained from the complete dataset of all 4280 loci (all/RAxML; values to the left) and from the dataset only comprising the 2218 loci in the 320- to 500-nt length range (320–500/RAxML; values to the right). Dashes (–) signify clades that were supported in 320–500/RAxML but not recovered in all/RAxML. Branches arising directly from the root were shortened (branch length = 0.019) for the purpose of better visibility. Clade names and affiliations to sections are indicated to the right of taxon names. Topology and branch lengths correspond to the results obtained with the 320–500 dataset. p.p., pro parte (partly).

**Fig. 3.**
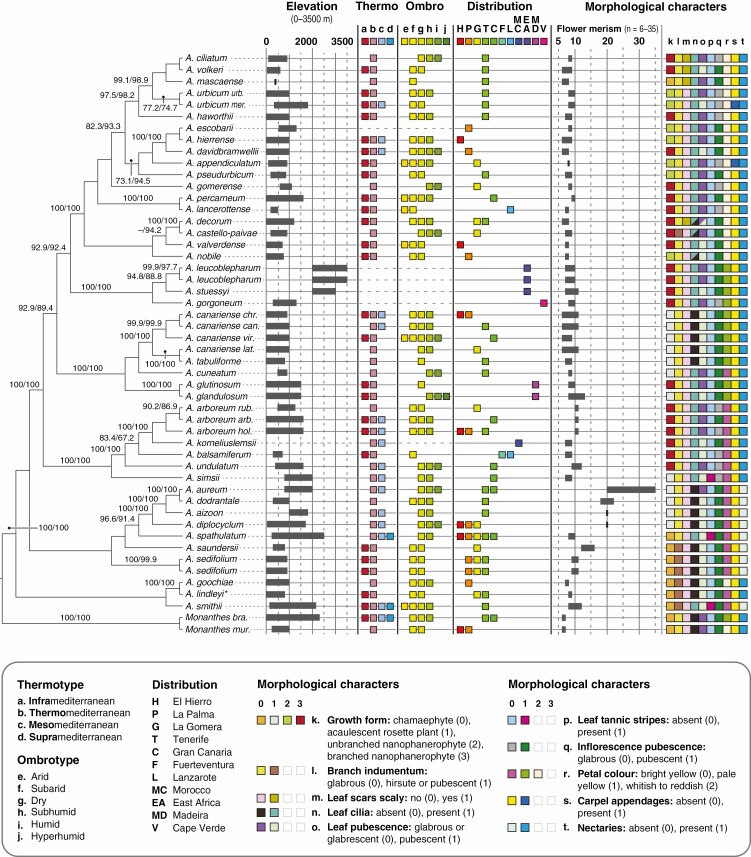
Species tree of *Aeonium* inferred using ASTRAL-III. Numbers above branches indicate bootstrap values (only shown if at least one of them is ≥75) obtained from the species-tree analysis of all 4280 individual RAxML gene trees and from those 2218 gene trees inferred from loci in the 320- to 500-nt length range (left and right values, respectively). The dash (–) signifies a clade that was supported in 320–500/ASTRAL but not recovered in all/ASTRAL. The topology corresponds to the results obtained with the 320–500 dataset. Information about geographical distribution, elevational range across the distribution area and thermo- and ombrotype is given to the right of taxon names. Missing data with respect to elevation, thermo- and ombrotype is indicated with dashed horizontal lines. Further to the right, morphological character states are coded and specified by the inset figure legend below. *A. urbicum urb.*, *Aeonium urbicum* subsp. *urbicum*; *A. urbicum mer.*, *A. urbicum* subsp. *meridionale*; *A. canariense chr.*, *A. canariense* subsp. *christii*; *A. canariense can.*, *A. canariense* subsp. *canariense*; *A. canariense vir.*, *A. canariense* subsp. *virgineum*; *A. canariense lat.*, *A. canariense* subsp. *latifolium*; *A. arboreum rub.*, *A. arboreum* subsp. *holochrysum* var. *rubrolineatum*; *A. arboreum arb.*, *A. arboreum* subsp. *arboreum*; *A. arboreum hol.*, *A. arboreum* subsp. *holochrysum* var. *holochrysum*; *Monanthes bra.*, *M. brachycaulos*; *Monanthes mur.*, *M. muralis*. *A. lindleyi* *Aeonium lindleyi s.l., including both subspecies.

**Fig. 4.**
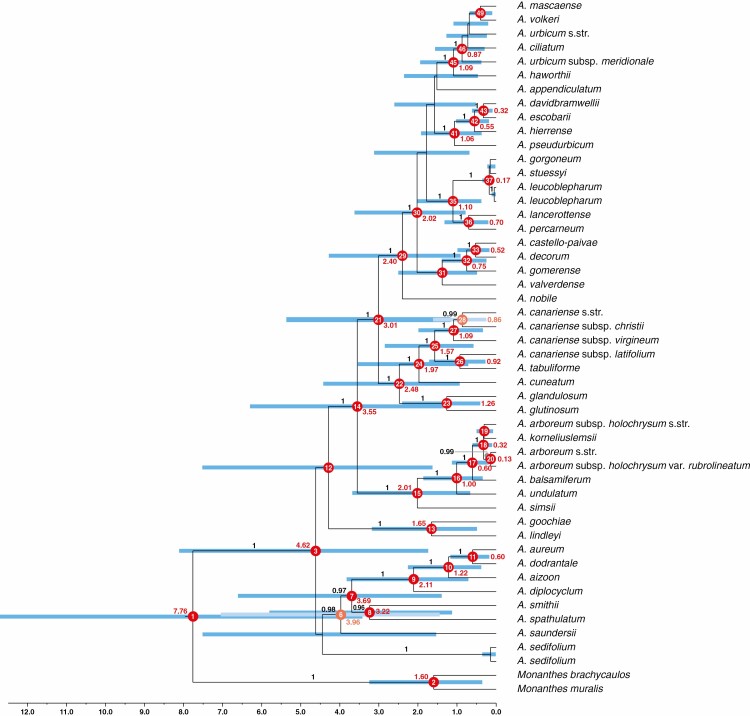
Chronogram of *Aeonium* inferred using BEAST, based on the sequence dataset of those 357 loci in the 320- to 500-nt length range that had sequence information for at least 20 different samples (referred to as the min20tax dataset in the text). The topology inferred from the 320–500/RAxML analysis (Fig. 2) was used as starting tree for this BEAST analysis. Only posterior probabilities ≥0.85 are shown above branches. Mean age estimates are given for each supported node (posterior probability ≥0.95) in red font, and the blue error bars indicate the respective 95 % highest posterior density (HPD) intervals. For nodes 6 and 28, age estimates and error bars received paler colours for a better contrast with neighbouring nodes. The scale shows time in million years before present. See also Table 2 for a summary of the stem and crown age estimates of well-supported clades.

Raw Euclidean distances (REDs; Lloyd, 2016) were calculated as a measure of morphological and ecological divergence between well-supported sister lineages. More precisely, we identified those nodes that were present in all phylogenetic analyses (including the dating analysis) and received high support (BS = 100 and posterior probability ≥0.99) in at least one of these analyses. For these nodes, we separately calculated the morphological and ecological REDs between the respective sister lineages using the matrix of morphological characters (see section Compilation of morphological, ecological and species-occurrence data, above) and habitat characteristics (i.e. elevation, thermotype and ombrotype), respectively. The internal topology of the well-supported Tenerifean subclade of sect. *Leuconium* (i.e. *A. ciliatum* through *A. haworthii* in Figs 2 and 3, nodes 46–49 in Fig. 4) was inconsistently recovered across our phylogenetic analyses, but diversification in this subclade was reconstructed to have happened within Tenerife (Supplementary Data File S4). Therefore, we calculated morphological and ecological REDs in this subclade separately for each of the different topologies as recovered by our concatML (Fig. 2), ASTRAL (Fig. 3) and dating analyses (Fig. 4). Finally, the magnitude of morphological and ecological REDs was compared pairwise between those nodes that, according to the results of our BSM analysis, were associated with intra-island diversification on the one hand and those nodes that were associated with inter-island diversification on the other hand (two-sided *t*-test).

## RESULTS

### Sequencing, parameter optimization in ipyrad and locus properties of the final assemblies

The Illumina sequencing reactions yielded a total of 13 921 303 paired-end reads, which is equivalent to an average number of 272 967 (±146 466) paired-end reads per sample. Of these, 13 914 286 (i.e. 99.95 %) reads passed quality filtering. *Aeonium undulatum*, *A. balsamiferum* and *A. gorgoneum* had the highest number of reads (680 738, 623 623 and 621 070, respectively) while *A. lindleyi* subsp. *viscatum* and *A. nobile* had the lowest number of reads (32 710 and 88 491, respectively).

The box- and scatterplots of the relevant assembly metrics as obtained from ipyrad after ISC against the tested CTs (Supplementary Data Fig. S1) revealed similar patterns for samples treated as diploid and tetraploid. For both groups, an onset of exponential increase in the number of clusters (Supplementary Data Fig. S1, clusters_total) became apparent at CT values between 0.92 and 0.94, indicating incipient undermerging in this CT range. In the same range, the decrease in number of rejected clusters due to high heterozygosity (Supplementary Data Fig. S1, filtered_by_maxH) was steepest, and the decreasing slope of average read depth (Supplementary Data Fig. S1, avg_depth_total) gained steepness. Maximum heterozygosity was reached at CT 0.92 for the tetraploid and at CT 0.93 for the diploid samples. Thus, for the tetraploids CT 0.92 was identified as the most suitable clustering threshold, and the same CT value was also chosen for the diploids because the other metrics indicated that undermerging had an impact on the ISC of diploids at CT 0.93. After merging all samples of the ISC runs performed with a CT value of 0.92, the results of the BSC test (Supplementary Data File S5) showed the characteristic ‘hockey-stick signal’ (Paris *et al.*, 2017) when the number of new polymorphic loci was plotted against CT, with an initial increase again at CT 0.92. This signal coincided with a minimum of missingness in the assembled dataset (Supplementary Data File S5, 73.17 % missing sites). Although the cumulative number of single-nucleotide polymorphisms (SNPs) and PISs both reached a maximum at a CT value of 0.93, this was not preferred over CT 0.92 for the same reason as in the ISC of diploid samples.

Because *A. lindleyi* subsp. *viscatum* only had 24 loci in the assembly, it was removed from the dataset for the final assembly. After removing the loci of the plastome as well as those loci without PIS, the resulting assembly comprised 50 samples and 4280 loci with a total length of 1 529 544 nt and an average length of 357.4 nt per locus (total length range 49–617 nt). The numbers of SNPs and PISs amounted to 89 093 (20.8 ± 13.3 SNPs per locus) and 49 209 (11.5 ± 9.4 PIS per locus), respectively. With an average of 11.2 samples per locus, the average missingness per locus was relatively high (77.6 ± 17.5 %). When only the locus length range of 320–500 nt was retained (320–500 dataset; see Materials and methods section), the number of loci was reduced to 2218 (51.8 % of the total number of loci) and the total length of the assembly was 884 786 nt (398.9 ± 49.8 nt per locus). This reduced assembly had a higher number of SNPs and PISs per locus (22.8 ± 13.1 and 13.1 ± 10.4, respectively) and a slightly lower average missingness per locus (76.8 ± 18.7 %) than the dataset comprising all loci.

### *Phylogenetic relationships and age estimates for the diversification of* Aeonium

All four phylogenies, i.e. all/RAxML, 320–500/RAxML (Fig. 2), all/ASTRAL and 320–500/ASTRAL (Fig. 3), strongly supported *Aeonium* sect. *Goochiae* as monophyletic, i.e. the Goochiae clade (*A. goochiae* and *A. lindleyi* in Fig. 2). This clade was also well supported when *A. lindleyi* subsp. *viscatum* was included (Supplementary Data Fig. S2) in spite of its very low locus coverage (see section Sequencing, parameter optimization in ipyrad and locus properties of the final assemblies, above). In this latter analysis the two subspecies of *A. lindleyi* were supported as sister to each other. However, the position of the Goochiae clade relative to other lineages differed between analyses. While it was resolved as the earliest-branching lineage of *Aeonium* in all/RAxML, it formed a polytomy with two larger clades in 320–500/RAxML (Fig. 2). The smaller of these two clades (i.e. the nectary-lacking clade in Fig. 2) was not recovered by either of the ASTRAL analyses (Fig. 3). It comprises sects *Chrysocome*, *Greenovia* and *Petrothamnium*. Section *Greenovia* was well supported as monophyletic, and relationships among the four species of this section were consistently well resolved in all analyses. Section *Petrothamnium* was only monophyletic in both concatML phylogenies, and sect. *Chrysocome* was only supported as monophyletic in 320–500/RAxML (Fig. 2). In summary, sects *Goochiae* and *Greenovia* were concordantly supported as monophyletic sections, while the inferred relationships of the remaining taxa in the basal part of the phylogeny differed between analyses (Figs 2 and 3). In this part of the phylogeny, the only taxon recovered in conflicting positions was *A. spathulatum*, which was supported as sister to sect. *Greenovia* in the ASTRAL analyses (Fig. 3) but sister to *A. smithii* in 320–500/RAxML (Fig. 2). Possible causes for incongruence among our phylogenies, such as incomplete lineage sorting or hybridization, were not explored in depth. A search for potential hybrids among our samples of *Aeonium* using HyDe (Blischak *et al.*, 2018) did not return any significant test results (not shown).

The larger clade, *A. ciliatum* through *A. glandulosum* in Fig. 2 and *A. ciliatum* through *A. simsii* in Fig. 3, was always well supported and contains species of the remaining sections, i.e. sects *Aeonium*, *Canariensia*, *Leuconium*, *Patinaria* and *Pittonium*. In this clade, the different analyses resolved different subclades as the earliest-branching lineage. The phylogenies inferred from species-tree analyses in ASTRAL-III (Fig. 3) supported a clade comprising all Canarian taxa of sect. *Aeonium* plus *A. korneliuslemsii* from Morocco as the earliest-diverging lineage (*A. arboreum rub.* through *A. simsii* in Fig. 3, i.e. clade Arboreum I in Fig. 2). In the concatML phylogenies (Fig. 2), the position of earliest-diverging lineage within this larger clade was taken by an always well-supported subclade comprising all taxa of sects *Canariensia*, *Patinaria* and *Pittonium* (*A. canariense* subsp. *christii* through *A. glandulosum* in Fig. 2). Within this subclade, henceforth referred to as the Canariensia clade, the monospecific sects *Patinaria* and *Pittonium* (i.e. *A. glandulosum* and *A. glutinosum*, respectively) were most closely related to each other and sister to the monophyletic sect. *Canariensia* in all analyses. In the all/RAxML phylogeny, both clade Arboreum I and the Canariensia clade were well supported as clades, but formed a polytomy with a small clade, i.e. clade Arboreum II, comprising the East African and Cape Verde taxa of sect. *Aeonium* (*A. leucoblepharum* through *A. gorgoneum* in Fig. 2), two single species (*A. lancerottense* and *A. percarneum*; both sect. *Leuconium*) and one large clade (*A. ciliatum* through *A. appendiculatum* in Fig. 2) containing taxa only of sect. *Leuconium*. Clade Arboreum II was also supported in all other analyses, and in both ASTRAL phylogenies all species of sect. *Leuconium* were well supported as a clade, i.e. the Leuconium clade (Fig. 3). To summarize relationships in the larger clade above the nectary-lacking clade/grade and the Goochiae clade, the Canariensia clade comprised a monophyletic sect. *Canariensia* that was supported as sister to the Madeiran species *A. glandulosum* and *A. glutinosum*. Two distinct clades with representatives of sect. *Aeonium* were recovered, one containing the Canarian species and *A. korneliuslemsii* (clade Arboreum I), the other containing the East African and Cape Verde species (clade Arboreum II). The Canariensia clade was inconsistently supported as either sister to a clade including both these Arboreum clades plus sect. *Leuconium* (concatML analyses; Fig. 2) or only clade Arboreum II plus sect. *Leuconium* (ASTRAL analyses; Fig. 3). Section *Leuconium* was in turn supported as monophyletic only in the ASTRAL analyses (Fig. 3), while it formed an unsupported paraphyletic group in relation to clade Arboreum II in the concatML analyses (Fig. 2).

The chronogram of the *Aeonium* phylogeny obtained from the BEAST analysis of the min20tax dataset using the topology of the 320–500/RAxML analysis as starting tree is shown in Fig. 4. The corresponding age estimates for nodes that were well supported in all phylogenetic analyses are summarized in Table 2. The BEAST analysis of the same min20tax dataset but with the topology of the 320–500/ASTRAL analysis as starting tree (Supplementary Data Fig. S3) resulted in a topology identical and divergence time estimates nearly identical to those obtained when using the 320–500/RAxML topology as starting tree. Therefore, only the results of the BEAST analysis with the 320–500/RAxML topology as starting tree are described and discussed. The divergence between *Aeonium* and *Monanthes* was dated to the Upper Miocene or Pliocene, 7.76 myr (95 % HPD = 3.42–12.7 myr), and thus towards the younger range of the prior for the root (11.62 myr; 95 % HPD = 6.56–17.1 myr). The onset of diversification of *Aeonium* was dated to 4.62 myr (95 % HPD = 1.74–8.11 myr), a time when El Hierro and La Palma had not yet formed (van den Bogaard, 2013). Mean age estimates for the diversification within sects *Chrysocome* and *Petrothamnium* were all older than 3 myr (Fig. 4), and the diversification of all other sections started later. The mean age estimates for divergence between these sections and sect. *Greenovia* (node 7 in Fig. 4) as well as between clade Arboreum I, the Canariensia clade and the larger clade comprising sect. *Leuconium* and clade Arboreum II (nodes 14 and 21 in Fig. 4) ranged between 3 and 4 myr (95 % HPD = 1.16–6.61 myr). The split of sects *Patinaria* and *Pittonium* from sect. *Canariensia* and thus the colonization of Madeira was dated to 2.48 myr ago (95 % HPD = 0.93–4.42 myr ago). The colonization of the African continent [1.1 myr ago (95 % HPD = 0.37–2.01 myr ago) by the East African lineage giving rise to *A. leucoblepharum* and *A. stuessyi*, and 0.27 myr ago (95 % HPD = 0.08–0.50 myr ago) by the Moroccan *A. korneliuslemsii*] and of the Cape Verde Islands (0.1 myr ago; 95 % HPD = 0.01–0.22 myr ago) took place much later. The onset of diversification in sect. *Leuconium* was dated to 2.40 myr ago (95 % HPD = 0.91–4.28 myr ago). This diversification, as well as the incipient diversification of sects *Greenovia* (2.11 myr ago; 95 % HPD = 0.71–3.82 myr ago) and *Canariensia* (1.97 myr ago; 95 % HPD = 0.71–3.54 myr ago) and clade Arboreum I (2.01 myr ago; 95 % HPD = 0.66–3.68 myr ago), took place after the onset of the Pleistocene glaciation cycles (Gibbard *et al.*, 2010). Section *Goochiae* (1.65 myr ago; 95 % HPD = 0.49–3.18 myr ago) and the Madeiran species *A. glandulosum* and *A. glutinosum* (1.26 myr ago; 95 % HPD = 0.40–2.4 myr ago) diverged later.

**Table 2.** Age estimates in million years for all supported nodes (posterior probability ≥0.95 unless otherwise specified) of the chronogram of *Aeonium* (Fig. 4). For clade names see Fig. 2

Node number^a^	Clade name	Node age (95 % confidence interval)
1	*Aeonium* + *Monanthes*	7.76 (3.42–12.70)
3	*Aeonium*	4.62 (1.74–8.11)
6	*A. saundersii* + sects *Chrysocome* and *Greenovia*	3.96 (1.44–7.05)
7	Sections *Chrysocome* + *Greenovia*	3.69 (1.39–6.61)
8	Section *Chrysocome*	3.22 (1.12–5.80)
9	Section *Greenovia*	2.11 (0.71–3.82)
10	*A. aizoon*, *A. aureum* + *A. dodrantale*	1.22 (0.38–2.25)
11	*A. aureum* + *A. dodrantale*	0.60 (0.17–1.17)
12c	Goochiae clade, Canariensia clade, sects *Aeonium* and *Leuconium*	4.23 (1.63–7.52)
13	Goochiae clade	1.65 (0.49–3.18)
14	Canariensia clade + sects *Aeonium* and *Leuconium*	3.55 (1.34–6.30)
15	Clade Arboreum I	2.01 (0.66–3.68)
16	*A. undulatum*, *A. balsamiferum*, *A. korneliuslemsii* + *A. arboreum* s.l.	1.00 (0.34–1.86)
17	*A. balsamiferum*, *A. korneliuslemsii* + *A. arboreum* s.l.	0.60 (0.19–1.13)
18	*A. korneliuslemsii* + *A. arboreum* s.l.	0.32 (0.10–0.60)
19	*A. korneliuslemsii* + *A. arboreum* subsp. *holochrysum* var. *holochrysum*	0.27 (0.08–0.50)
20	*A. arboreum* subsp. *arboreum* + *A. arboreum* subsp. *holochrysum* var. *rubrolineatum*	0.13 (0.02–0.28)
21	Canariensia clade, clade Arboreum II + sect. *Leuconium*	3.01 (1.16–5.37)
22	Canariensia clade	2.48 (0.93–4.42)
23	Sects *Patinaria* + *Pittonium*	1.26 (0.40–2.40)
24	*A. cuneatum*, *A. canariense* s.l. + *A. tabuliforme*	1.97 (0.71–3.54)
25	*A. canariense* s.l. + *A. tabuliforme*	1.57 (0.57–2.85)
26	*A. canariense* subsp. *latifolium* + *A. tabuliforme*	0.92 (0.27–1.71)
27	*A. canariense* without *A. canariense* subsp. *latifolium*	1.09 (0.33–1.99)
28	*A. canariense* subsp. *christii* + *A. canariense* subsp. *canariense*	0.86 (0.25–1.61)
29	Clade Arboreum II + sect. *Leuconium*	2.40 (0.91–4.28)
30b	Clade Arboreum II + sect. *Leuconium* without *A. nobile*	2.02 (0.78–3.62)
31b	*A. valverdense*, *A. gomerense*, *A. castello-paivae* + *A. decorum*	1.37 (0.48–2.50)
32	*A. gomerense*, *A. castello-paivae* + *A. decorum*	0.75 (0.24–1.39)
33	*A. castello-paivae* + *A. decorum*	0.52 (0.17–0.99)
35	*A. lancerottense*, *A. percarneum*, clade Arboreum II	1.10 (0.37–2.01)
36	*A. lancerottense* + *A. percarneum*	0.70 (0.20–1.32)
37	Clade Arboreum II	0.17 (0.04–0.35)
41	*A. pseudurbicum*, *A. hierrense*, *A. davidbramwellii* + *A. escobarii*	1.06 (0.37–1.92)
42	*A. hierrense*, *A. davidbramwellii* + *A. escobarii*	0.55 (0.18–1.02)
43	*A. davidbramwellii* + *A. escobarii*	0.32 (0.09–0.62)
45	*A. haworthii*, *A. urbicum* s.l., *A. ciliatum*, *A. volkeri* + *A. mascaense*	1.09 (0.37–1.94)
46b	*A. urbicum* s.l., *A. ciliatum*, *A. volkeri* + *A. mascaense*	0.87 (0.29–1.56)
49b	*A. volkeri* + *A. mascaense*	0.35 (0.10–0.68)

^a^Node numbers as specified in Fig. 4.

^b^Posterior probability for this node or for at least one of the two subordinate nodes <0.95, but ≥0.85.

### *Biogeography, co-occurrence and hybridization of* Aeonium *species*

**Fig. 5.**
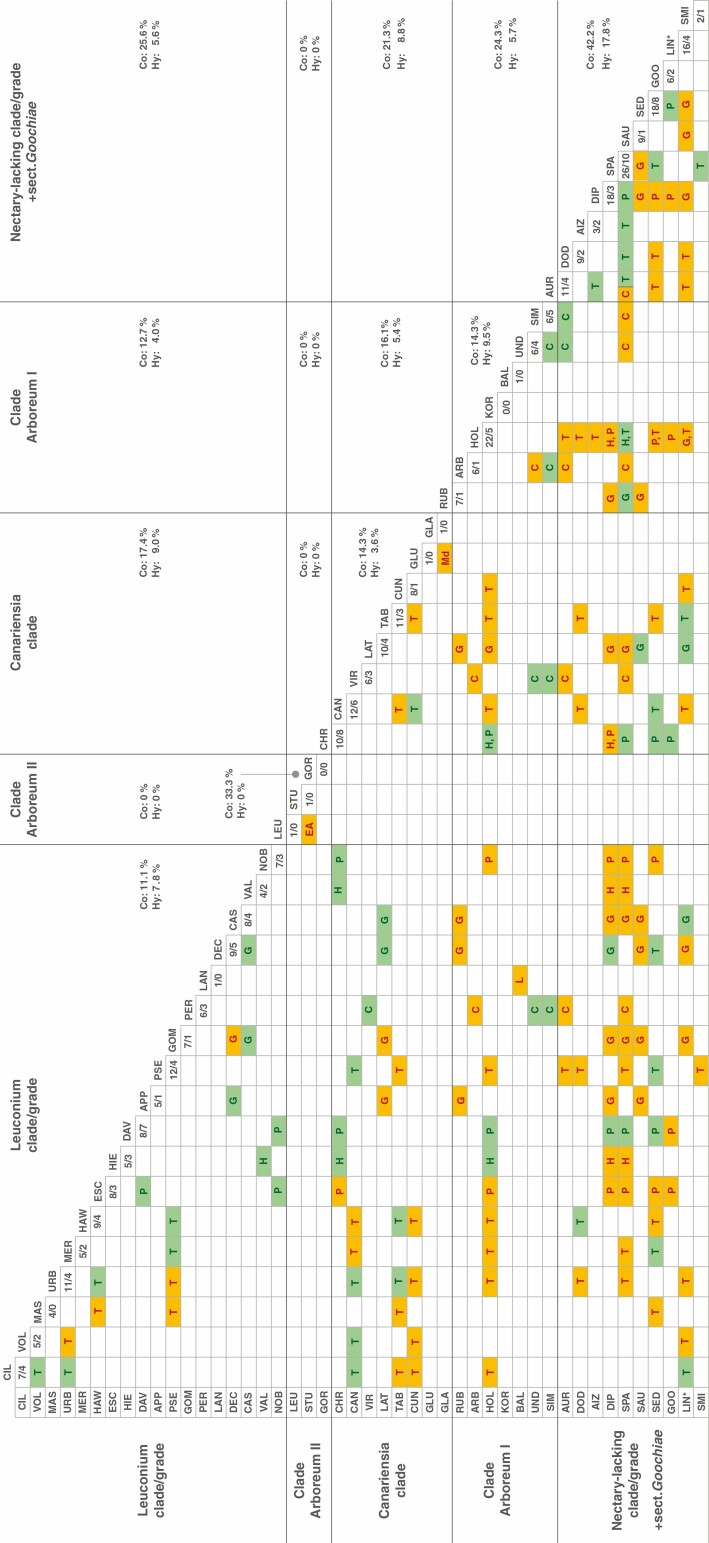
Co-occurrence matrix of all species and subspecies of *Aeonium*. Taxa are sorted in clades (see Fig. 2 for clade names) and their names are abbreviated by the first three letters of their species/subspecies/variety epithet. Boxes filled in ochre indicate co-occurrence of the two taxa defining the coordinates of the box, boxes filled in green indicate published natural hybrids between the two respective taxa. Letters in coloured boxes specify where the co-occurrence of two taxa or their hybrids are located; for the geographical codes of these letters see Table 1 or Fig. 3. Numbers in the diagonal boxes below the abbreviated taxon names summarize the number of co-occurrences and number of natural hybrids with other *Aeonium* taxa (left and right of the slash, respectively) for each taxon. Percentages marked ‘Co’ and ‘Hy’ indicate the proportions of co-occurrences and natural hybrids, respectively, for each pairing of two clades, where the number of possible pairings between all taxa of the two respective clades is considered 100 %. LIN^*^, *Aeonium lindleyi* s.l., including both subspecies.

The co-occurrence matrix for all species of *Aeonium* is shown in Fig. 5, which also contains information on published natural hybrids between any two of all species. Compared with other clades/grades of the *Aeonium* phylogeny, taxa of the basal nectary-lacking group plus sect. *Goochiae* co-occur and hybridize with each other to the greatest extent (42.2 and 17.8 %, respectively). The same applies to the proportion of co-occurrence and hybridization between these taxa and taxa of other clades/grades (on average 17.8 and 5 %, respectively; Fig. 5). Disregarding the exclusively extra-Canarian clade Arboreum II, the basal grade again co-occurs and hybridizes with the highest proportion of species of other clades or grades (on average 23.7 and 6.7 %, respectively), followed by the Leuconium clade/grade (18.6 and 6.2 %, respectively), the Canariensia clade (18.3 and 7.7 %, respectively) and clade Arboreum I (17.7 and 5 %, respectively). Concerning co-occurrence and hybridization of species within individual clades/grades, the basal grade is followed by clade Arboreum I (14.3 and 9.5 %, respectively), the Canariensia clade (14.3 and 3.6 %, respectively) and the Leuconium clade/grade (11.1 and 7.8 %, respectively; Fig. 5).

The co-occurrence matrix (Fig. 5) also revealed that Gran Canaria has the highest proportion of co-occurring *Aeonium* taxa among the western Canary Islands (i.e. the Canary Islands except Fuerteventura and Lanzarote). More specifically, every taxon of *Aeonium* that occurs on Gran Canaria can co-occur with every other taxon of *Aeonium*, at least at some place, on this island, meaning a co-occurrence rate of 100 %. This is followed by La Palma (88.9 %), El Hierro (80 %), La Gomera (70.9 %) and Tenerife (43.3 %). The two *Aeonium* species of Lanzarote co-occur but do not hybridize, and Fuerteventura is home to only one *Aeonium* species (Table 1). Natural hybrids of *Aeonium* taxa are found on all western Canary Islands (Fig. 5), with the highest rate of hybridizing taxa on Gran Canaria (42.9 %), followed by La Palma (42.7 %), El Hierro (40.0 %), La Gomera (18.2 %) and Tenerife (15.2 %).

The AAR analysis in BioGeoBEARS identified BAYAREALIKE+J as the best-fitting model (LnL = −173.4; AICc_wt = 1.0; Table 3). This was followed by the DEC+J model receiving the second highest scores (LnL = −180.9; AICc_wt = 5.0 × 10^−4^). The BSM analysis revealed founder events (32.9 %, interpreted as inter-island speciation), anagenetic dispersal events (33.5 %, interpreted as inter-island dispersal without speciation) and sympatric diversification events (33.6 %, interpreted as intra-island speciation) as biogeographical processes that were more or less equally frequent in the evolution of *Aeonium* (Table 4).

**Table 3.** Comparison of parameters obtained from different models in the ancestral area reconstruction analysis in BioGeoBEARS. The results for BAYAREALIKE+J, i.e. the model with the best fit based on LnL and AICc_wt, are highlighted in bold

Model	LnL	*d*	*e*	*j*	AICc	AICc_wt
DEC	−197.4	0.051	0.180	0	399.0	1.2 × 10^−10^
DEC+J	−180.9	0.026	0.042	0.049	368.4	5.0 × 10^−4^
DIVALIKE	−195.8	0.054	0.140	0	395.9	5.6 × 10^−10^
DIVALIKE+J	−184.0	0.030	0.043	0.041	374.6	2.4 × 10^−5^
BAYAREALIKE	−207.8	0.055	0.520	0	419.9	3.4 × 10^−15^
**BAYAREALIKE+J**	**−173.4**	**0.021**	**0.044**	**0.050**	**353.3**	**1.0**

*d*, dispersal rate.

*e*, extinction rate.

*j*, rate of founder-event speciation (jump dispersal).

**Table 4.** Absolute and percentage mean incidence (± standard deviation) of biogeographical events recorded in 50 individual BSMs simulated for *Aeonium* under the BAYAREALIKE+J model. Cladogenetic dispersal corresponds to founder-event speciation (parameter *j* in BioGeoBEARS). Cladogenesis in sympatry and anagenetic dispersal correspond to parameters *y* and *d* in BioGeoBEARS, respectively. Note that this model does not allow for intra-range (subset) speciation and for vicariance

	Dispersal	Sympatry	Sum
Cladogenesis	24.24 (± 1.61); 32.9%	24.76 (± 1.61); 33.6%	49.00 (± 0.00); 66.5%
Anagenesis	24.64 (± 3.02); 33.5%	–	24.64 (± 3.02); 33.5%
Sum	48.88 (± 3.24); 66.4%	24.76 (± 1.61); 33.6%	73.64 (± 3.02); 100%

**Fig. 6.**
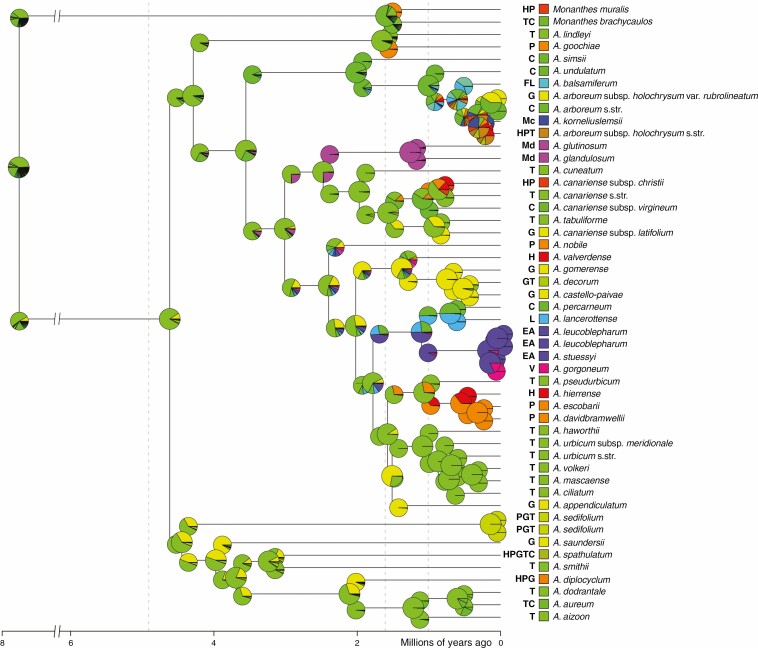
Ancestral area reconstruction of the dated *Aeonium* phylogeny (Fig. 4) obtained using the BAYAREALIKE+J model in BioGeoBEARS. Pie charts indicate the relative probability of each region or combination of regions for each node and each branch, and rectangles at the tips indicate the recent distribution area or combination of regions for each sampled taxon of the phylogeny. The branches arising directly from the root were shortened for better visibility. Dashed vertical lines represent the ages of Madeira (~5 myr), La Palma ( 1.7 myr) and El Hierro ( 1.1 myr). For the geographical code of letters at the tree tips see Table 1 or Fig. 3.

**Fig. 7:**
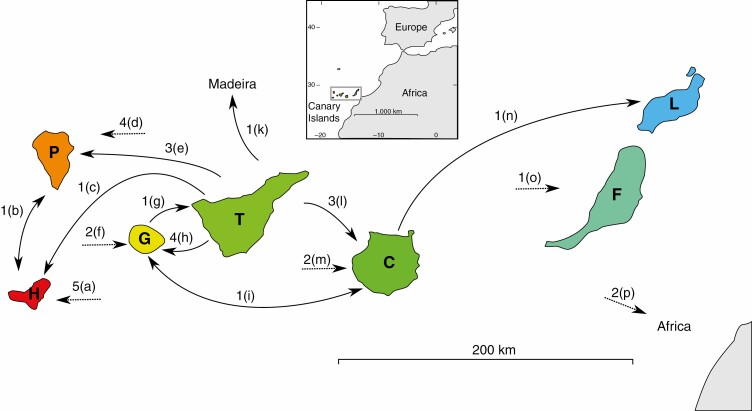
Topology of the Canary Islands off the Atlantic coast of North Africa (inset map) and dispersal paths between islands in the evolution of *Aeonium* that were inferred by the majority of the BSMs. Arrowheads indicate directionality of dispersals, and arrows with arrowheads on both ends indicate ambiguity about directionality. Dotted arrows indicate dispersals with ambiguous region of origin. Numbers above arrows correspond to the number of times a certain dispersal path was taken. Letters in brackets correspond to the nodes and lineages listed below (see Fig. 2 for clade names and Fig. 4 for node numbers) that followed the corresponding dispersal path. For the letter code of island names see Table 1 or Fig. 3. (a) Node 17; *Aeonium canariense* subsp. *christii*, *A. diplocyclum*, *A. spathulatum*, *A. valverdense*. (b) Node 42. (c) Node 28. (d) Node 17; *Aeonium canariense* subsp. *christii*, *A. diplocyclum*, *A. spathulatum*. (e) Nodes 13 and 29; *Aeonium sedifolium*. (f) Node 32; *Aeonium lindleyi* subsp. *viscatum*. (g) *Aeonium decorum*. (h) Nodes 9 and 26; *Aeonium appendiculatum*, *A. sedifolium*. (i) Node 20. (j) *Aeonium arboreum* subsp. *holochrysum* var. *holochrysum*. (k) Node 22. (l) Nodes 14 and 27; *Aeonium aureum*. (m) *Aeonium percarneum*, *A. spathulatum*. (n) Node 36. (o) *Aeonium balsamiferum*. (p) Node 35; *Aeonium korneliuslemsii*.

Tenerife was inferred as the ancestral area at the crown node of *Aeonium* (Fig. 6; node 3 in Fig. 4) with a probability of 82.6 %. The BSM analysis furthermore identified Tenerife as by far the most important starting point for dispersal events, as, on average, 41.73 % of all recorded cladogenetic and anagenetic dispersal events (i.e. parameters *j* and *d* in BioGeoBEARS, respectively) originated from Tenerife (Table 5). Most of the remaining dispersal events had their origin on La Gomera (14.73 %), Gran Canaria (12.32 %), La Palma (10.02 %) and El Hierro (7.82 %; Table 5). As receivers of dispersal events, islands were much closer to each other in frequency. La Palma acted as sink in 18.94 %, La Gomera in 16.41 %, Gran Canaria in 16.12 %, El Hierro in 14.73 % and Tenerife in 10.72 % of all dispersal events (Table 5). All inter-island paths of dispersal that were assigned to two specific islands by the majority of the BSM simulations are summarized in Fig. 7. Following Tenerife where *Aeonium* most probably originated (see above), La Gomera has been colonized for the longest time as suggested by 44 of 50 BSMs in which the ancestral lineage of *A. saundersii* (node 6 in Fig. 4 and Table 2; median age estimate: 3.96 myr) was inferred as the first inhabitant of La Gomera (Supplementary Data File S4). Gran Canaria was most probably reached for the first time between 3.55 myr ago (95 % HPD = 1.34–6.3 myr ago) and 2.01 myr ago (95 % HPD = 0.66–3.68 myr ago), because in 38/50 BSMs Gran Canaria was colonized by the most recent common ancestor (MRCA) of clade Arboreum I (node 14), and all BSMs inferred the ancestral lineage of *A. simsii* (node 15) as colonizer of Gran Canaria (Supplementary Data File S4). The BSM analysis further suggests that La Palma and El Hierro were first colonized only shortly after their emergence above sea level (~1.7 and 1.1 myr ago, respectively; van den Bogaard, 2013). Thus, La Palma was first reached by the ancestral lineage of *A. goochiae* in 48/50 BSMs (1.65 myr; 95 % HPD = 0.49–3.18 myr), corresponding to node 13 (Fig. 4 and Table 2). El Hierro was first reached either by the ancestral lineage of the well-supported clade of *A. hierrense*, *A. davidbramwellii* and *A. escobarii* (1.06 myr; 95 % HPD = 0.37–1.92 myr) in 26/50 BSMs, or by the ancestral lineage of *A. canariense* subsp. *christii* (0.86 myr; 95 % HPD = 0.25–1.61 myr) in an additional 14 BSMs (Supplementary Data File S4), corresponding to nodes 41 and 28, respectively. Colonization of the eastern Canary Islands (i.e. Fuerteventura and Lanzarote) by *Aeonium* happened relatively late and at least twice independently, namely 0.7 myr ago (95 % HPD = 0.2–1.32 myr ago) by the lineage giving rise to *A. lancerottense* and 0.6 myr ago (95 % HPD = 0.19–1.13 myr ago) by the lineage giving rise to *A. balsamiferum* (nodes 36 and 17, respectively). However, in 31/50 BSMs one of the eastern Canary Islands was reached earlier than this, and in most of these cases at least one of the immediate parental lineages (i.e. nodes 35 and 16, respectively) was simulated as native to Fuerteventura and/or Lanzarote (Supplementary Data File S4). The African continent was back-colonized at least twice independently, i.e. by dispersal of *A. korneliuslemsii* to Morocco 0.27 myr ago (95 % HPD = 0.08–0.50 myr ago), and dispersal of the MRCA of clade Arboreum II to East Africa and the southwestern part of the Arabian Peninsula between 0.17 and 1.10 myr ago (95 % HPD = 0.04–2.01 myr ago). Finally, the Cape Verde Islands were reached from the African continent by the ancestor of *A. gorgoneum* after dispersal of clade Arboreum II to Africa (Fig. 6). However, the relationships among the three species of clade Arboreum II were unsupported in our dated phylogeny (Fig. 4; see Discussion section).

**Table 5:** Number of cladogenetic + anagenetic dispersal counts (mean ± standard deviation) between islands/regions averaged across 50 individual BSMs simulated for *Aeonium* under the BAYAREALIKE+J model. Direction of dispersal events is from the row state to the column state. At the end of each row the average number (absolute and relative) of dispersal events originating from the corresponding island/region is given. At the bottom of each column, the respective number of dispersal events terminating on the corresponding island/region is given. C, Gran Canaria; EA, East Africa; F, Fuerteventura; G, La Gomera; H, El Hierro; L, Lanzarote; Mc, Morocco; Md, Madeira; P, La Palma; T, Tenerife; V, Cape Verde

	H	P	G	T	C	F	L	Mc	EA	Md	V	Sum
H	–	1.74 (± 0.90)	0.36 (± 0.56)	0.28 (± 0.50)	0.54 (± 0.79)	0.18 (± 0.44)	0.20 (± 0.40)	0.46 (± 0.54)	0.02 (± 0.14)	0.02 (± 0.14)	0.02 (± 0.14)	3.82 7.82%
P	2.60 (± 0.97)	–	0.44 (± 0.73)	0.40 (± 0.61)	0.60 (± 0.64)	0.12 (± 0.33)	0.20 (± 0.45)	0.42 (± 0.50)	0.06 (± 0.24)	0.06 (± 0.24)	0.00 (± 0.00)	4.90 10.02%
G	1.22 (± 0.74)	1.52 (± 0.93)	–	2.36 (± 1.45)	1.10 (± 0.86)	0.18 (± 0.44)	0.18 (± 0.39)	0.14 (± 0.40)	0.20 (± 0.45)	0.12 (± 0.33)	0.18 (± 0.39)	7.20 14.73%
T	2.38 (± 1.32)	3.86 (± 1.39)	5.70 (± 1.64)	–	4.56 (± 1.05)	0.48 (± 0.58)	0.74 (± 0.88)	0.46 (± 0.73)	0.80 (± 0.73)	1.10 (± 0.68)	0.32 (± 0.62)	20.40 41.73%
C	0.56 (± 0.58)	1.34 (± 1.00)	1.00 (± 0.88)	1.38 (± 1.38)	–	0.16 (± 0.42)	0.74 (± 0.66)	0.04 (± 0.20)	0.54 (± 0.65)	0.20 (± 0.45)	0.06 (± 0.24)	6.02 12.32%
F	0.06 (± 0.24)	0.08 (± 0.27)	0.04 (± 0.20)	0.06 (± 0.31)	0.14 (± 0.40)	–	0.48 (± 0.54)	0.00 (± 0.00)	0.04 (± 0.20)	0.02 (± 0.14)	0.02 (± 0.14)	0.94 1.92%
L	0.08 (± 0.27)	0.28 (± 0.45)	0.12 (± 0.33)	0.20 (± 0.40)	0.50 (± 0.51)	0.54 (± 0.58)	–	0.04 (± 0.20)	0.24 (± 0.43)	0.02 (± 0.14)	0.00 (± 0.00)	2.02 4.13%
Mc	0.00 (± 0.00)	0.10 (± 0.36)	0.08 (± 0.34)	0.06 (± 0.24)	0.08 (± 0.27)	0.00 (± 0.00)	0.02 (± 0.14)	–	0.00 (± 0.00)	0.04 (± 0.20)	0.00 (± 0.00)	0.38 0.78%
EA	0.14 (± 0.35)	0.10 (± 0.30)	0.10 (± 0.36)	0.10 (± 0.30)	0.26 (± 0.53)	0.02 (± 0.14)	0.22 (± 0.42)	0.00 (± 0.00)	–	0.02 (± 0.14)	1.00 (± 0.00)	1.96 4.01%
Md	0.10 (± 0.30)	0.10 (± 0.30)	0.14 (± 0.40)	0.28 (± 0.64)	0.04 (± 0.20)	0.00 (± 0.00)	0.02 (± 0.14)	0.00 (± 0.00)	0.00 (± 0.00)	–	0.02 (± 0.14)	0.70 1.43%
V	0.06 (± 0.24)	0.14 (± 0.35)	0.04 (± 0.20)	0.12 (± 0.44)	0.06 (± 0.24)	0.02 (± 0.14)	0.02 (± 0.14)	0.04 (± 0.28)	0.04 (± 0.20)	0.00 (± 0.00)	–	0.54 1.10%
Sum	7.20 14.73%	9.26 18.94%	8.02 16.41%	5.24 10.72%	7.88 16.12%	1.70 3.48%	2.82 5.77%	1.60 3.27%	1.94 3.97%	1.60 3.27%	1.62 3.31%	48.88 100.00%

Most of the simulated intra-island cladogenetic events (i.e. parameter *y* in BioGeoBEARS) occurred on Tenerife. On average, 14.82 (± 2.64), i.e. 59.9 %, of the 24.76 recorded intra-island speciation events took place on Tenerife (Supplementary Data Table S2). In all 50 simulations of the BSM analysis, the five speciation events that gave rise to the Tenerifean subclade within sect. *Leuconium* (i.e. *A. haworthii* through *A. ciliatum* in Fig. 6) were inferred to have taken place on Tenerife (Supplementary Data File S4). On La Gomera, on average 2.88 intra-island speciation events per simulation occurred (Supplementary Data Table S2). La Palma, Gran Canaria and Madeira hosted approximately one intra-island speciation event each, and another single diversification event within East Africa was inferred in 48/50 BSMs for the split between *A. leucoblepharum* and the last common ancestor of *A. stuessyi* and *A. gorgoneum* (Supplementary Data File S4, node 37).

### Morphological and ecological divergence between sister lineages derived from intra- and inter-island speciation events

**Fig. 8.**
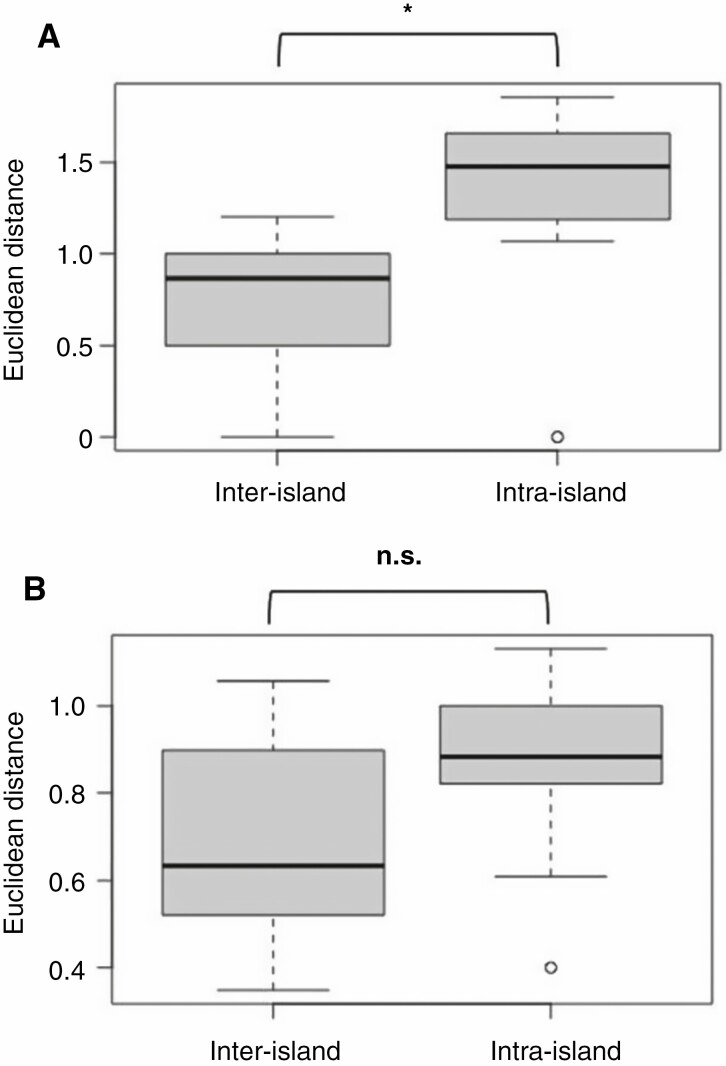
Boxplots of REDs in (A) morphological characters and (B) ecological habitat characteristics between well-supported sister lineages (see Materials and methods section for details concerning selection of sister lineages). REDs are separated by the biogeographical correlate (inter- or intra-island) of the diversification event giving rise to the respective sister lineages. Raw data contributing to these boxplots are listed in Table 6. *Difference between means is significant (*P* < 0.05); n.s., difference between means is not significant (*P* > 0.05).

We identified 17 nodes (listed in Table 6) that were consistently recovered in all phylogenetic analyses and received high support in at least one of these analyses. Four additional nodes in the Tenerifean subclade of sect. *Leuconium* (nodes 46–49 in Fig. 4) were inconsistently recovered across the phylogenetic analyses but were always associated with intra-island diversification on Tenerife (Table 6) (Supplementary Data File S4). Twelve of these altogether 21 nodes corresponded to intra-island diversification events and the remaining nine nodes to inter-island diversification events. The morphological REDs ranged between 0 and 1.86 among the intra-island diversification events (1.97 for node 47 of the concatML analyses; Table 6) and between 0 and 1.20 among the inter-island diversification events. The ecological REDs ranged between 0.4 and 1.13 (1.35 for node 48 of the concatML and ASTRAL analyses; Table 6) among intra-island and between 0.35 and 1.06 among inter-island diversification events. The morphological and ecological REDs corresponding to the four inconsistently recovered nodes 46–49 were very similar for the three principal phylogenetic analyses, i.e. concatML, ASTRAL and dating analysis (Table 6). For the comparison of morphological and ecological REDs between intra- and inter-island speciation events, we therefore used the respective mean values calculated across the three phylogenetic analyses (bottom row in Table 6). The difference in morphological REDs between intra- (1.33 ± 0.56) and inter-island diversification events (0.74 ± 0.39) was significant (two-sided *t*-test, *P* < 0.05), but the difference in ecological REDs (0.86 ± 0.23 and 0.66 ± 0.25, respectively) was not (*P* = 0.10; Fig. 8). On average, sister lineages were more divergent in ombrotype than in thermotype or elevation (Supplementary Data Table S3), and ombrotype was more divergent between intra- (0.55 ± 0.20) than between inter-island diversification events (0.39 ± 0.19), but not significantly (*P* = 0.09).

**Table 6:** Morphological and ecological REDs between sister lineages corresponding to those nodes that were recovered in all phylogenetic analyses and received high support in at least one of these analyses. For each node, the biogeographical correlate of diversification (i.e. inter- or intra-island diversification) as reconstructed in the BSM analysis is given, along with the islands on which diversification took place and the percentage of BSMs supporting this scenario. Numbers in brackets following morphological REDs refer to the numbers of morphological characters (of 10 characters observed, i.e. characters k–t in Fig. 3) in which the two sister lineages differ from each other. In addition to the morphological and ecological REDs, the distances in terms of growth form and the three habitat characteristics examined (i.e. elevation, thermo- and ombrotype) are given individually, with 0 corresponding to full identity and 1 corresponding to full disparity

Node number^a^	Sister lineage 1	Sister lineage 2	Biogeographical correlate of diversification	Morphological RED	Ecological RED
9	*A. diplocyclum*	Remainder of sect. *Greenovia*	94 % inter-island (92 % Tenerife—La Gomera)	1.07 (1/10)	0.38
10	*A. aizoon*	*A. aureum* + *A. dodrantale*	100 % intra-island (100 % Tenerife)	1.07 (1/10)	0.88
11	*A. aureum*	*A. dodrantale*	90 % intra-island (90 % Tenerife)	1.30 (1/10)	1.13
13	*A. goochiae*	*A. lindleyi*	100 % inter-island (96 % Tenerife—La Palma)	1.20 (1/10)	0.67
15	*A. simsii*	Remainder of clade Arboreum I	82 % intra-island (82 % Gran Canaria)	1.66 (4/10)	0.85
16	*A. undulatum*	*A. balsamiferum*, *A. korneliuslemsii* + *A. arboreum* s.l.	100 % inter-island (ambiguous)	0.87 (2/10)	0.65
17	*A. balsamiferum*	*A. korneliuslemsii* + *A. arboreum* s.l.	100 % inter-island (ambiguous)	0.81 (2/10)	1.06
20	*A. arboreum* subsp. *holochrysum* var. *rubrolineatum*	*A. arboreum* subsp. *arboreum*	100 % inter-island (100 % Gran Canaria—La Gomera)	1.00 (1/10)	0.92
22	*A. glandulosum* + *A. glutinosum*	Sect. *Canariensia*	100 % inter-island (98 % Tenerife—Madeira)	0.87 (2/10)	0.90
23	*A. glandulosum*	*A. glutinosum*	100 % intra-island (100 % Madeira)	1.80 (3/10)	1.00
24	*A. cuneatum*	Remainder of sect. *Canariensia*	98 % intra-island (98 % Tenerife)	1.86 (3/10)	1.08
25	*A. tabuliforme* + *A. canariense* subsp. *latifolium*	Remainder of *A. canariense*	84 % intra-island (84 % Tenerife)	0.00 (0/10)	0.40
26	*A. tabuliforme*	*A. canariense* subsp. *latifolium*	100 % inter-island (100 % Tenerife—La Gomera)	0.50 (0/10)	0.54
27	*A. canariense* subsp. *virgineum*	*A. canariense* subsp. *canariense* + *A. canariense* subsp. *christii*	98 % inter-island (92 % Tenerife—Gran Canaria)	0.33 (0/10)	0.52
28	*A. canariense* subsp. *canariense*	*A. canariense* subsp. *christii*	100 % inter-island (ambiguous)	0.00 (0/10)	0.35
33	*A. castello-paivae*	*A. decorum*	94 % intra-island (94 % La Gomera)	1.58 (3/10)	0.82
45	*A. haworthii*	Remainder of the Tenerifean Leuconium subclade	100 % intra-island (100 % Tenerife)	1.19 (5/10)	0.61
46^b^	Incongruent	Incongruent	100 % intra-island (100 % Tenerife)	1.19 (5/10) 1.69 (4/10) 1.47 (5/10)	0.63 0.67 0.76
47^b^	Incongruent	Incongruent	100 % intra-island (100 % Tenerife)	1.97 (5/10) 1.41 (2/10) 1.17 (4/10)	0.91 0.79 0.73
48^b^	Incongruent	Incongruent	100 % intra-island (100 % Tenerife)	1.25 (3/10) 1.25 (3/10) 1.90 (4/10)	1.35 1.35 0.56
49^b^	Incongruent	Incongruent	100 % intra-island (100 % Tenerife)	1.50 (2/10) 1.50 (2/10) 1.44 (2/10)	0.99 0.99 1.22
Mean of Tenerifean *Leuconium* subclade^c^	–	–	100 % intra-island (100 % Tenerife)	1.48 (3.42/10)	0.91

^a^Node numbers as specified in Fig. 4.

^b^These nodes in the Tenerifean subclade of sect. *Leuconium* were incongruent among the three principal phylogenetic analyses, i.e. concatML, ASTRAL and the dating analysis. Therefore, three REDs are presented for each node, from top to bottom in the same order as just listed. For comparison of the consequences of intra- and inter-island speciation events, the REDs corresponding to these nodes were replaced by the mean values given in the bottom row of this table.

^c^These values are REDs averaged across the nodes of the Tenerifean subclade of sect. *Leuconium* above node 45 (i.e. the inconsistently resolved nodes 46–49) as recovered in all our phylogenetic analyses. See Materials and methods section for more detailed information.

## DISCUSSION

### Biogeography and age of diversification

The present dating analysis based on ddRAD sequences inferred an age of 7.76 (± 4.64) myr for the split between *Aeonium* and *Monanthes*. This estimate is intermediate between the estimate inferred by Kim *et al.* (2008; 11.08 myr) and the estimate for the stem age of the clade comprising *Aeonium* + *Monanthes* inferred by Kondraskov *et al.* (2015; 6.73 myr). Although Tenerife was inferred as the ancestral area of *Aeonium* by our AAR, another ancestral area might be inferred if knowledge of the distribution of extinct *Aeonium* taxa were available. The observation that islands were colonized by *Aeonium* very shortly after their emergence (see Results section for La Palma and El Hierro) may suggest, as an alternative hypothesis, that colonization of Tenerife after its emergence may have occurred from the older eastern islands with subsequent extinction there.

The model we used for biogeographical analyses based on its highest likelihood and AICc_wt scores (Table 3), BAYAREALIKE+J, takes into account founder-event speciation or jump dispersal (parameter *j* in BioGeoBEARS). The concerns raised by Ree and Sanmartín (2018) about the biased preferability of parameter *j* in statistical selection of biogeographical models have recently been dispelled (Klaus and Matzke, 2020; Matzke, 2021), and founder-event speciation seems especially plausible in the context of island systems (Albaladejo *et al.*, 2021; Cala-Riquelme *et al.*, 2022). All currently accepted sections of *Aeonium* that occur on the Canary Islands probably originated before the emergence of El Hierro (1.1 myr ago) and La Palma (1.7 myr ago). Inter-island speciation by dispersal between Tenerife and either La Gomera to the west, Gran Canaria to the east or Madeira to the north, as well as intra-island cladogenesis within Tenerife, was inferred to play the major part in the emergence of the sections of *Aeonium* (Supplementary Data File S4), i.e. in the backbone of the phylogeny. When cladogenetic events within Tenerife are interpreted as sympatric speciation in this time period, it needs to be considered that this island most probably originated from three palaeo-islands (i.e. Adeje, Anaga and Teno) that were separated from each other by sea straits between ~8 and 3.5 myr ago (Caujapé-Castells *et al.*, 2017). Therefore, diversification that was reconstructed to have taken place in this timespan on Tenerife could be the result of founder-event speciation between palaeo-islands instead of intra-island speciation. The MRCA of *A. percarneum*, *A. lancerottense* and clade Arboreum II, which existed ~1.1 myr ago (95 % HPD = 0.37–2.01 myr ago; Table 2), was the oldest ancestral taxon, with a probability >8 % (namely 24 %) of colonizing the eastern Canary Islands. This pattern of a late colonization of Fuerteventura and/or Lanzarote has also been found in many other endemic lineages of the Canarian flora, such as the closely related *Aichryson* (Hühn *et al.*, 2022).

Back-colonization of the African continent took place twice independently in the evolution of *Aeonium*. The age estimates inferred here for both back-colonization events are younger than the estimates by Kim *et al.* (2008; 1.28 ± 0.21 and 2.95 ± 1.32 myr), but the topology of their *Aeonium* phylogeny differed strongly from ours. Back-colonization of the African continent after diversification on the Canary Islands has also been suggested for, among several others, *Matthiola* (Brassicaceae; Jaén-Molina *et al.*, 2009) and *Lotus* (Fabaceae; Jaén-Molina *et al.*, 2021). All phylogenetic analyses supported a sister relationship of the Cape Verde species *A. gorgoneum* to either *A. stuessyi* alone or to *A. stuessyi* plus the second East African species, *A. leucoblepharum*. Probably because the dated phylogeny that was used for the AAR analysis supported the former relationship, and because *A. gorgoneum* was thus nested within the East African clade, migration from (East) Africa to the Cape Verde Islands was reconstructed. A long-distance dispersal event between these two very distant regions (distance ~7500 km) would be one possible explanation for the disjunct distribution and was put forward by Thiv *et al.* (2010*b*), who argued that the numerous small seeds produced by *Aeonium* species may have facilitated long-distance dispersal. Vicariance of initially widespread lineages across northern Africa that subsequently went extinct in the central part of the distribution range due to aridification and emergence of the Sahara, ~6–7 myr ago (Senut *et al.*, 2009), has been discussed as the Rand Flora pattern (Christ, 1910; Pokorny *et al.*, 2015). After its first emergence, the Sahara area went through numerous periods of increased humidity, the latest dated to only 6000 years ago (Schuster *et al.*, 2006), and could therefore possibly have been inhabited by the MRCA of clade Arboreum II before a later period of aridification.

Overall, our results indicate a balanced proportion of cladogenetic events associated with either dispersal or sympatry (Table 4), suggesting equal numbers of inter- and intra-island diversification events. Although intra-island diversification certainly is not always the result of divergent selection (Gillespie *et al.*, 2020), our results suggest a balanced role of ecological and geographical drivers of diversification when compared with, e.g. *Argyranthemum*, where probably >70 % of diversification events had occurred within single islands (White *et al.*, 2020).

### Morphological and ecological divergence of sister lineages

We expected stronger ecological divergence resulting from intra-island, i.e. potentially sympatric, diversification than from inter-island diversification. Indeed, our comparison of ecological REDs between sister lineages derived from intra- and inter-island diversification events (henceforth referred to as intra- and inter-island sister lineages, respectively) indicates slightly higher ecological divergence in intra- versus inter-island diversification, but this difference was not significant (Fig. 8). This absence of a significant difference in ecological REDs may be due to the coarseness of the habitat-related information used in this study, namely elevation and categorical data for thermo- and ombrotype, where thermo- and ombrotype, overlapping for many taxa, may underestimate differences in microhabitat. However, vegetation types or biomes were not used instead of thermo- and ombrotype due to the lack of a thorough assessment of habitats for all *Aeonium* species. In contrast to the ecological REDs, morphological REDs between intra-island sister lineages were significantly higher than those between inter-island sister lineages (Fig. 8). Additionally, intra-island sister lineages were more frequently differentiated from each other in terms of growth form (mean RED ± standard deviation: 0.35 ± 0.44) when compared with inter-island sister lineages (Supplementary Data Table S4, 0.06 ± 0.17). Growth form, in turn, has been discussed as a potential indicator of the occupied ecological niche of plants (e.g. Lowrey, 1995), and especially of *Aeonium* (Lösch, 1990; Mes and ’t Hart, 1996; Jorgensen and Olesen, 2001; Mort *et al.*, 2007; P. dos Santos, unpubl. res.).

In summary, we found clear signs of stronger divergence in morphology among intra- than among inter-island sister lineages, and this may be the result of ecologically divergent selection in intra-island diversification events. To illustrate this further, patterns of diversification and morphological and ecological divergence of the Canariensia clade (*A. canariense chr.* through *A. glandulosum* in Fig. 3) may serve as an example. Although the initial diversification event in this clade (node 22), which involved the colonization of Madeira, was associated with relatively high morphological and ecological divergence (0.87 and 0.9, respectively; Table 6), the intra-island diversification between *A. glutinosum* and *A. glandulosum* (node 23) on Madeira was associated with even higher morphological (1.8; shift in growth form and leaf pubescence) and ecological divergence (1.0; shift in ombrotype). There were two more intra-island diversification events in this clade, namely the split between *A. cuneatum* and the remainder of sect. *Canariensia* (node 24) and the split between *A. tabuliforme* + *A. canariense* subsp. *latifolium* and the other subspecies of *A. canariense* (node 25). The former diversification event was associated with the highest morphological divergence recorded here (1.86; Table 6), because *A. cuneatum* differs from *A. canariense* s.l. (including *A. tabuliforme*) in having ciliate, glabrous and distinctly glaucous leaves and a slightly different petal colour. Finally, *A. canariense* s.l. is strikingly uniform in morphology and elevational range, and its (sub)species only differ slightly in thermo- and ombrotype (Fig. 3), resulting in low morphological and ecological REDs for the corresponding inter-island sister lineages (nodes 26–28 in Table 6).

### *Co-occurrence and hybridization among* Aeonium *species*

The observation that plant radiations in the Canary Islands, especially species-rich radiations, are mostly monophyletic, a pattern that is best explained by a single colonization event, has led to the theory that niche pre-emption by the earliest successful colonizers prevented the establishment of later colonizers closely related to a given radiation (Silvertown, 2004). Previous studies have clearly shown that the Macaronesian clade of tribe Aeonieae, as well as *Aeonium*, are monophyletic lineages (Mes, 1995; Mort *et al.*, 2002), implying that the Macaronesian Aeonieae, too, are the product of a single colonization event of these islands and subsequent diversification. However, considering the results of our AAR analysis for *Aeonium*, a more differentiated picture emerges with respect to the role of niche pre-emption in the evolution of *Aeonium*, because some of the islands were successfully colonized several times independently (Fig. 7) and hence after the arrival of other *Aeonium* species on the same islands.

For example, Gran Canaria was colonized at least six times independently by (1) the MRCA of clade Arboreum I (node 14 in Fig. 4), (2) *A. canariense* subsp. *virgineum* (node 27), (3) *A. percarneum*, (4) *A. arboreum* subsp. *arboreum* (node 20), (5) *A. aureum* (node 11), and (6) *A. spathulatum*. These six lineages are those that we could define as recurrent across the majority of the 50 BSMs. However, the BSM simulations identified additional migrations between islands/regions among the outgroup taxa and along ancestral branches, which are often later reversed (Supplementary Data File S4). This explains the lower number of colonization events given here in comparison with those in Table 5. La Gomera was reached independently by at least seven lineages, namely (1) *A. sedifolium*, (2) the widespread *A. diplocyclum* and *A. spathulatum* (either they reached La Gomera independently or the MRCA of sects *Chrysocome* and *Greenovia* was distributed on La Gomera), (3) *A. lindleyi* subsp. *viscatum*, (4) *A. arboreum* subsp. *holochrysum* var. *rubrolineatum*, (5) *A. canariense* subsp. *latifolium*, (6) *A. appendiculatum*, and (7) the clade of *A. gomerense*, *A. decorum* and *A. castello-paivae* (Fig. 7). The highest number of independent colonization events was inferred for La Palma (at least eight; see Fig. 7). El Hierro received at least six lineages of *Aeonium* independently (Fig. 7). Tenerife, which acted most frequently as the source area, was colonized secondarily only in two instances: (1) along the ancestral lineage of *A. arboreum* subsp. *holochrysum* var. *holochrysum* (nodes 16–19), a variety that also colonized El Hierro and La Palma, and (2) by *A. decorum* (Supplementary Data File S4). Thus, Tenerife only received 13 % of all dispersal events among the western Canary Islands. In addition, Tenerife also has the lowest rates of co-occurrence and hybrid formation among these islands with only 43 and 15 %, respectively, of all possible species pairings. Together with our finding that Tenerife and La Gomera have the oldest record of continuous habitation by *Aeonium*, followed by Gran Canaria, La Palma and El Hierro (see Results section), it seems that different taxa of *Aeonium* are more likely to co-occur and hybridize with one another on islands that were colonized by *Aeonium* more recently, but the number of independent colonization events does not seem to be relevant for the observed rates of co-occurrence and hybridization.

The only obvious outlier in this negative relationship between the duration of habitation and rates of co-occurrence and hybridization is Gran Canaria, where every taxon may co-occur with every other taxon, although in some cases with only little overlap in distribution area. The reason for this apparent lack of spatial separation of *Aeonium* taxa on Gran Canaria might be the strong influence of human activities, especially deforestation, on this island (Macías-Hernández, 1992; Morales *et al.*, 2009). This may have eliminated important dispersal barriers and brought different taxa into closer contact with each other. The time of colonization of Gran Canaria inferred by our AAR (3.55 myr ago; 95 % HPD = 1.34–6.3 myr ago) roughly coincides with the Roque Nublo period (~5.3–3.7 myr ago), a catastrophic volcanic event that is discussed as cause for massive extinction on Gran Canaria (Anderson *et al.*, 2009; González-Pérez and Caujapé-Castells, 2021). This may suggest that the relatively late colonization of Gran Canaria by *Aeonium* inferred here was preceded by earlier colonizations that went extinct due to the Roque Nublo events. Furthermore, as the youngest of the Canary Islands, El Hierro would be expected to have the highest rates of co-occurrence and hybrid formation (80 % co-occurrence and 40 % hybridization observed), but it is closely surpassed by La Palma (89 and 44 %, respectively), possibly because relatively few taxa of *Aeonium* have (yet) reached El Hierro. Tenerife, on the other hand, where the highest number of intra-island diversification events was reconstructed (see Results section), has the lowest rates of co-occurrence and hybrid formation among *Aeonium* taxa, indicating a high level of ecological differentiation and reproductive barriers between these taxa. Interestingly, the Hawaiian silversword alliance (Asteraceae) seems to be a similar case of unbalanced distribution of natural hybrids. There, the western islands of the archipelago, which were colonized earlier by silversword ancestors (Landis *et al.*, 2018), harbour lower proportions of naturally occurring hybrid taxa (relative to the number of possible combinations) than the central and eastern islands of the archipelago (Carr, 1985), which were colonized later. The overall high potential for the formation of fertile hybrids in *Aeonium* might also be the reason for the observed incongruences among our different phylogenies (Figs 2–4), but this was not investigated in depth here.

In conclusion, ecological differentiation and reproductive barriers among *Aeonium* taxa seem to be more pronounced on islands with a longer history of habitation, which is well in line with our second hypothesis. This pattern may result from competitive exclusion of closely related species (e.g. Davies *et al.*, 2007) but, to our knowledge, this concept has not yet been discussed for island radiations. The fact that essentially the same pattern was found for the distribution of hybrids among members of the Hawaiian silversword alliance underpins the relevance of the duration of shared island habitation for the establishment of reproductive barriers.

## SUPPLEMENTARY DATA

Supplementary data are available online at https://academic.oup.com/aob and consist of the following.

Table S1: plant material. Detailed information about specimens used in this study. Table S2: intra-island diversification events. Counts of intra-island diversification events across the 50 simulations of the BSM analysis. Table S3: ecological REDs. REDs in the individual habitat characteristics for those nodes that were recovered in all phylogenetic analyses and received high support in at least one of these analyses. Table S4: morphological REDs. REDs in the individual morphological characters for those nodes that were recovered in all phylogenetic analyses and received high support in at least one of these analyses. Figure S1: ISC assembly metrics. Box- and scatterplots of assembly metrics obtained from ipyrad over the course of in-sample clustering for different clustering thresholds. Figure S2: phylogeny including *A. lindleyi* subsp. *viscatum*. ML phylogeny of *Aeonium*, including *A. lindleyi* subsp. *viscatum*, inferred from a concatenated supermatrix of all assembled loci. Figure S3: Chronogram with 320–500/ASTRAL as starting tree. Chronogram of *Aeonium* inferred using BEAST, based on the sequence dataset of those 357 loci in the 320- to 500-nt length range that had sequence information for at least 20 different samples. File S1: MultiQC report R1. Multiple FastQC report summarizing sequence counts, sequence quality and other statistics for the forward reads from all samples included in our ddRADseq laboratory analysis. File S2: MultiQC report R2. Multiple FastQC report summarizing sequence counts, sequence quality and other statistics for the reverse reads from all samples included in our ddRADseq laboratory analysis. File S3: locus properties. Properties of all 4280 loci of our final assembly that was used for the all/RAxML and all/ASTRAL analyses. File S4: biogeographical stochastic maps. Fifty simulations of range shifts and migration across the dated *Aeonium* phylogeny obtained from our BSM analysis. File S5: BSC assembly metrics. Assembly metrics and corresponding diagrams for between-sample clustering using ipyrad with different clustering thresholds.

mcad033_suppl_Supplementary_Data_S1

mcad033_suppl_Supplementary_Data_S2

mcad033_suppl_Supplementary_Data_S3

mcad033_suppl_Supplementary_Data_S4

mcad033_suppl_Supplementary_Data_S5

mcad033_suppl_Supplementary_Figure_S1

mcad033_suppl_Supplementary_Figure_S2

mcad033_suppl_Supplementary_Figure_S3

mcad033_suppl_Supplementary_Table_S1

mcad033_suppl_Supplementary_Table_S2

mcad033_suppl_Supplementary_Table_S3

mcad033_suppl_Supplementary_Table_S4
