# Supplementary material for: Inter- and intra-island speciation and their morphological and ecological correlates in Aeonium (Crassulaceae), a species-rich Macaronesian radiation
Source: Ann Bot. 2023 Feb 23;131(4):697–721. doi: 10.1093/aob/mcad033 (PMC10147336; doi:10.1093/aob/mcad033)

**File S1: MultiQC report R1.** Multiple FastQC (MultiQC) report summarizing sequence counts, sequence quality and other statistics for the forward reads from all samples included in our ddRADseq laboratory analysis.


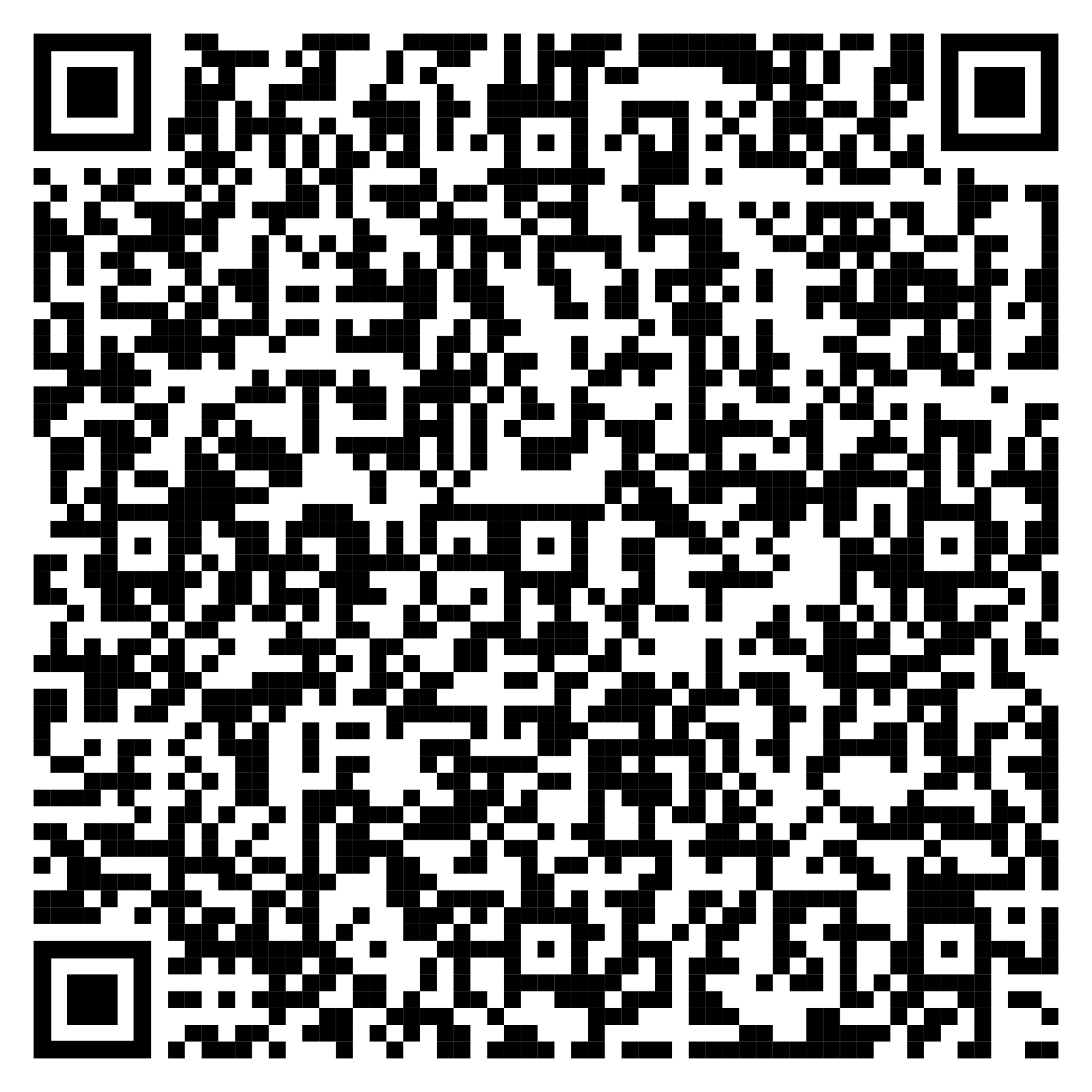

Supplement: mcad033_suppl_Supplementary_Data_S1 [file mcad033_suppl_supplementary_data_s1.docx]
