# Supplementary material for: Inter- and intra-island speciation and their morphological and ecological correlates in Aeonium (Crassulaceae), a species-rich Macaronesian radiation
Source: Ann Bot. 2023 Feb 23;131(4):697–721. doi: 10.1093/aob/mcad033 (PMC10147336; doi:10.1093/aob/mcad033)

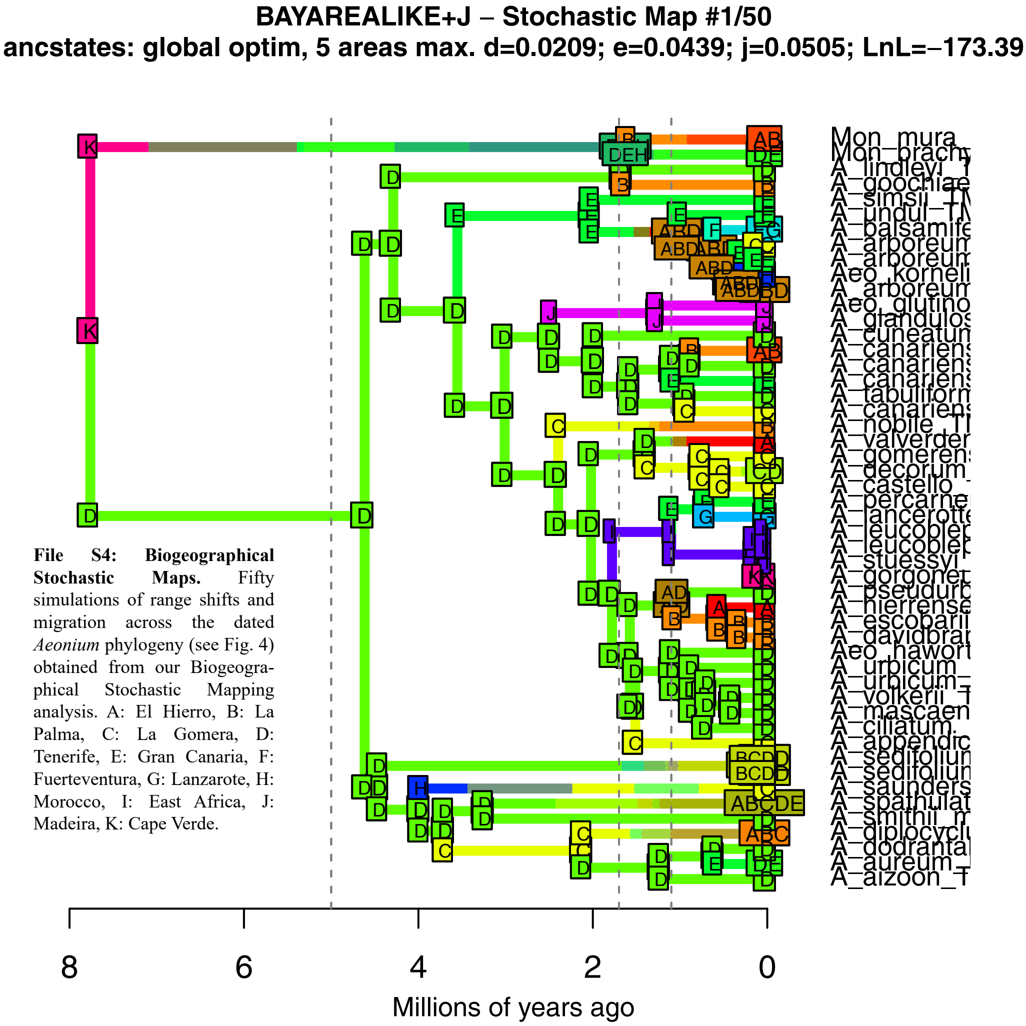

File S4: Biogeographical Stochastic Maps. Fifty simulations of range shifts and migration across the dated *Aeonium* phylogeny (see Fig. 4) obtained from our Biogeographical Stochastic Mapping analysis. A: El Hierro, B: La Palma, C: La Gomera, D: Tenerife, E: Gran Canaria, F: Fuerteventura, G: Lanzarote, H: Morocco, I: East Africa, J: Madeira, K: Cape Verde.

BAYAREALIKE+J − Stochastic Map #2/50
ancstates: global optim, 5 areas max. d=0.0209; e=0.0439; j=0.0505; LnL=−173.39

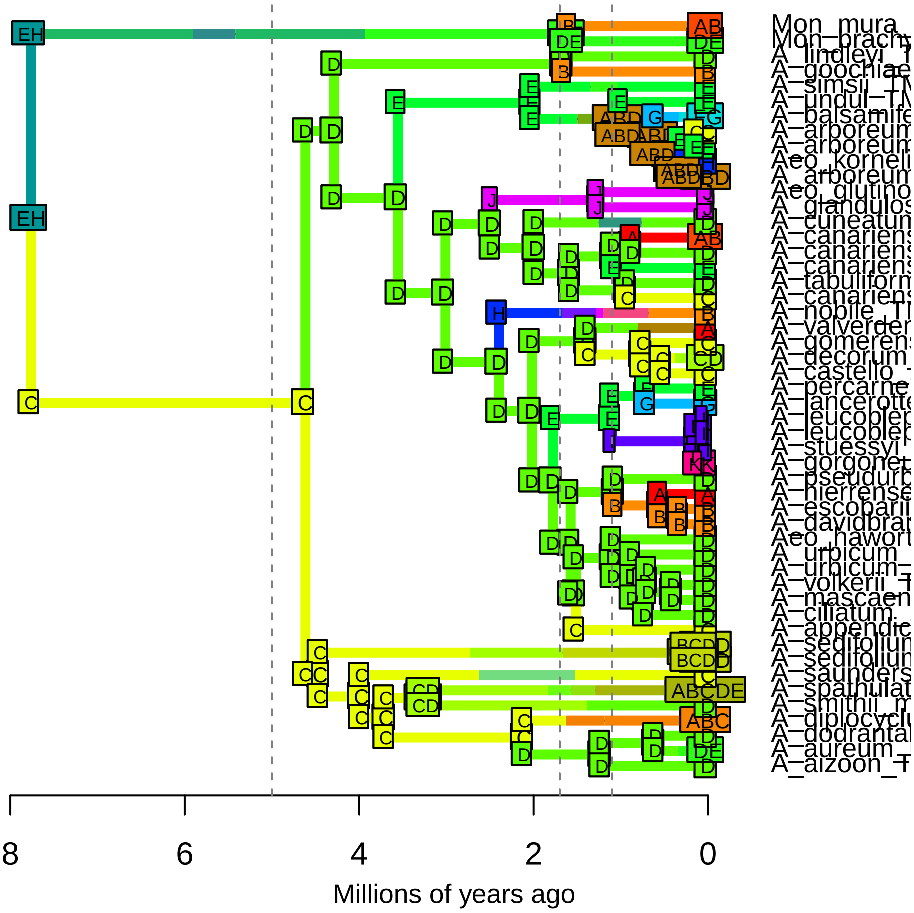

BAYAREALIKE+J − Stochastic Map #3/50
ancstates: global optim, 5 areas max. d=0.0209; e=0.0439; j=0.0505; LnL=−173.39

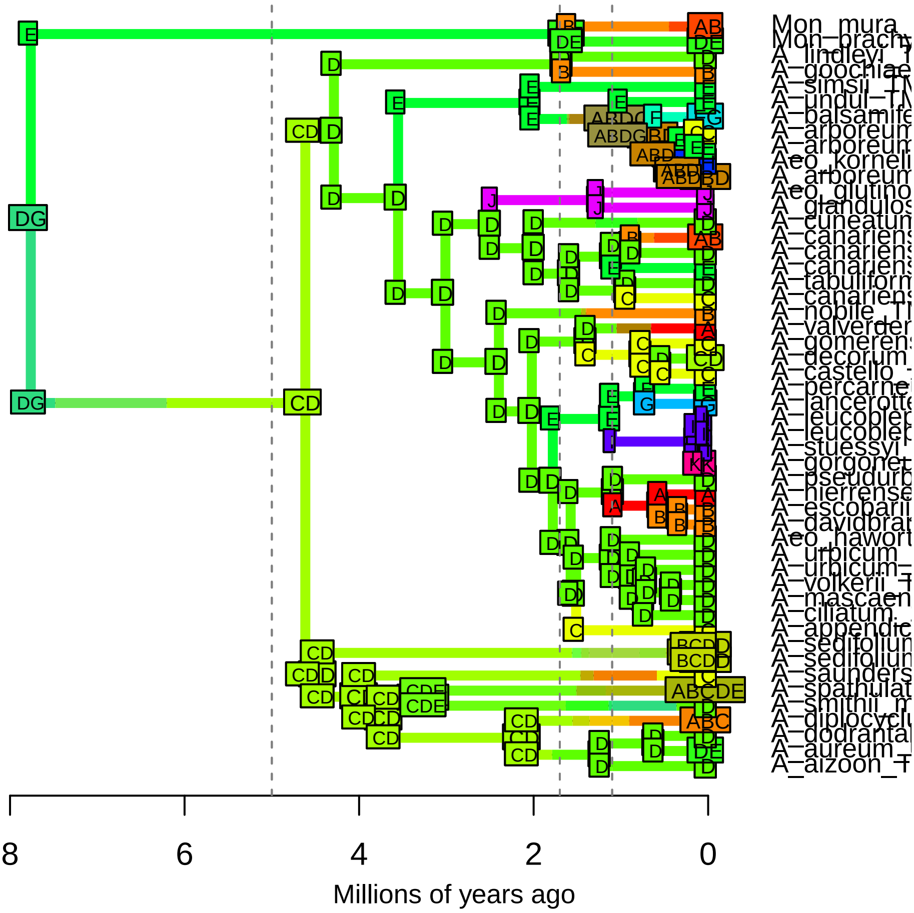

BAYAREALIKE+J − Stochastic Map #4/50

ancstates: global optim, 5 areas max. d=0.0209; e=0.0439; j=0.0505; LnL=−173.39

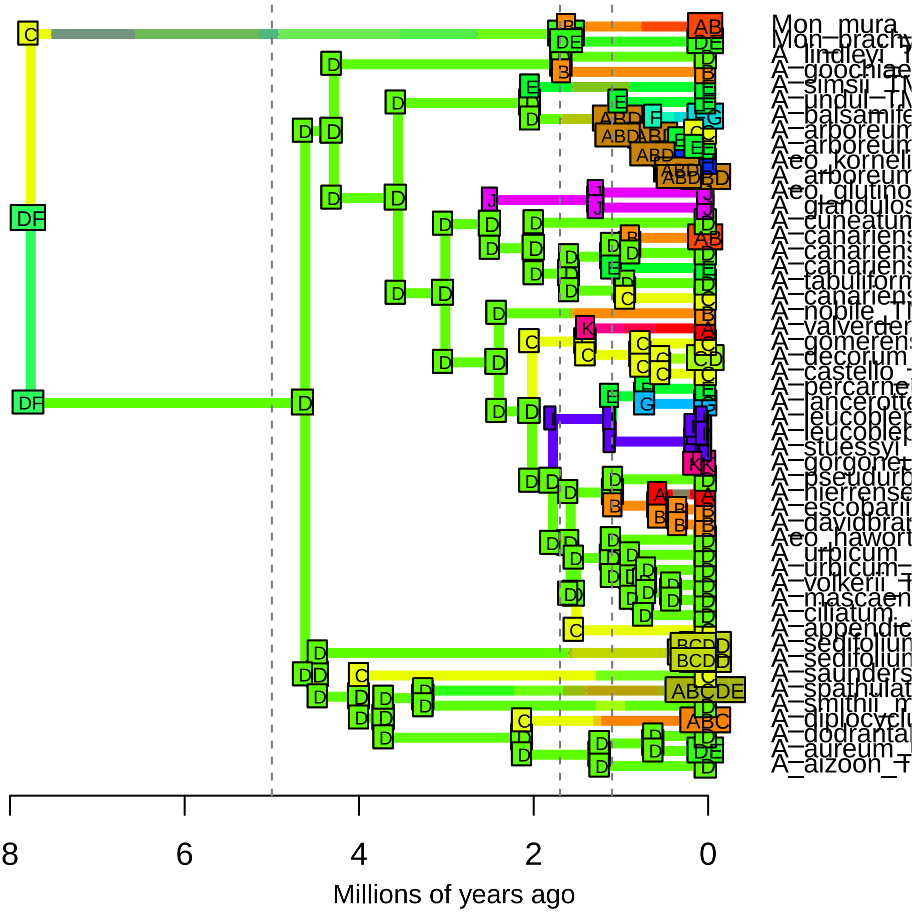

BAYAREALIKE+J − Stochastic Map #5/50
ancstates: global optim, 5 areas max. d=0.0209; e=0.0439; j=0.0505; LnL=−173.39

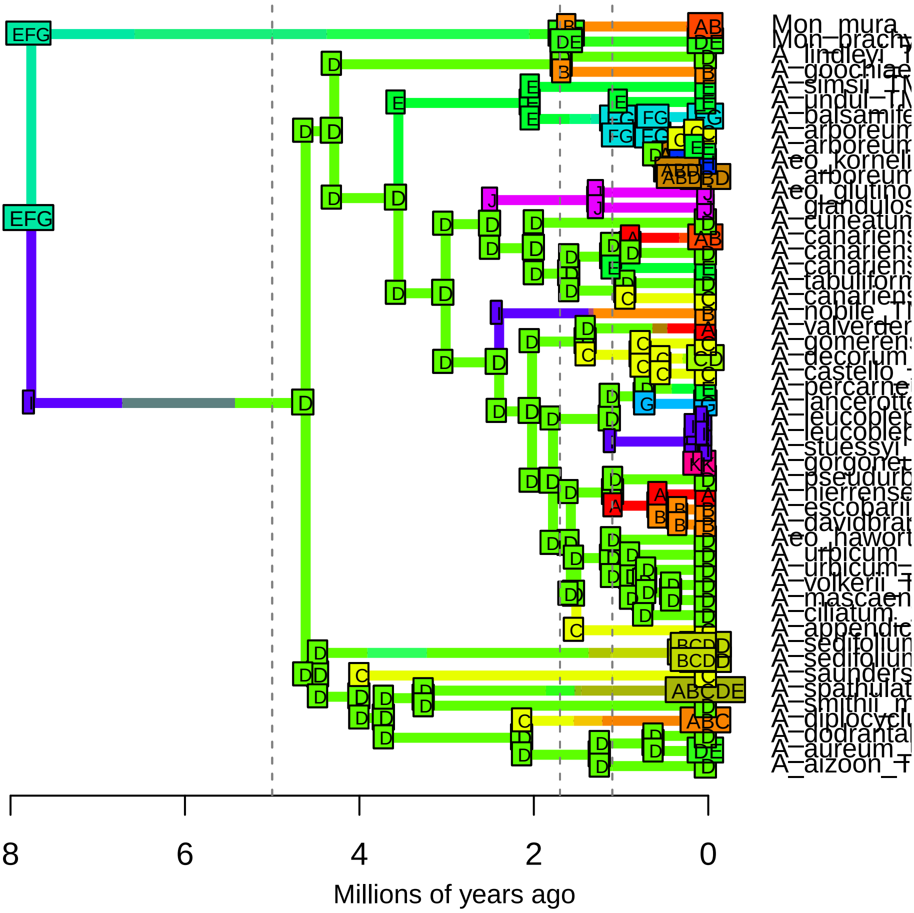

BAYAREALIKE+J − Stochastic Map #6/50
ancstates: global optim, 5 areas max. d=0.0209; e=0.0439; j=0.0505; LnL=−173.39

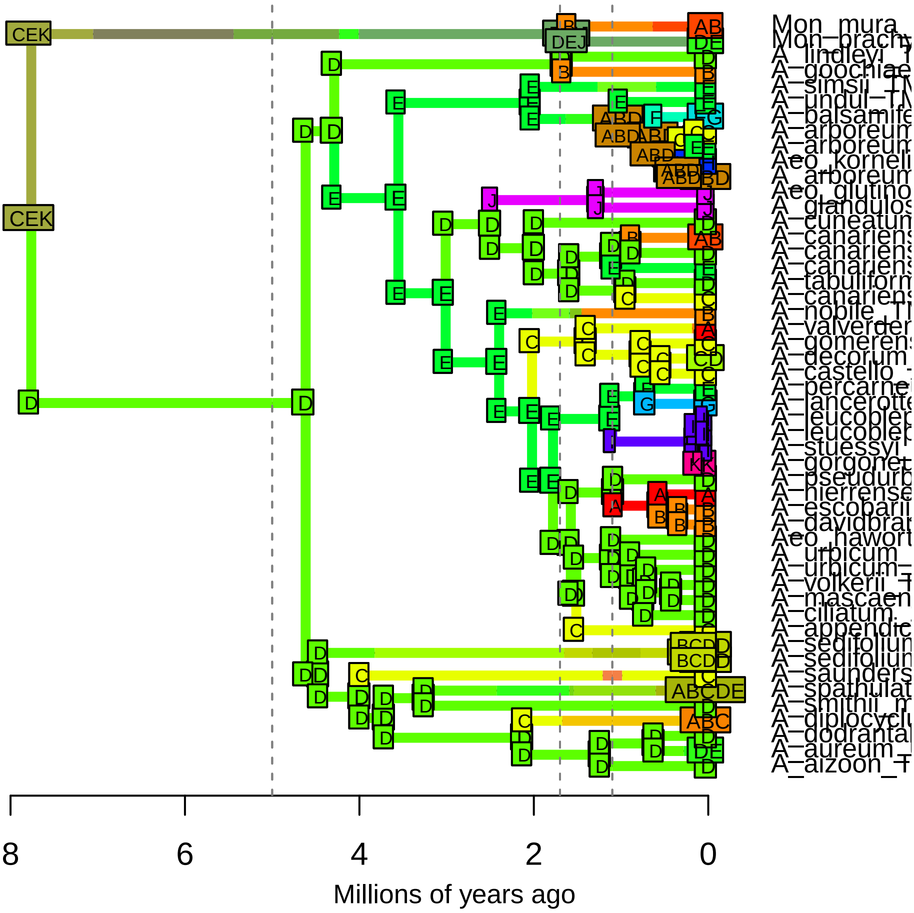

BAYAREALIKE+J − Stochastic Map #7/50
ancstates: global optim, 5 areas max. d=0.0209; e=0.0439; j=0.0505; LnL=−173.39

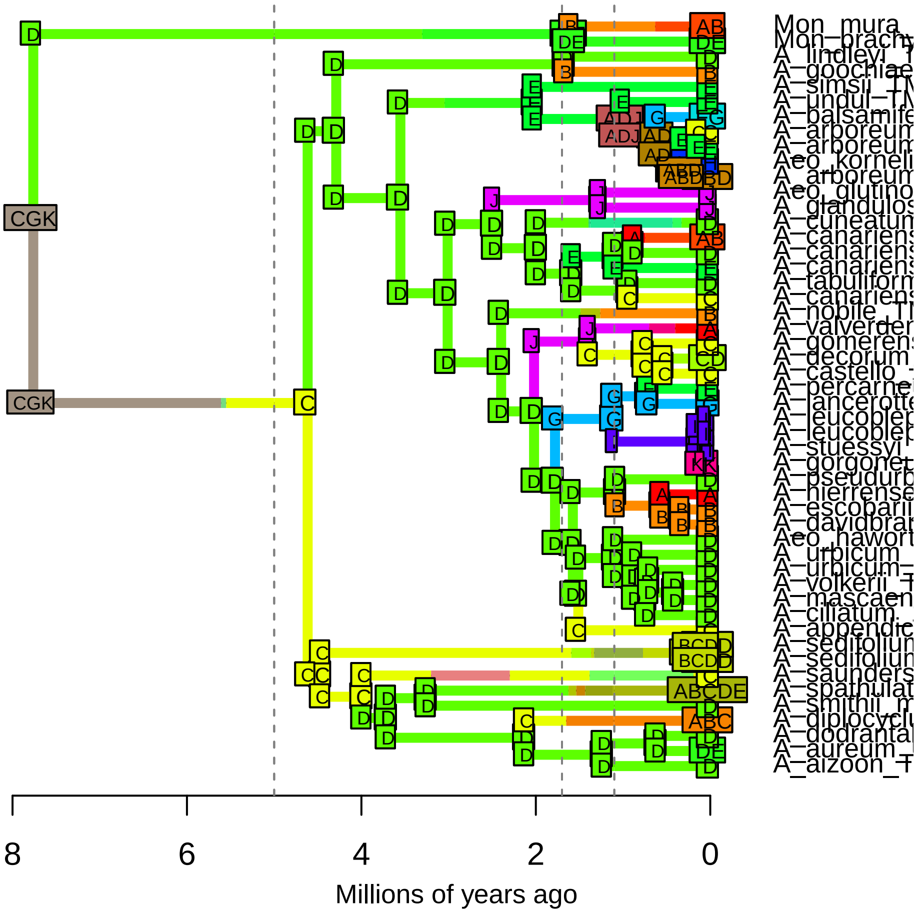

BAYAREALIKE+J − Stochastic Map #8/50
ancstates: global optim, 5 areas max. d=0.0209; e=0.0439; j=0.0505; LnL=−173.39

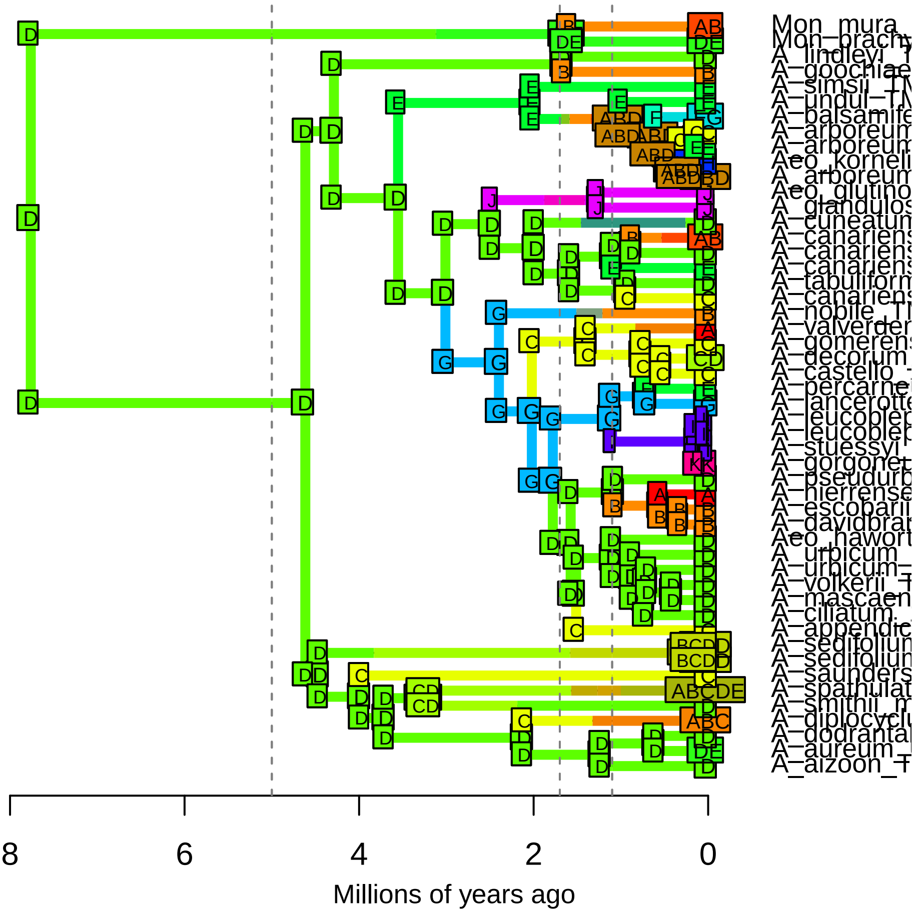

BAYAREALIKE+J − Stochastic Map #9/50

ancstates: global optim, 5 areas max. d=0.0209; e=0.0439; j=0.0505; LnL=−173.39

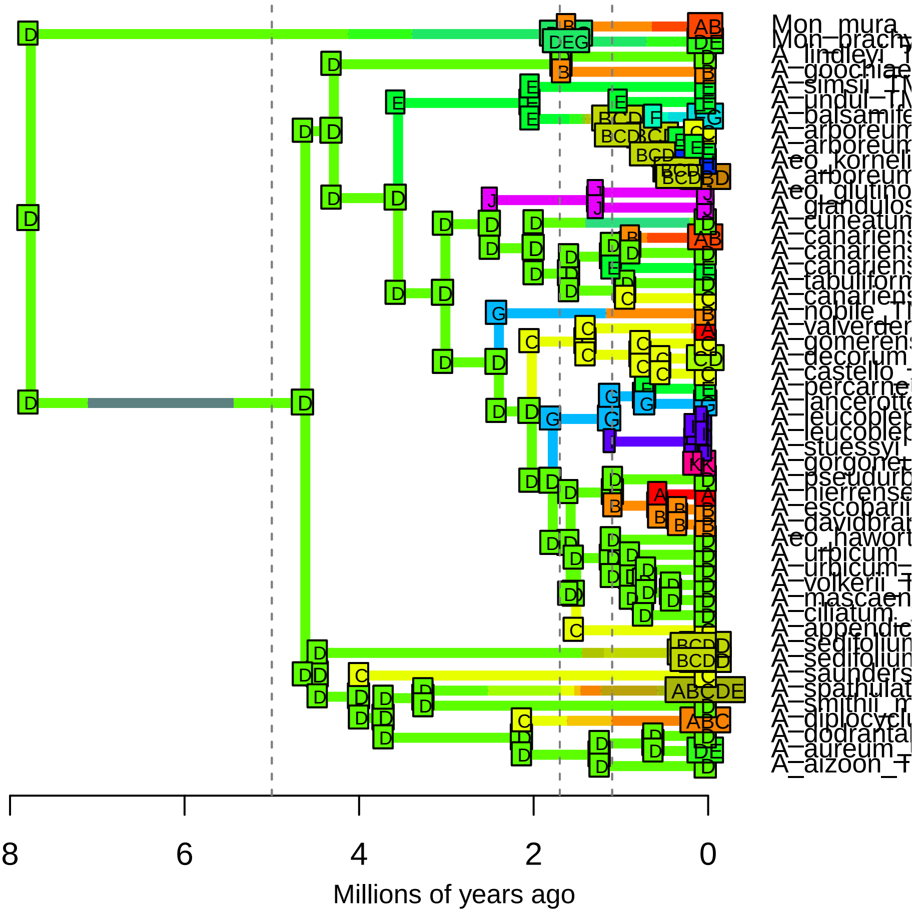

BAYAREALIKE+J − Stochastic Map #10/50

ancstates: global optim, 5 areas max. d=0.0209; e=0.0439; j=0.0505; LnL=−173.39

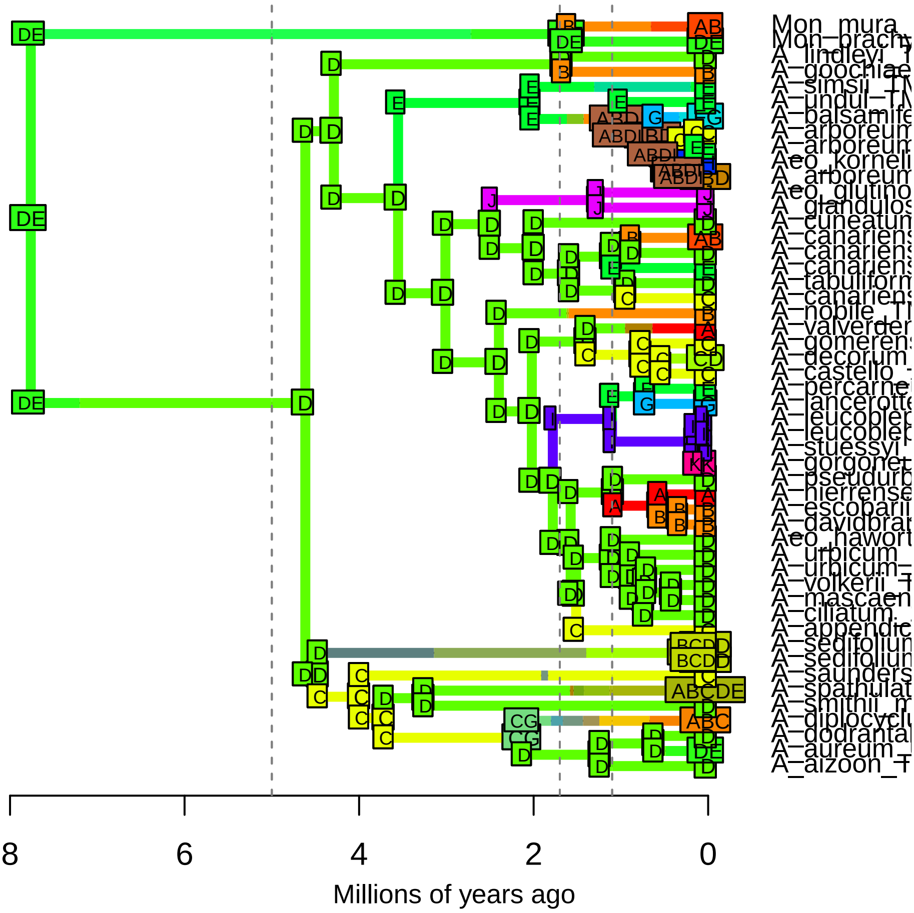

BAYAREALIKE+J − Stochastic Map #11/50
ancstates: global optim, 5 areas max. d=0.0209; e=0.0439; j=0.0505; LnL=−173.39

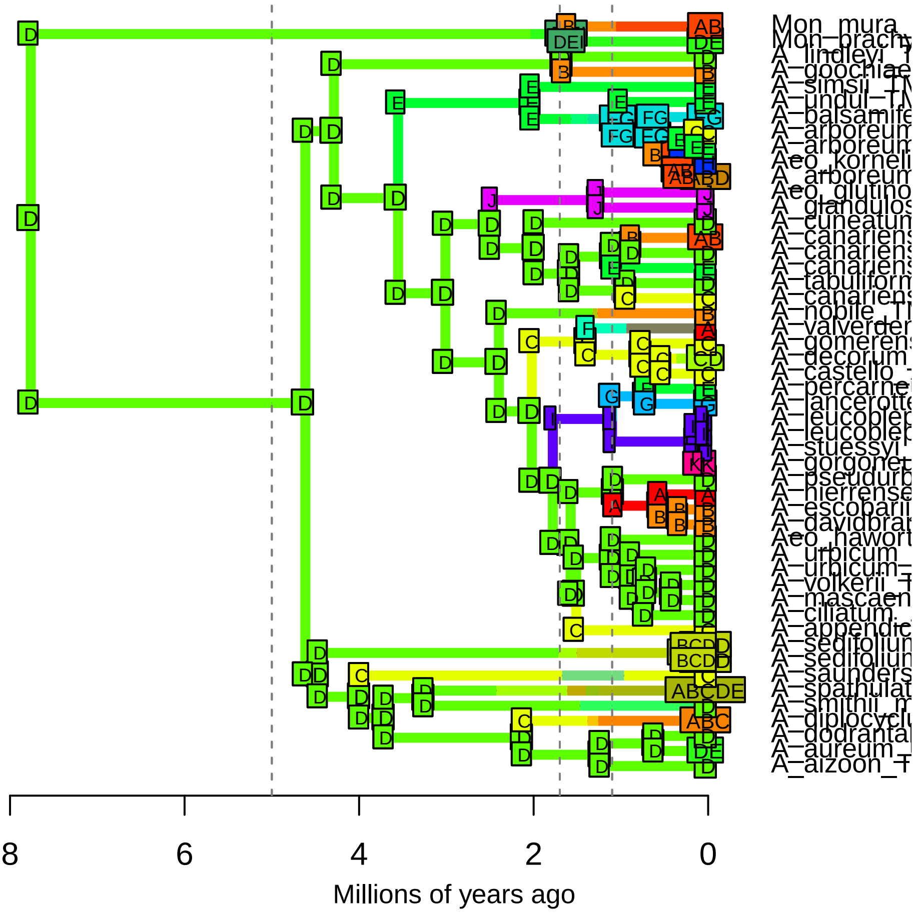

BAYAREALIKE+J − Stochastic Map #12/50
ancstates: global optim, 5 areas max. d=0.0209; e=0.0439; j=0.0505; LnL=−173.39

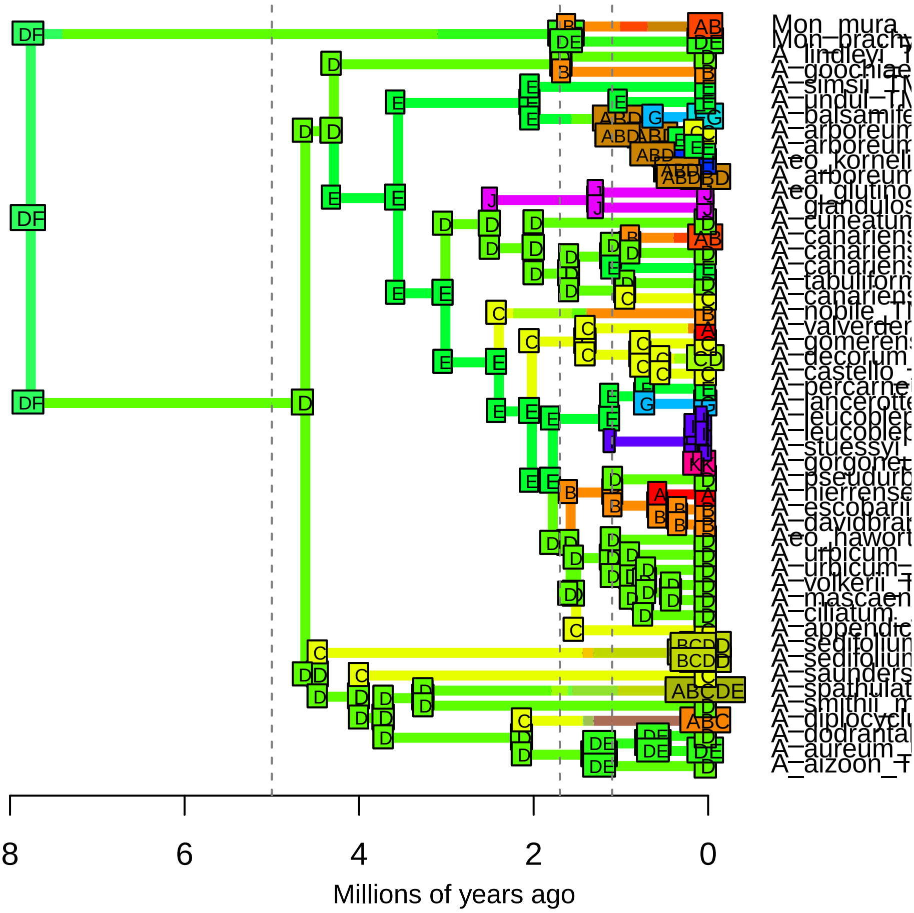

BAYAREALIKE+J − Stochastic Map #13/50
ancstates: global optim, 5 areas max. d=0.0209; e=0.0439; j=0.0505; LnL=−173.39

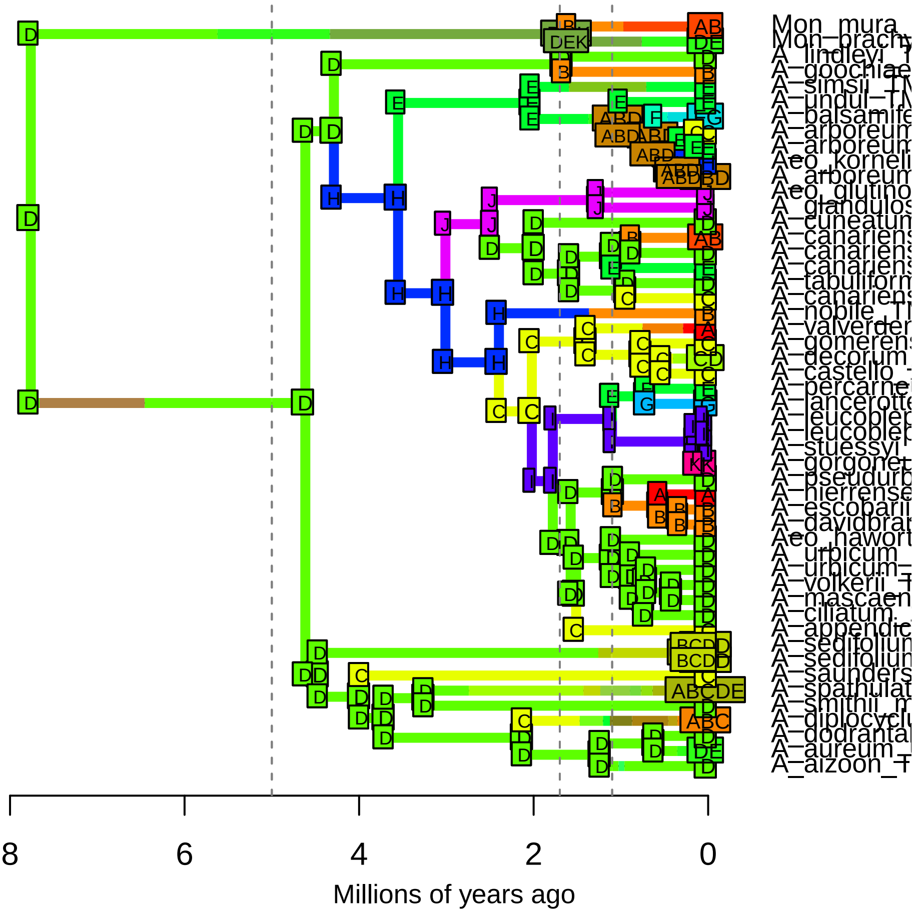

BAYAREALIKE+J − Stochastic Map #14/50
ancstates: global optim, 5 areas max. d=0.0209; e=0.0439; j=0.0505; LnL=−173.39

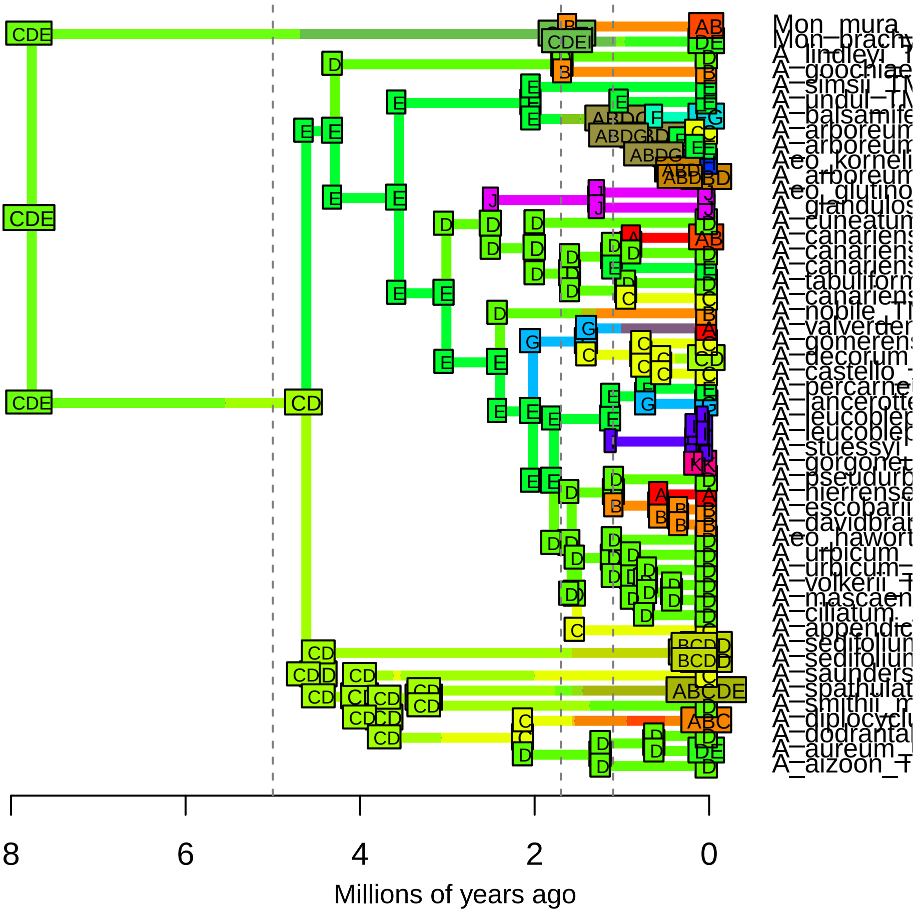

BAYAREALIKE+J − Stochastic Map #15/50
ancstates: global optim, 5 areas max. d=0.0209; e=0.0439; j=0.0505; LnL=−173.39

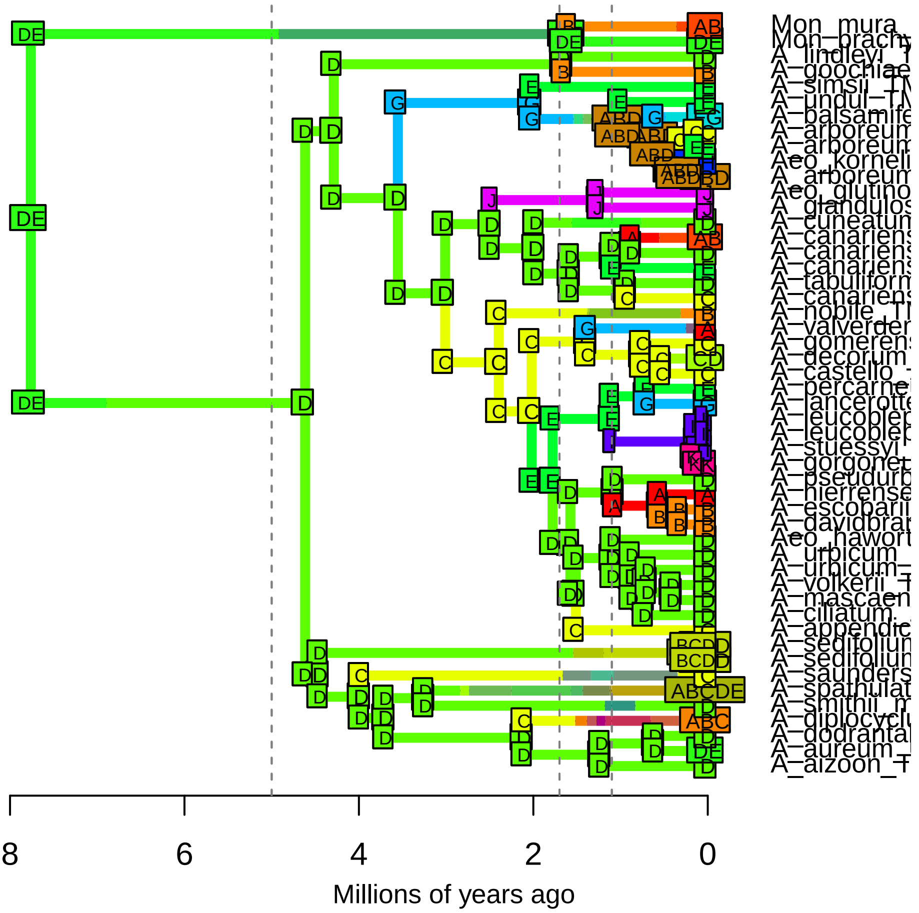

BAYAREALIKE+J − Stochastic Map #16/50

ancstates: global optim, 5 areas max. d=0.0209; e=0.0439; j=0.0505; LnL=−173.39

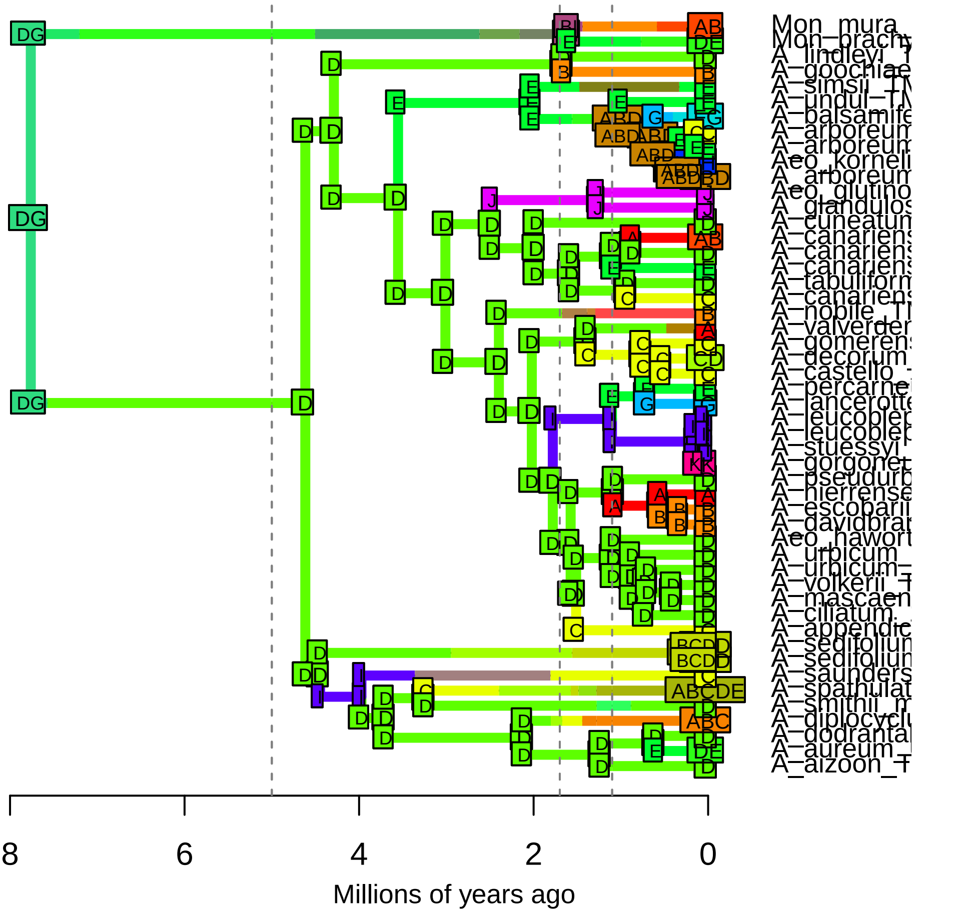

BAYAREALIKE+J − Stochastic Map #17/50
ancstates: global optim, 5 areas max. d=0.0209; e=0.0439; j=0.0505; LnL=−173.39

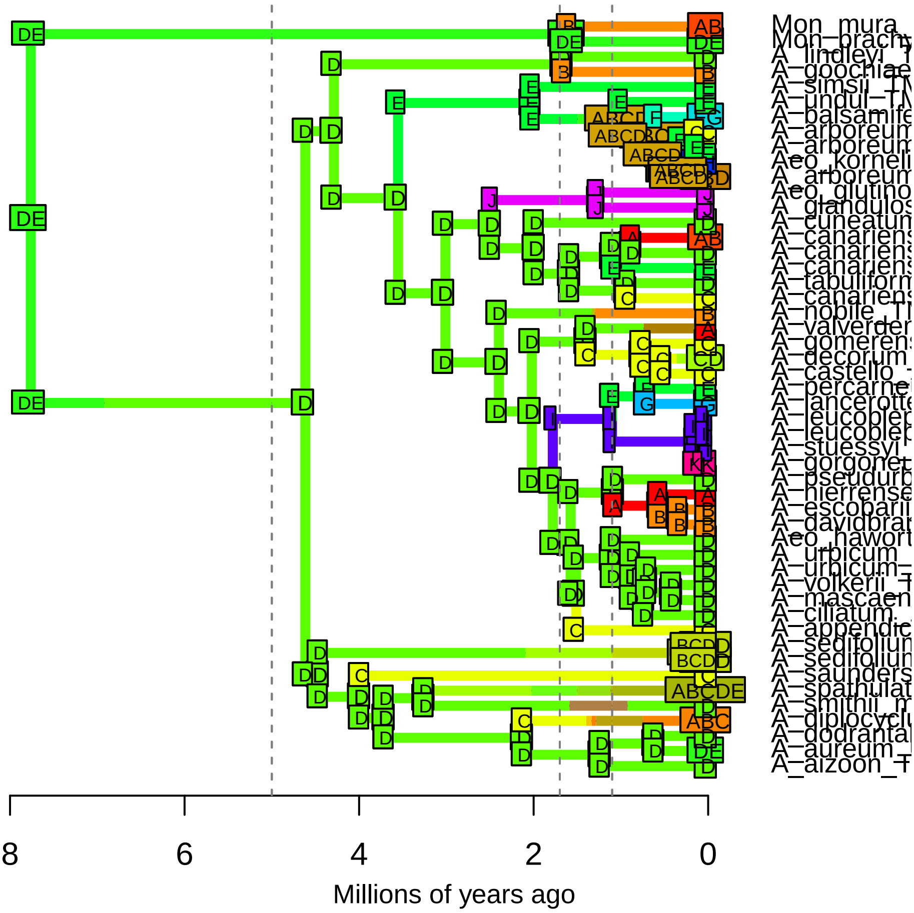

BAYAREALIKE+J − Stochastic Map #18/50
ancstates: global optim, 5 areas max. d=0.0209; e=0.0439; j=0.0505; LnL=−173.39

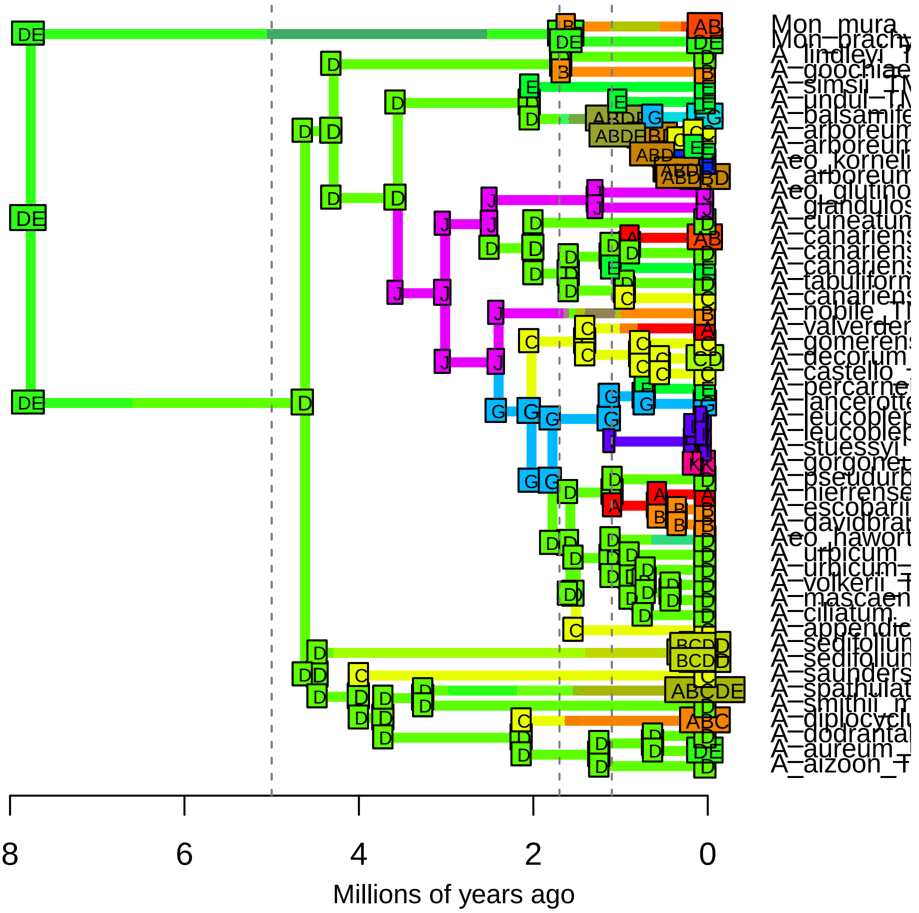

BAYAREALIKE+J − Stochastic Map #19/50
ancstates: global optim, 5 areas max. d=0.0209; e=0.0439; j=0.0505; LnL=−173.39

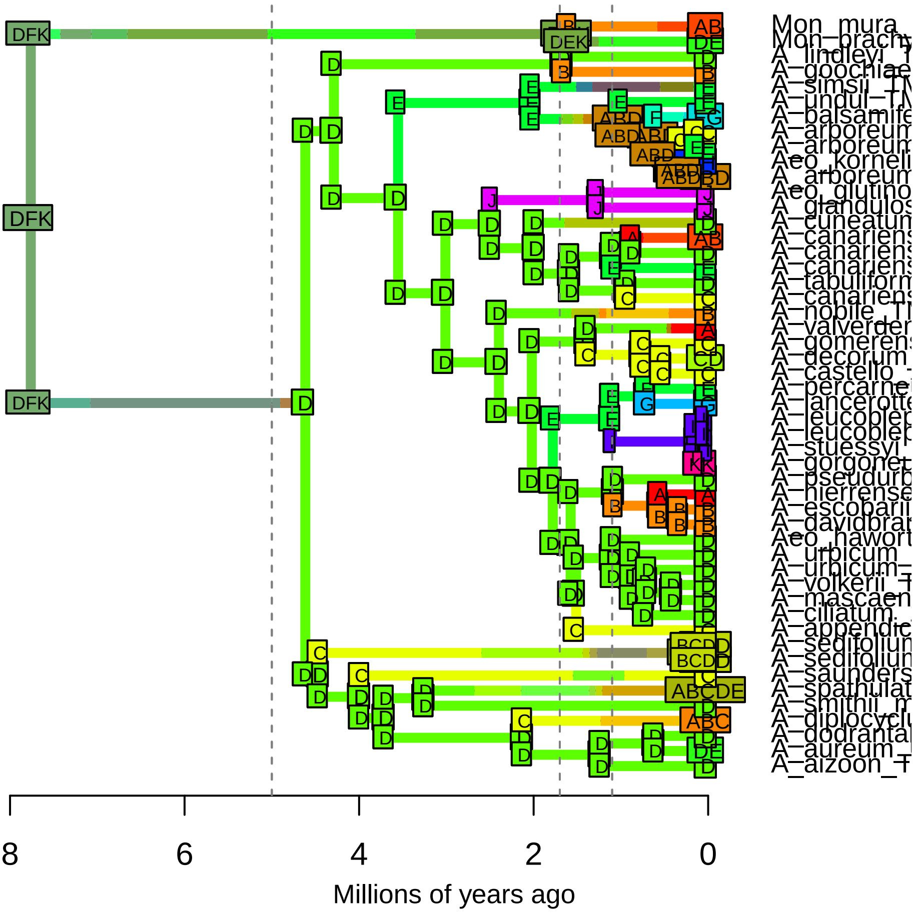

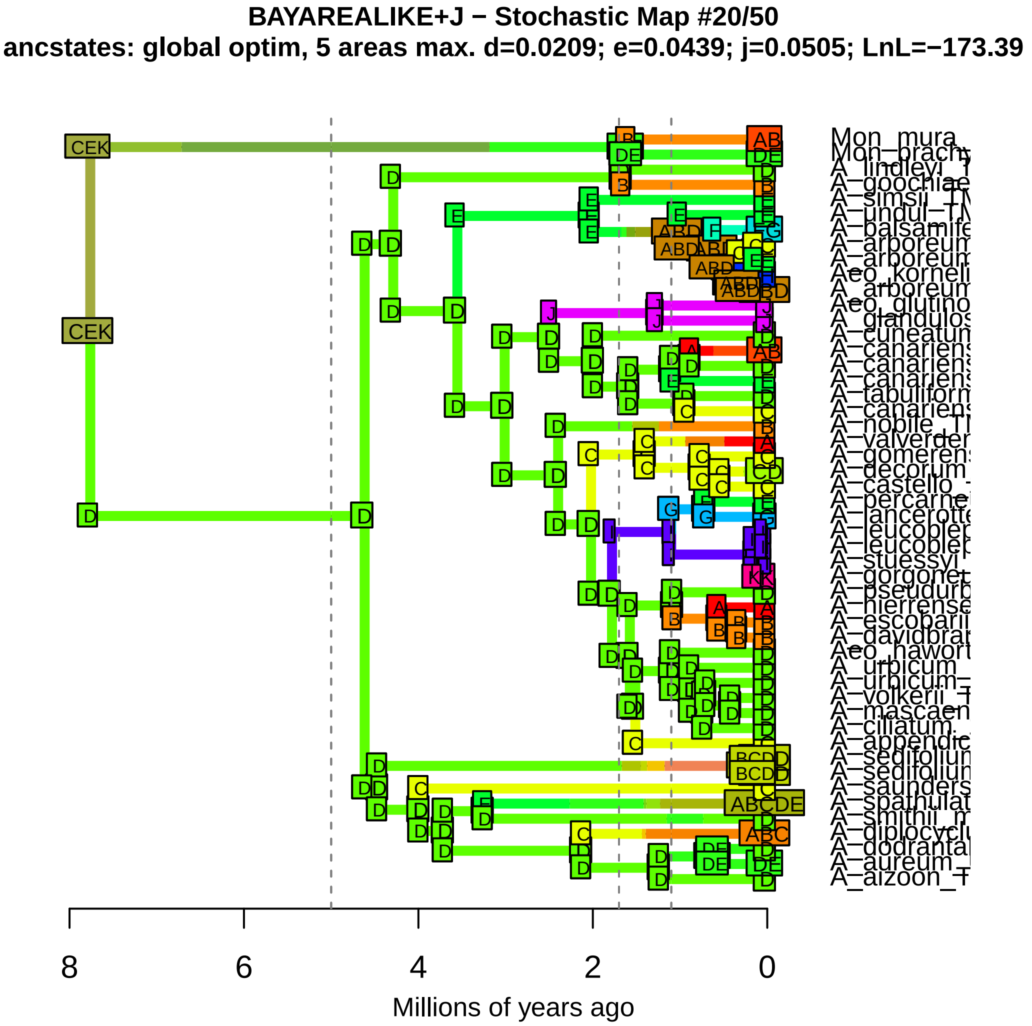
BAYAREALIKE+J − Stochastic Map #20/50
ancstates: global optim, 5 areas max. d=0.0209; e=0.0439; j=0.0505; LnL=−173.39
Mon_mura
Mon−brachy
A−lindleyi_T
A−goochiae
A−simsii_TN
A−undul−TN
A−balsamife
A−arboreum
A−arboreum
Aeo_korneli
A_arboreum
Aeo_glutino
A−glandulos
A−cuneatum
A−canariens
A−canariens
A−canariens
A−tabuliform
A−canariens
A−nobile_TI
A−valverder
A−gomerens
A−decorum
A−castello
A−percarnei
A−lancerotte
A−leucoblep
A−leucoblep
A−stuessyi
A−gorgonet
A−pseudurb
A−hierrense
A−escobarii
A−davidbrar
Aeo_hawort
A_urbicum
A−urbicum
A−volkerii
A−mascaen
A−ciliatum
A−appendic
A−sedifolium
A−sedifolium
A−saunders
A−spathulat
A−smithii_m
A−diplocyclu
A−dodrantal
A−aureum
A−aizoon
8
6
4
2
0
Millions of years ago

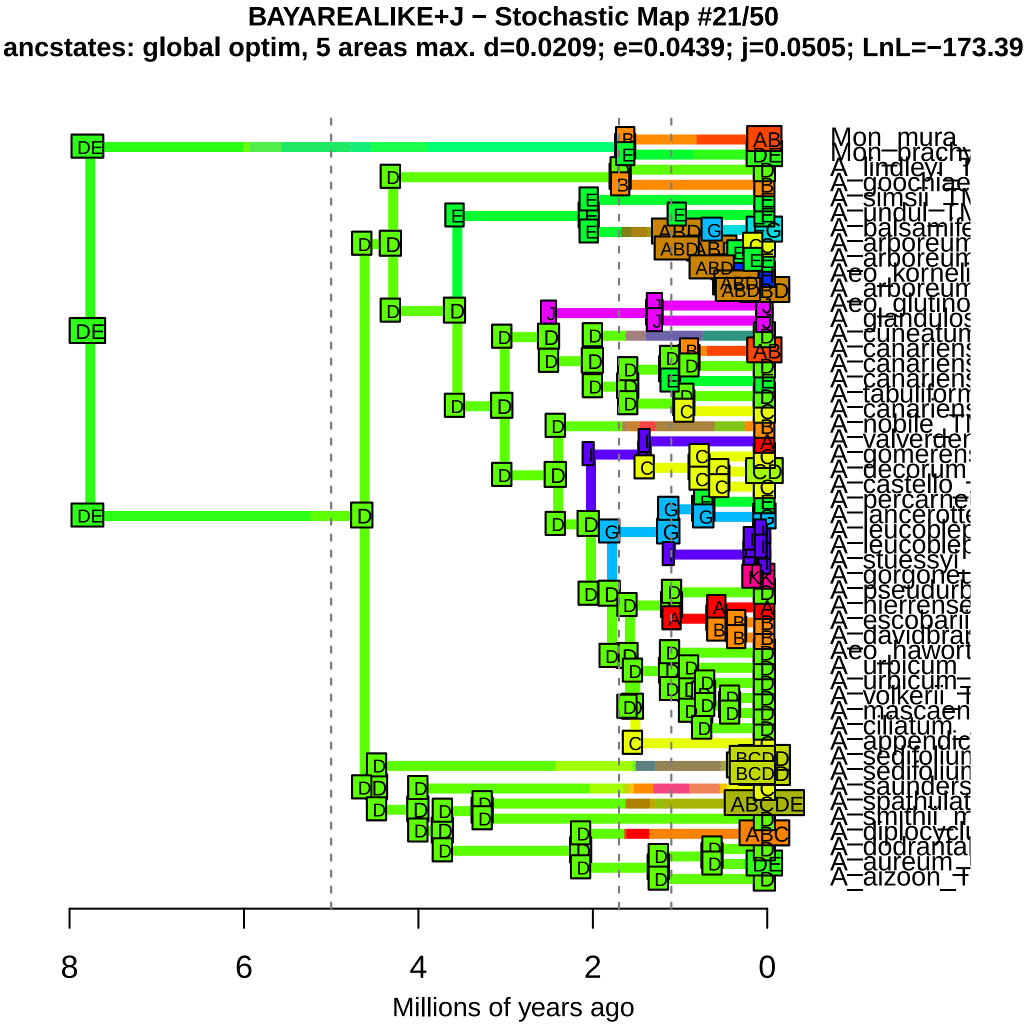
BAYAREALIKE+J − Stochastic Map #21/50
ancstates: global optim, 5 areas max. d=0.0209; e=0.0439; j=0.0505; LnL=−173.39
Mon_mura
Mon−brachy
A_lindleyi
A−goochiae
A−simsii
A−undul
A−balsamife
A−arboreum
A−arboreum
Aeo_korneli
A_arboreum
Aeo_glutino
A_glandulos
A−cuneatum
A−canariens
A−canariens
A−canariens
A−tabuliform
A−canariens
A−nobile
A−valverder
A−gomerens
A−decorum
A−castello
A−percarnei
A−lancerotte
A−leucoblep
A−leucoblep
A−stuessyi
A−gorgonet
A−pseudurb
A−hierrense
A−escobarii
A−davidbrar
Aeo_hawort
A_urbicum
A−urbicum
A−volkerii
A−mascaen
A−ciliatum
A−appendic
A−sedifoliun
A−sedifoliun
A−saunders
A−spathulat
A−smithii
A−diplocyclu
A−dodranta
A−aureum
A_aizoon
8
6
4
2
0
Millions of years ago

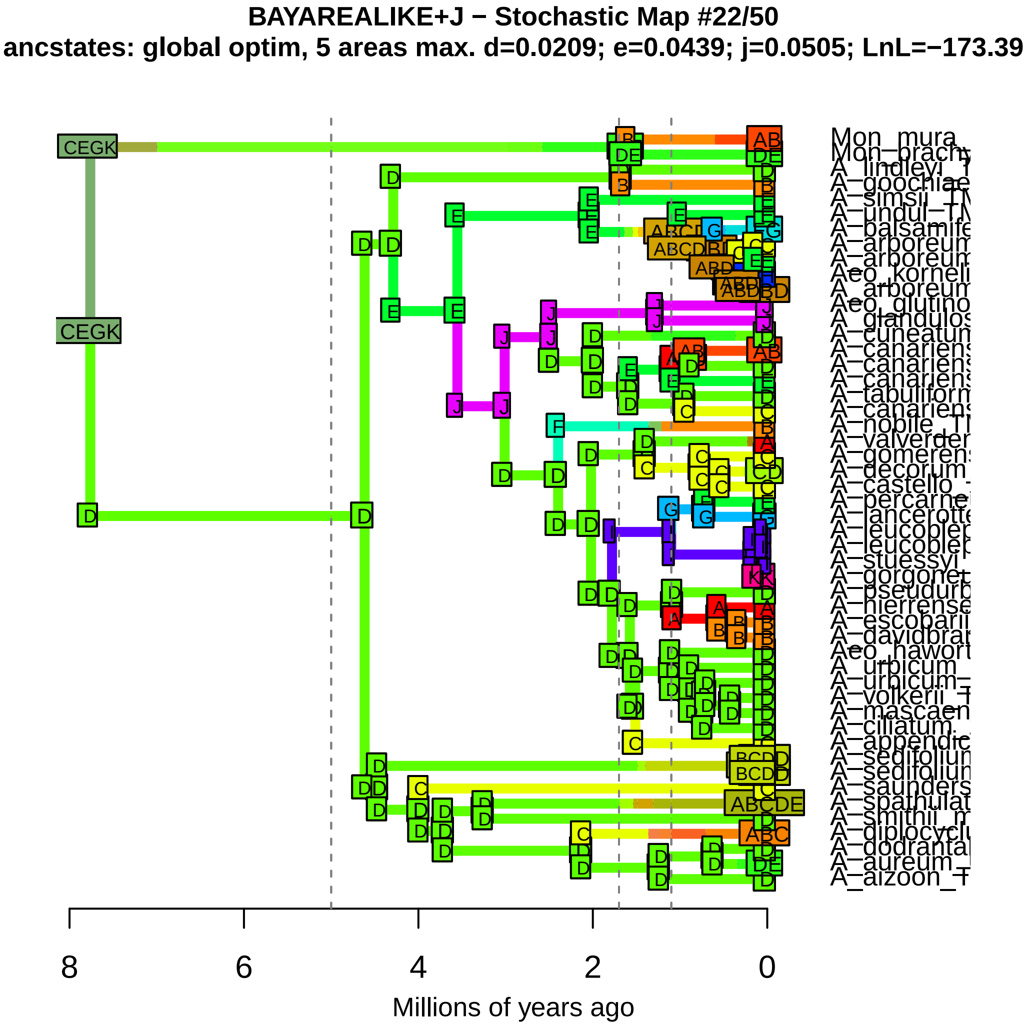

BAYAREALIKE+J − Stochastic Map #22/50
ancstates: global optim, 5 areas max. d=0.0209; e=0.0439; j=0.0505; LnL=−173.39
CEGK
Mon_mura
Mon−brachy
A_lindleyi_T
A−goochiae
A−simsii_TM
A−undul−TM
A−balsamife
A−arboreum
A−arboreum
Aeo_korneli
A_arboreum
Aeo_glutino
A_glandulos
A−cuneatum
A−canariens
A−canariens
A−canariens
A−tabuliform
A−canariens
A−nobile_TM
A−valverder
A−gomerens
A−decorum
A−castello
A−percarnei
A−lancerotte
A−leucoblep
A−leucoblep
A−stuessyi
A−gorgonet
A−pseudurb
A−hierrense
A−escobarii
A−davidbrar
Aeo_hawort
A_urbicum
A−urbicum
A−volkerii
A−mascaen
A−ciliatum
A−appendic
A−sedifoliun
A−sedifoliun
A−saunders
A−spathulat
A−smithii
A−diplocycl
A−dodrantal
A−aureum
A_aizoon
8
6
4
2
0
Millions of years ago

BAYAREALIKE+J − Stochastic Map #23/50
ancstates: global optim, 5 areas max. d=0.0209; e=0.0439; j=0.0505; LnL=−173.39

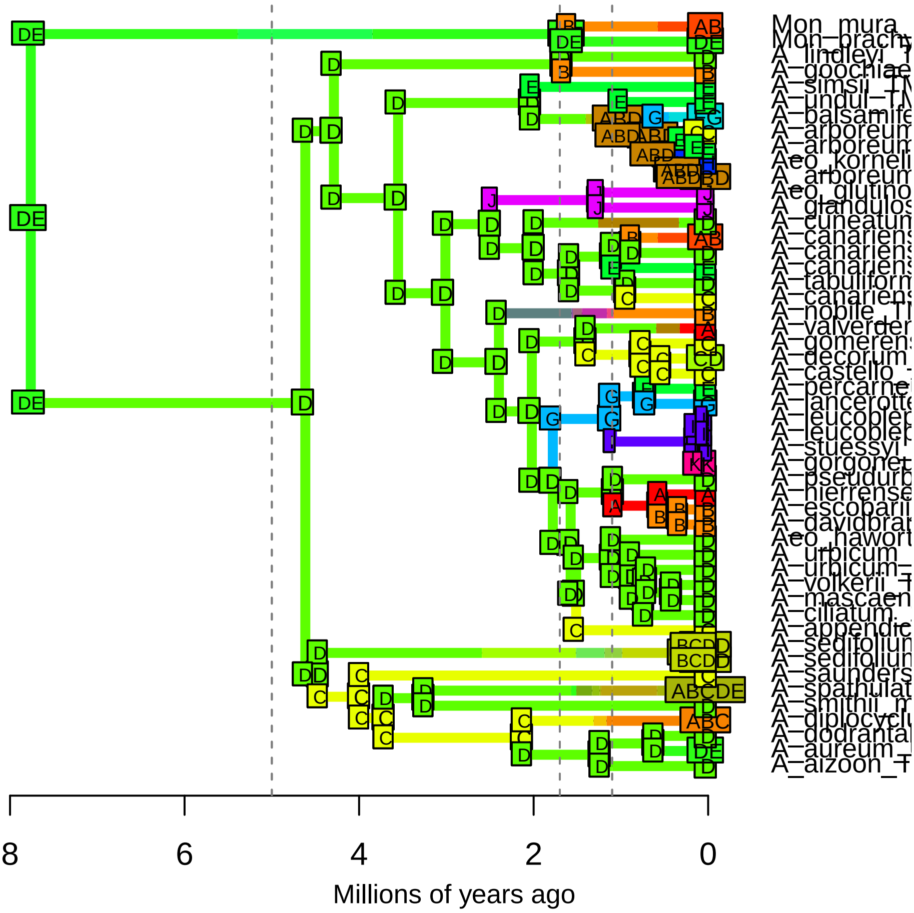

BAYAREALIKE+J − Stochastic Map #24/50
ancstates: global optim, 5 areas max. d=0.0209; e=0.0439; j=0.0505; LnL=−173.39

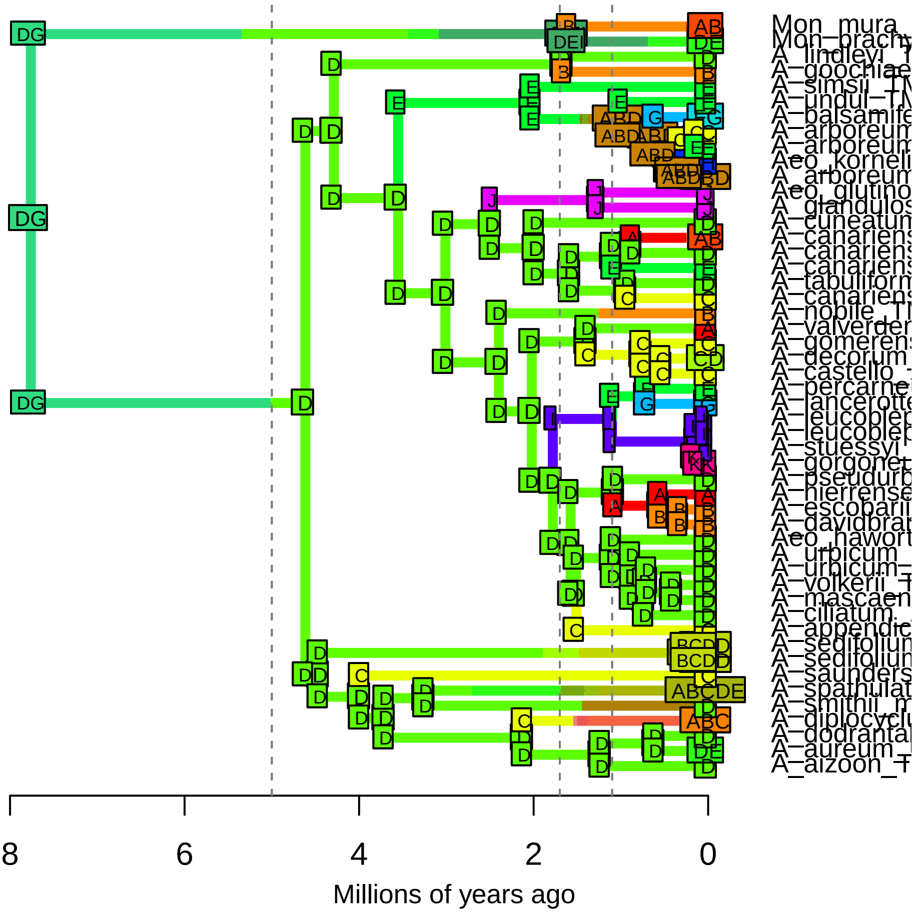

BAYAREALIKE+J − Stochastic Map #25/50
ancstates: global optim, 5 areas max. d=0.0209; e=0.0439; j=0.0505; LnL=−173.39

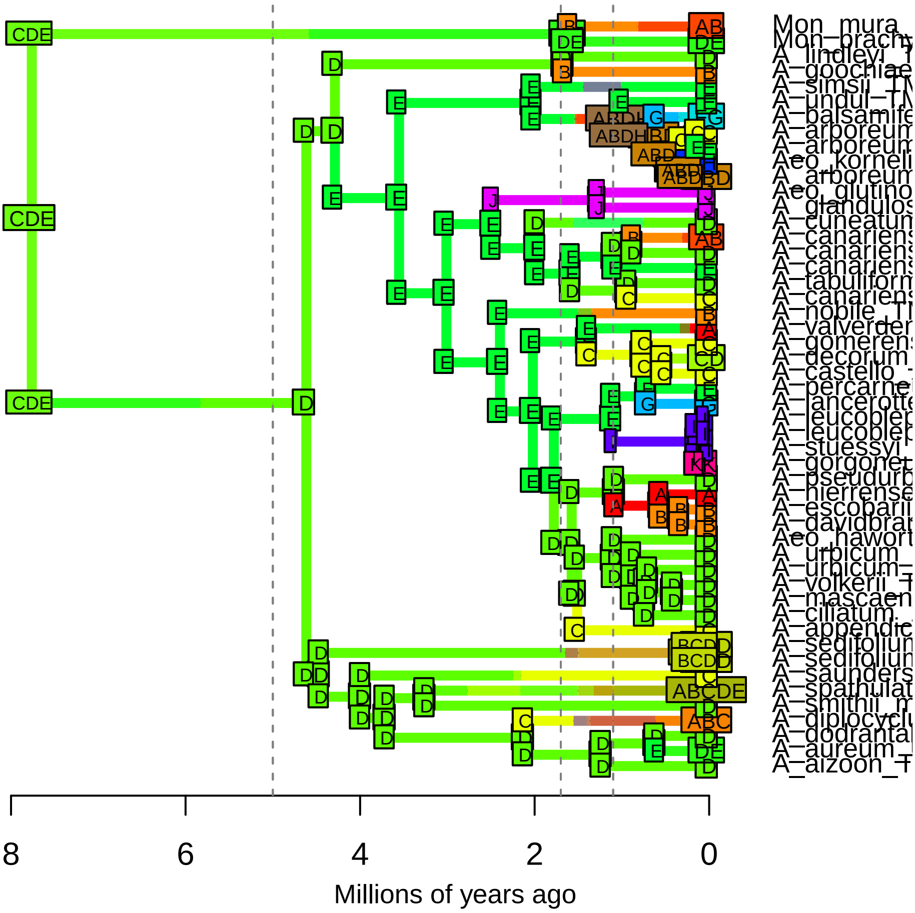

BAYAREALIKE+J − Stochastic Map #26/50
ancstates: global optim, 5 areas max. d=0.0209; e=0.0439; j=0.0505; LnL=−173.39

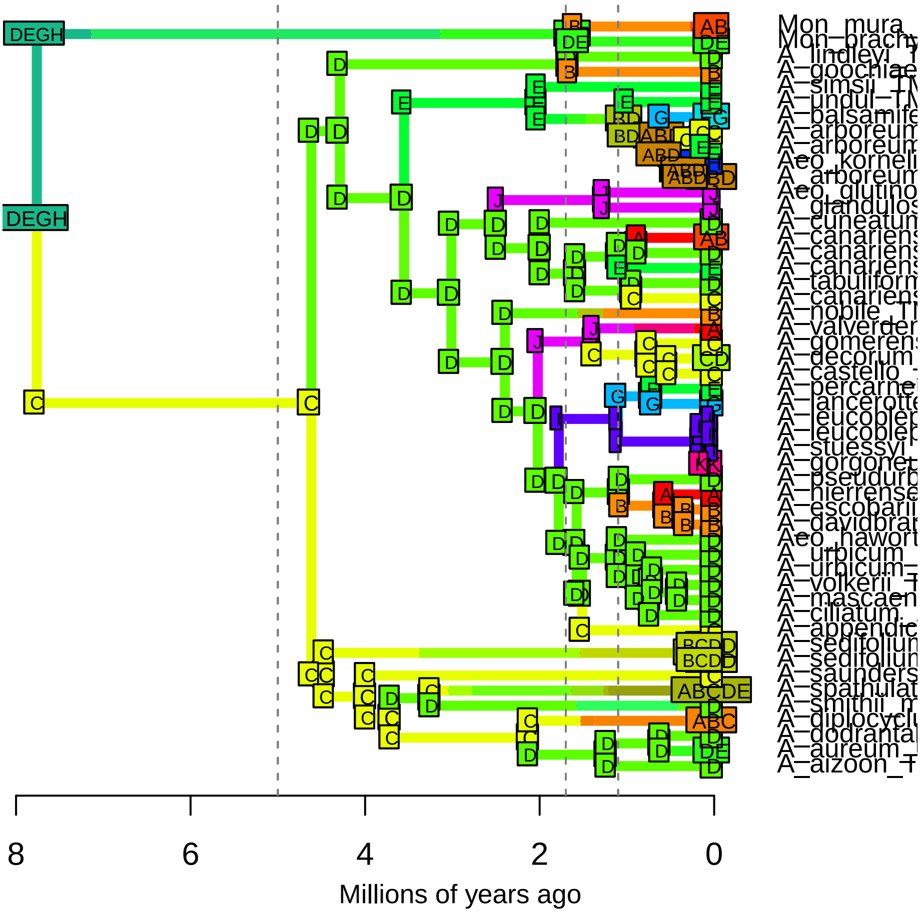

BAYAREALIKE+J − Stochastic Map #27/50
ancstates: global optim, 5 areas max. d=0.0209; e=0.0439; j=0.0505; LnL=−173.39

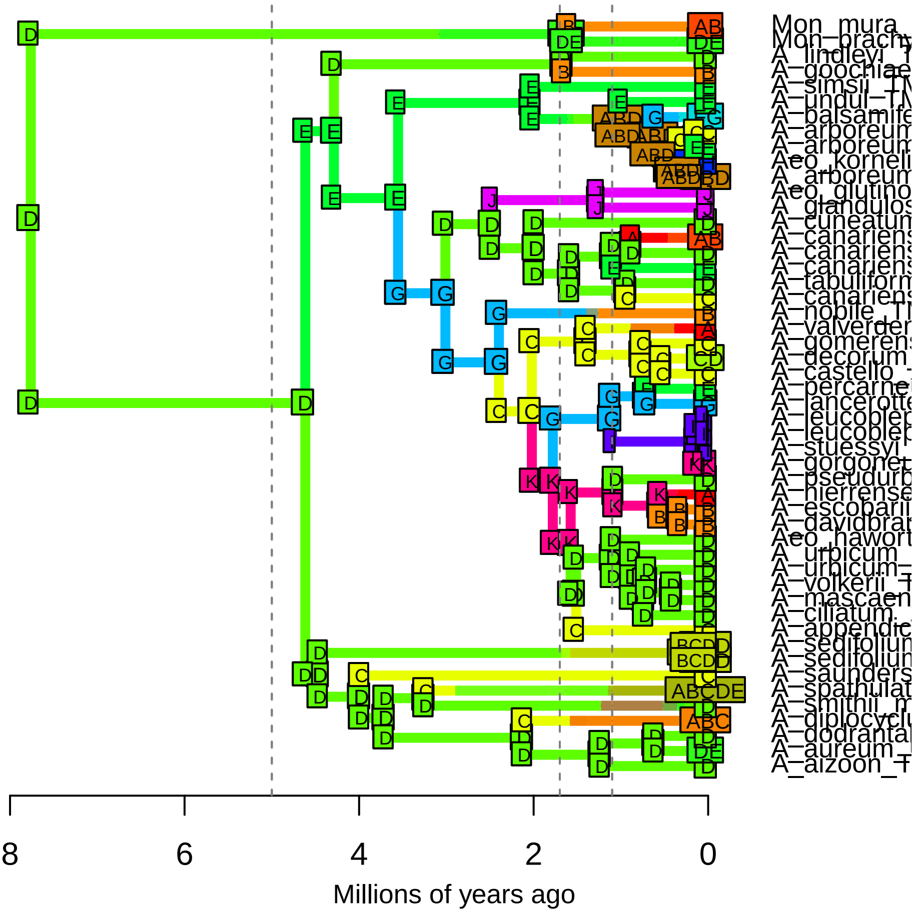

BAYAREALIKE+J − Stochastic Map #28/50
ancstates: global optim, 5 areas max. d=0.0209; e=0.0439; j=0.0505; LnL=−173.39

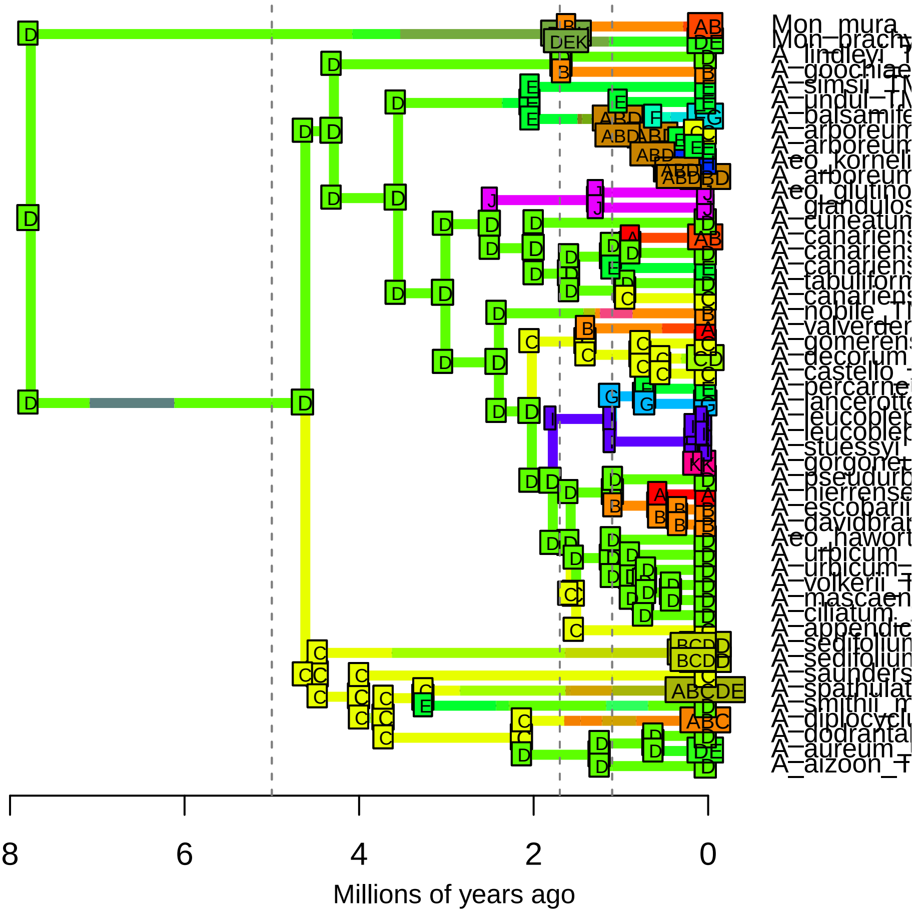

BAYAREALIKE+J − Stochastic Map #29/50
ancstates: global optim, 5 areas max. d=0.0209; e=0.0439; j=0.0505; LnL=−173.39

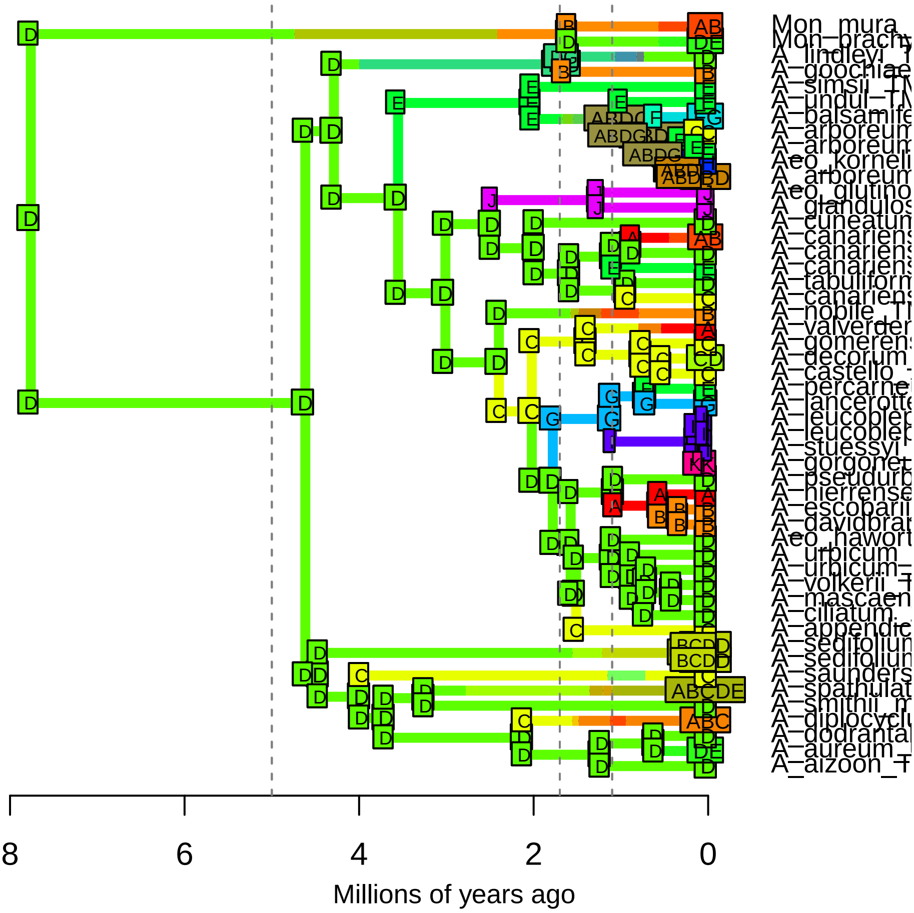

BAYAREALIKE+J − Stochastic Map #30/50
ancstates: global optim, 5 areas max. d=0.0209; e=0.0439; j=0.0505; LnL=−173.39

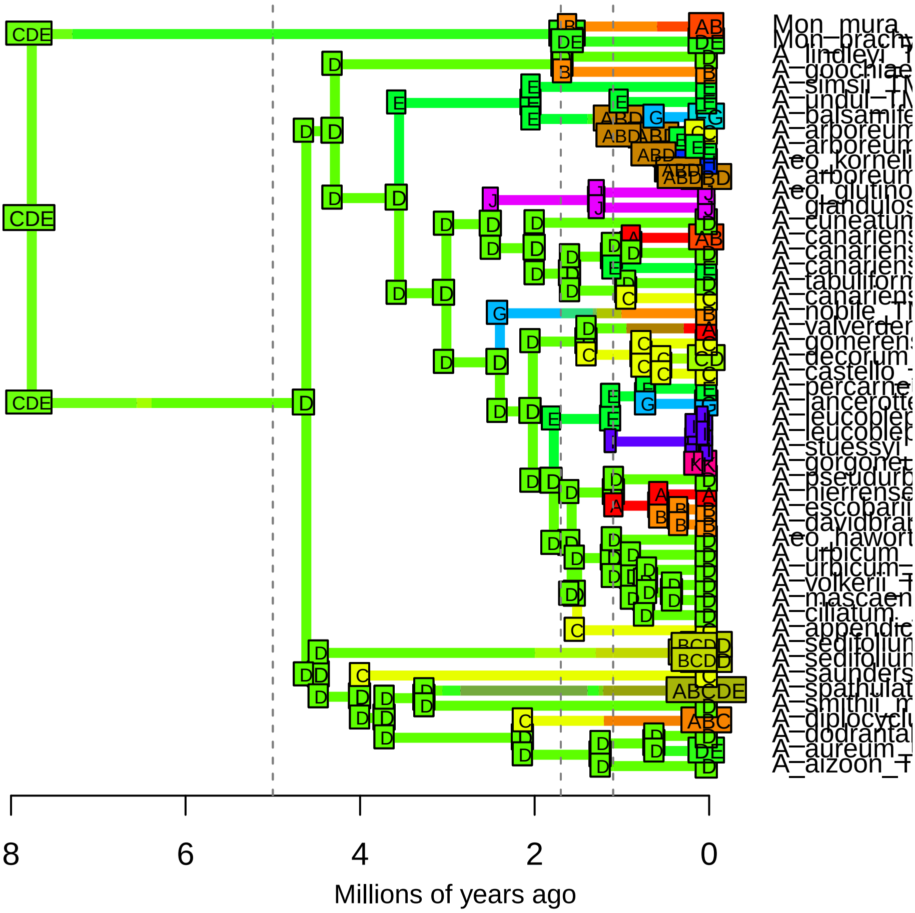

BAYAREALIKE+J − Stochastic Map #31/50
ancstates: global optim, 5 areas max. d=0.0209; e=0.0439; j=0.0505; LnL=−173.39

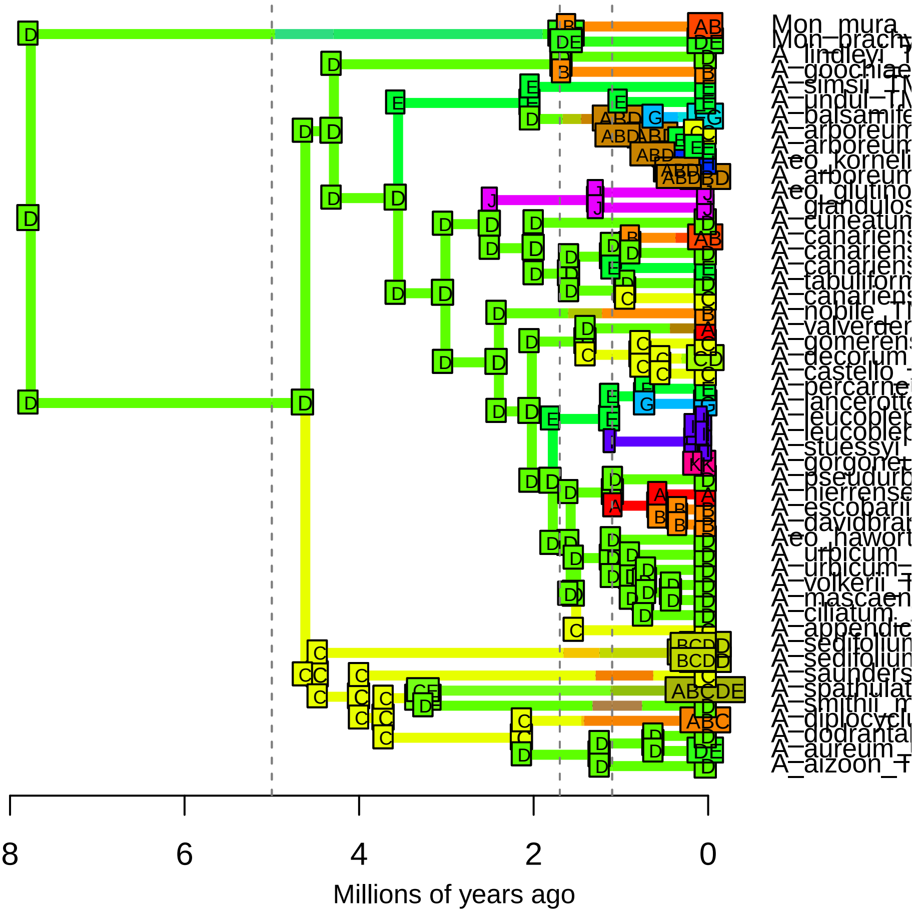

BAYAREALIKE+J − Stochastic Map #32/50
ancstates: global optim, 5 areas max. d=0.0209; e=0.0439; j=0.0505; LnL=−173.39

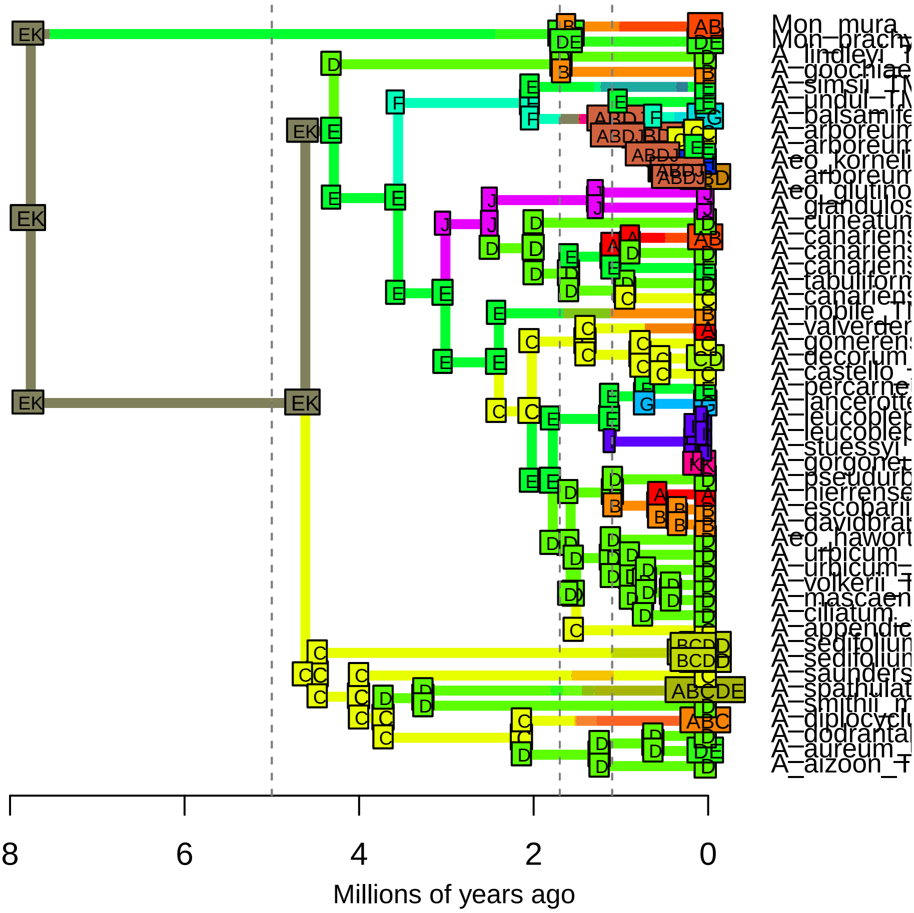

BAYAREALIKE+J − Stochastic Map #33/50

ancstates: global optim, 5 areas max. d=0.0209; e=0.0439; j=0.0505; LnL=−173.39

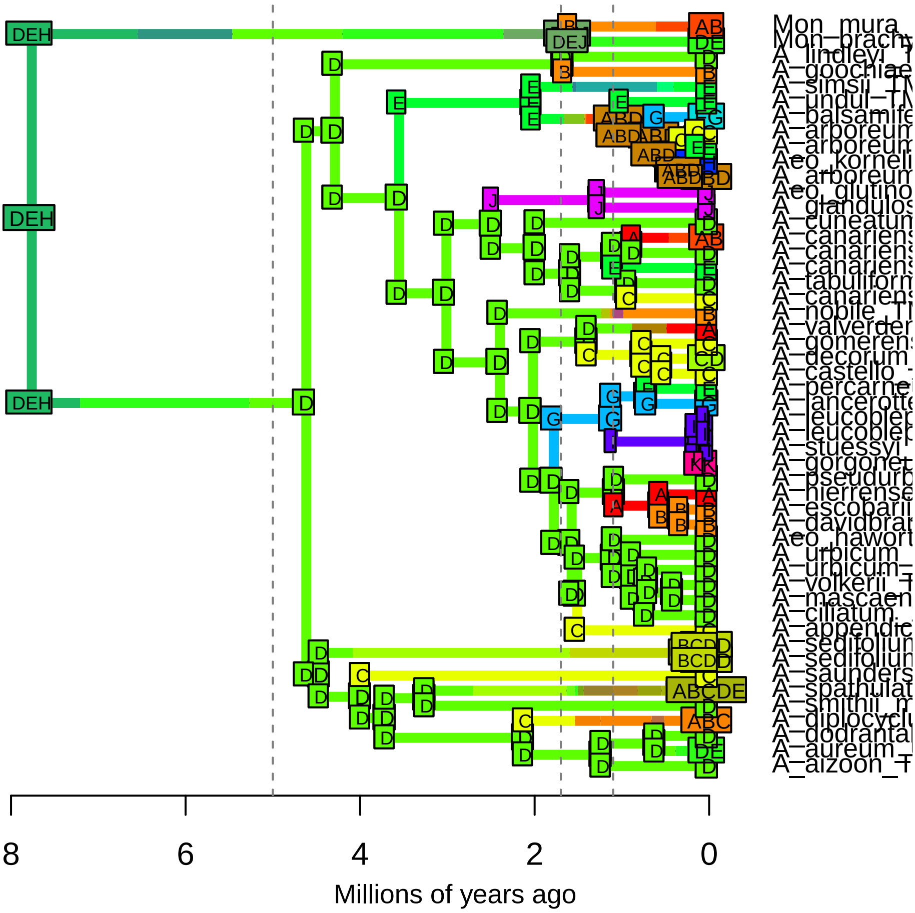

BAYAREALIKE+J − Stochastic Map #34/50
ancstates: global optim, 5 areas max. d=0.0209; e=0.0439; j=0.0505; LnL=−173.39

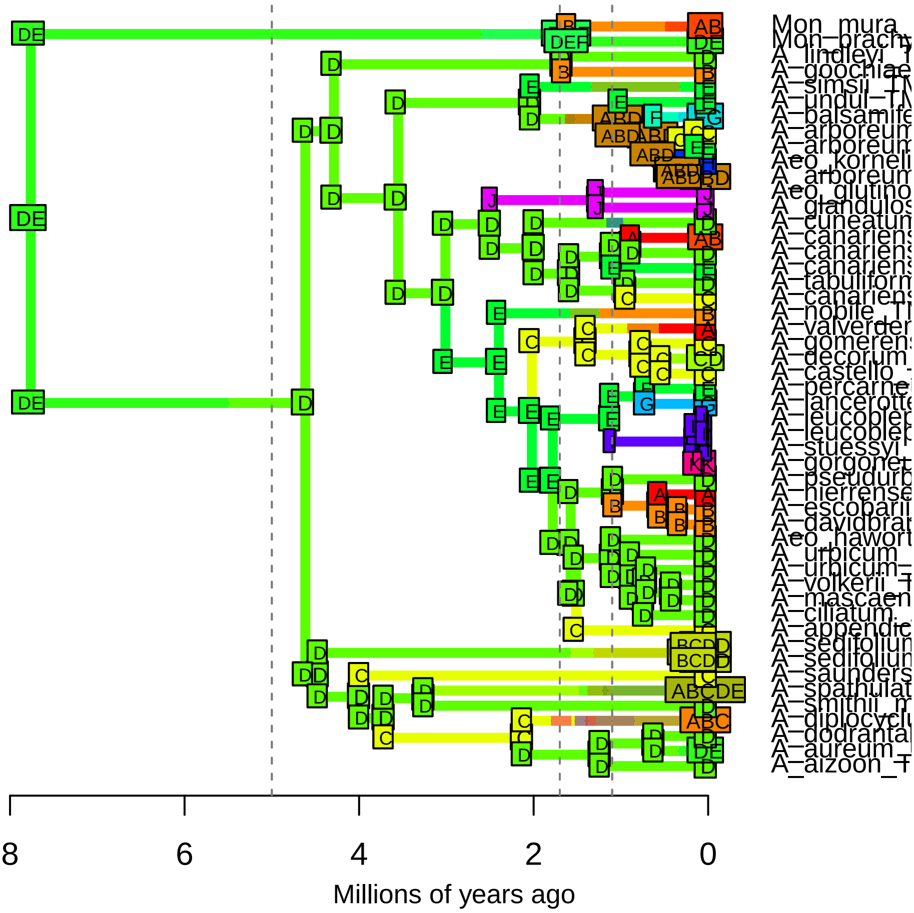

BAYAREALIKE+J − Stochastic Map #35/50
ancstates: global optim, 5 areas max. d=0.0209; e=0.0439; j=0.0505; LnL=−173.39

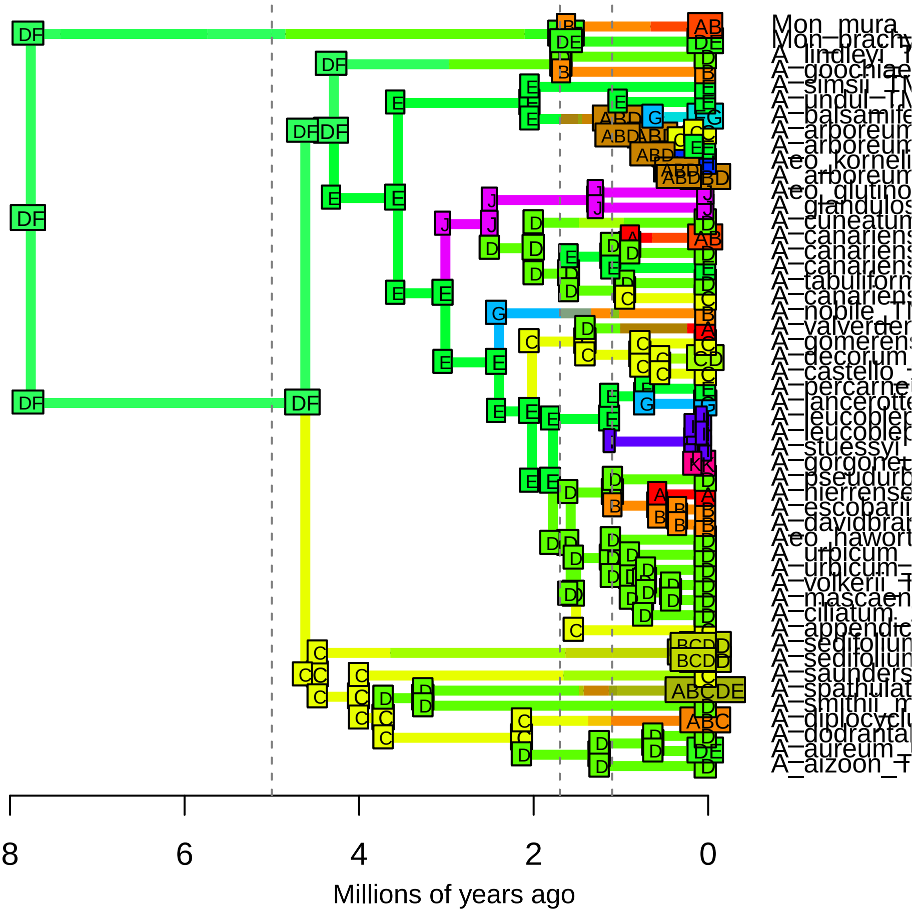

BAYAREALIKE+J − Stochastic Map #36/50
ancstates: global optim, 5 areas max. d=0.0209; e=0.0439; j=0.0505; LnL=−173.39

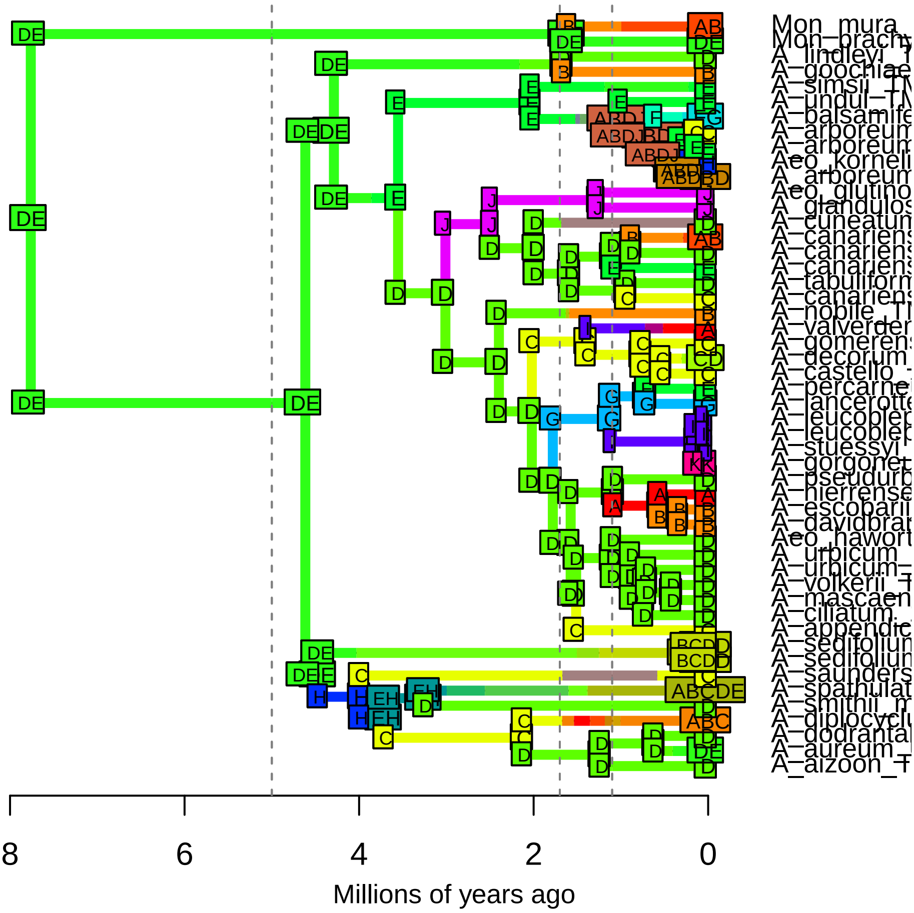

BAYAREALIKE+J − Stochastic Map #37/50
ancstates: global optim, 5 areas max. d=0.0209; e=0.0439; j=0.0505; LnL=−173.39

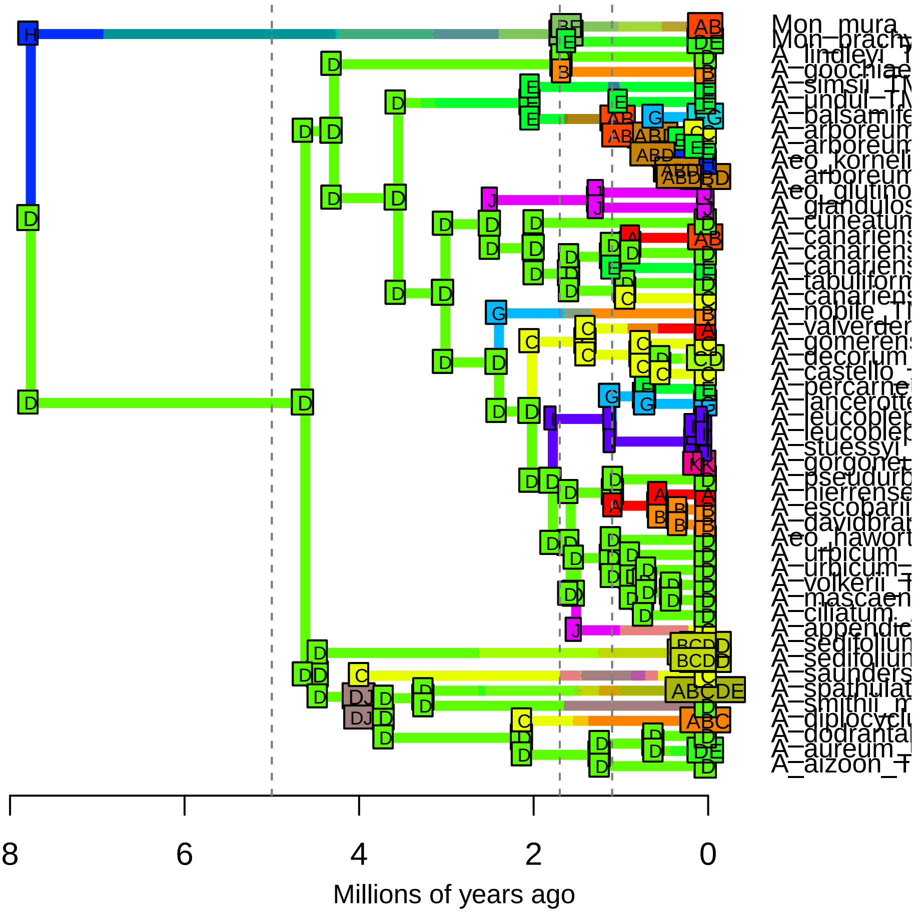

BAYAREALIKE+J − Stochastic Map #38/50
ancstates: global optim, 5 areas max. d=0.0209; e=0.0439; j=0.0505; LnL=−173.39

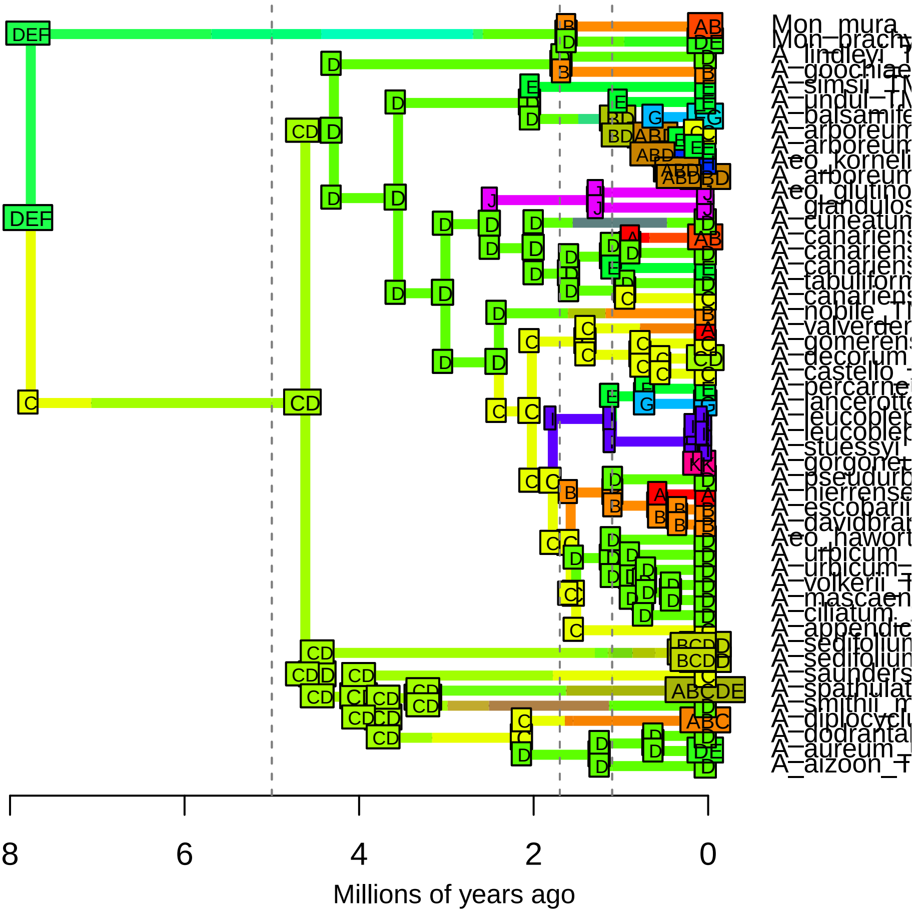

BAYAREALIKE+J − Stochastic Map #39/50

ancstates: global optim, 5 areas max. d=0.0209; e=0.0439; j=0.0505; LnL=−173.39

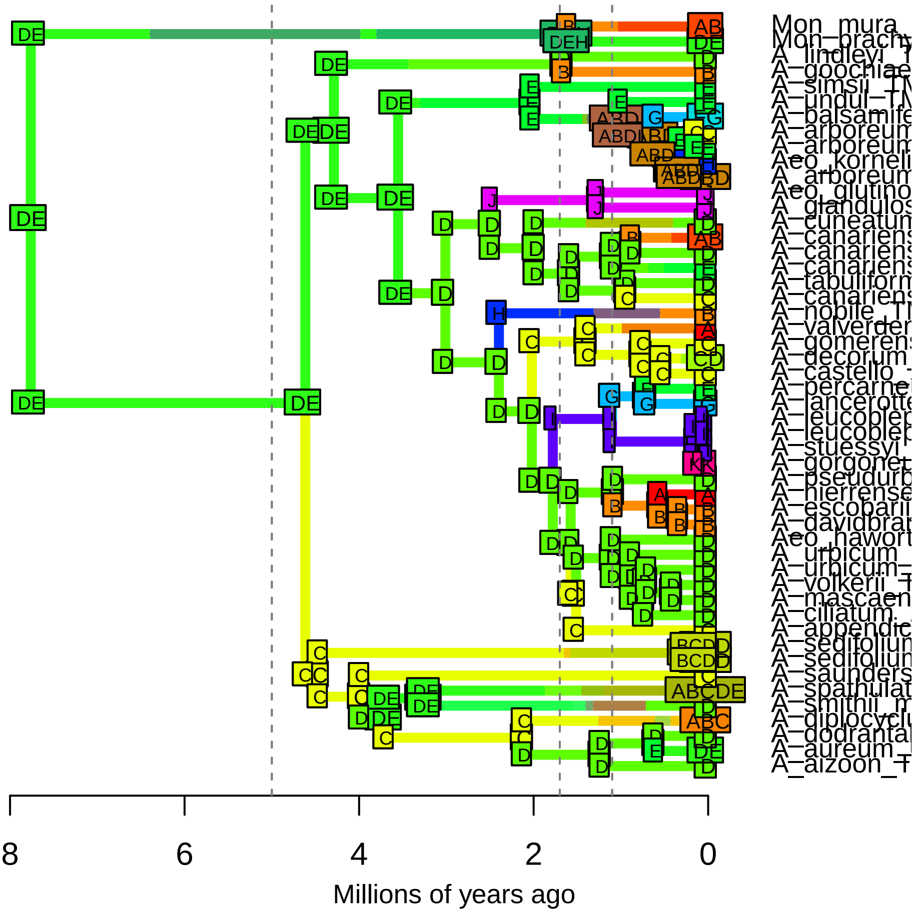

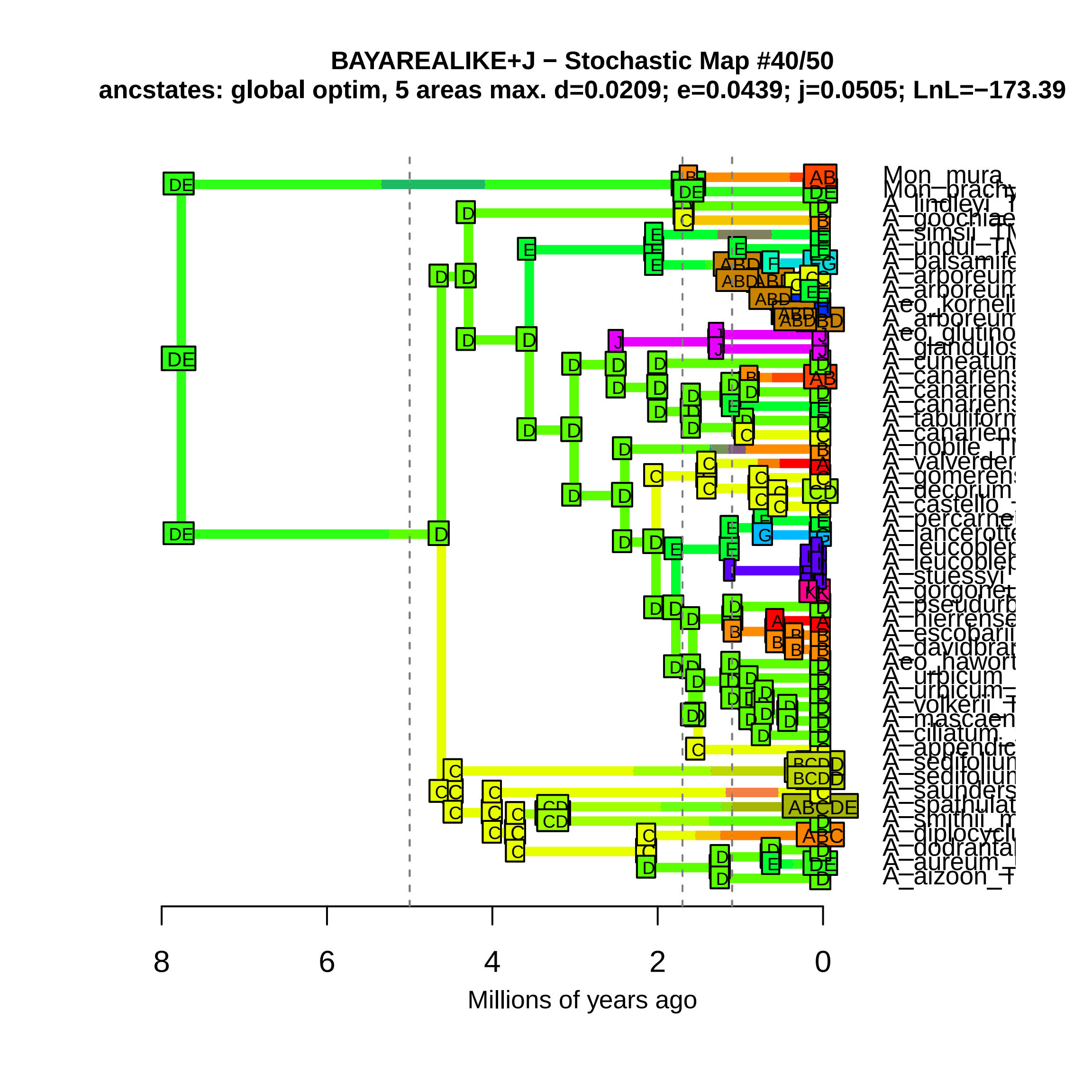

BAYAREALIKE+J − Stochastic Map #40/50
ancstates: global optim, 5 areas max. d=0.0209; e=0.0439; j=0.0505; LnL=−173.39
Mon_mura
Mon−brachy
A_lindleyi
A−goochiae
A−simsii
A−undul
A−balsamife
A−arboreum
A−arboreum
Aeo_korneli
A_arboreum
Aeo_glutino
A_glandulos
A−cuneatum
A−canariens
A−canariens
A−canariens
A−tabuliform
A−canariens
A−nobile
A−valverder
A−gomerens
A−decorum
A−castello
A−percarnei
A−lancerotte
A−leucoblep
A−leucoblep
A−stuessyi
A−gorgonet
A−pseudurb
A−hierrense
A−escobarii
A−davidbrar
Aeo_hawort
A_urbicum
A−urbicum
A−volkerii
A−mascaen
A−ciliatum
A−appendic
A−sedifolium
A−sedifolium
A−saunders
A−spathulat
A−smithii
A−diplocycl
A−dodranta
A−aureum
A_aizoon
8
6
4
2
0
Millions of years ago

BAYAREALIKE+J − Stochastic Map #41/50

ancstates: global optim, 5 areas max. d=0.0209; e=0.0439; j=0.0505; LnL=−173.39

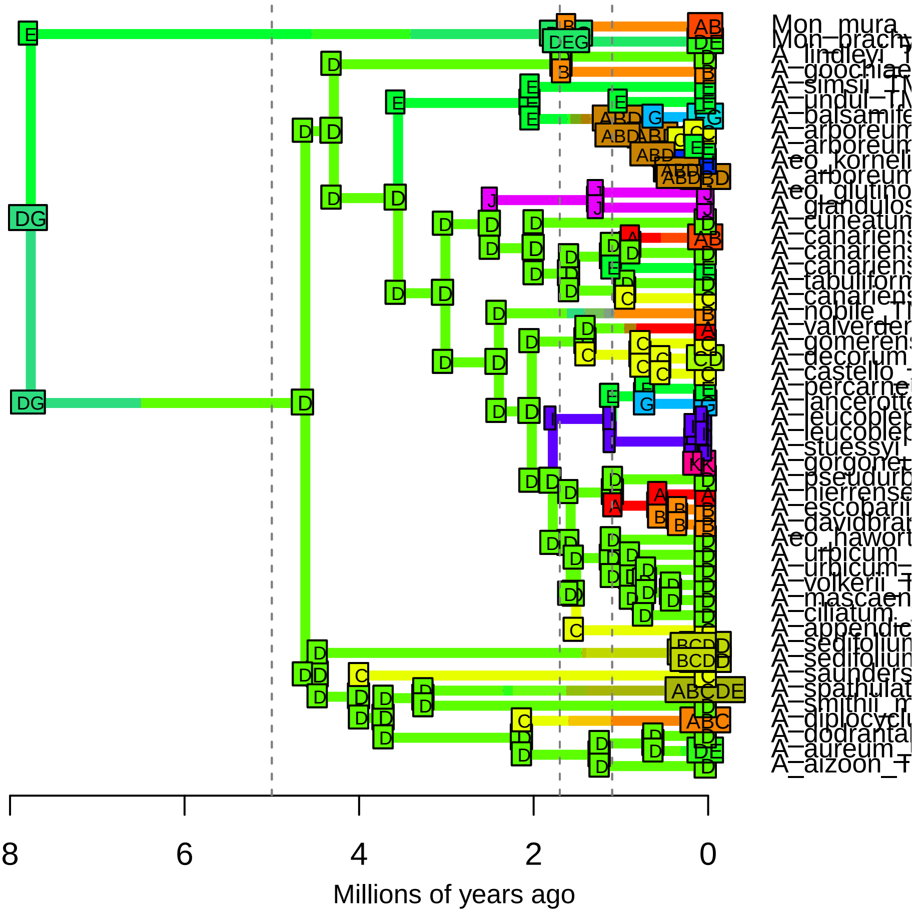

BAYAREALIKE+J − Stochastic Map #42/50
ancstates: global optim, 5 areas max. d=0.0209; e=0.0439; j=0.0505; LnL=−173.39

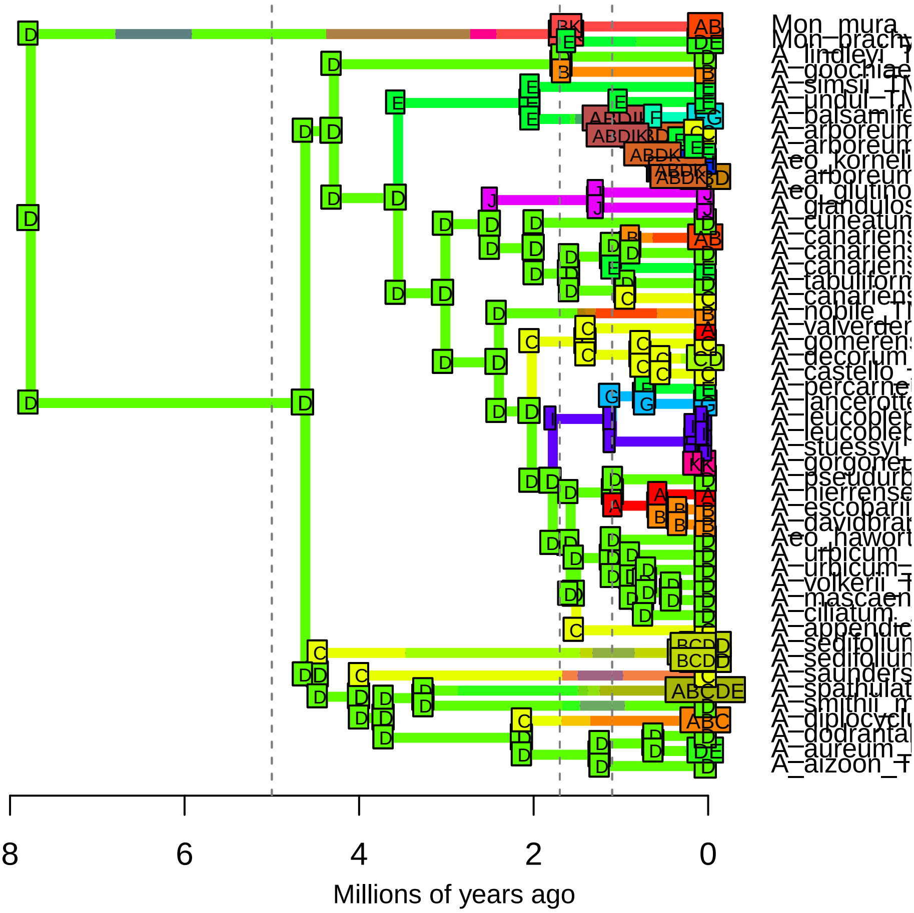

BAYAREALIKE+J − Stochastic Map #43/50
ancstates: global optim, 5 areas max. d=0.0209; e=0.0439; j=0.0505; LnL=−173.39

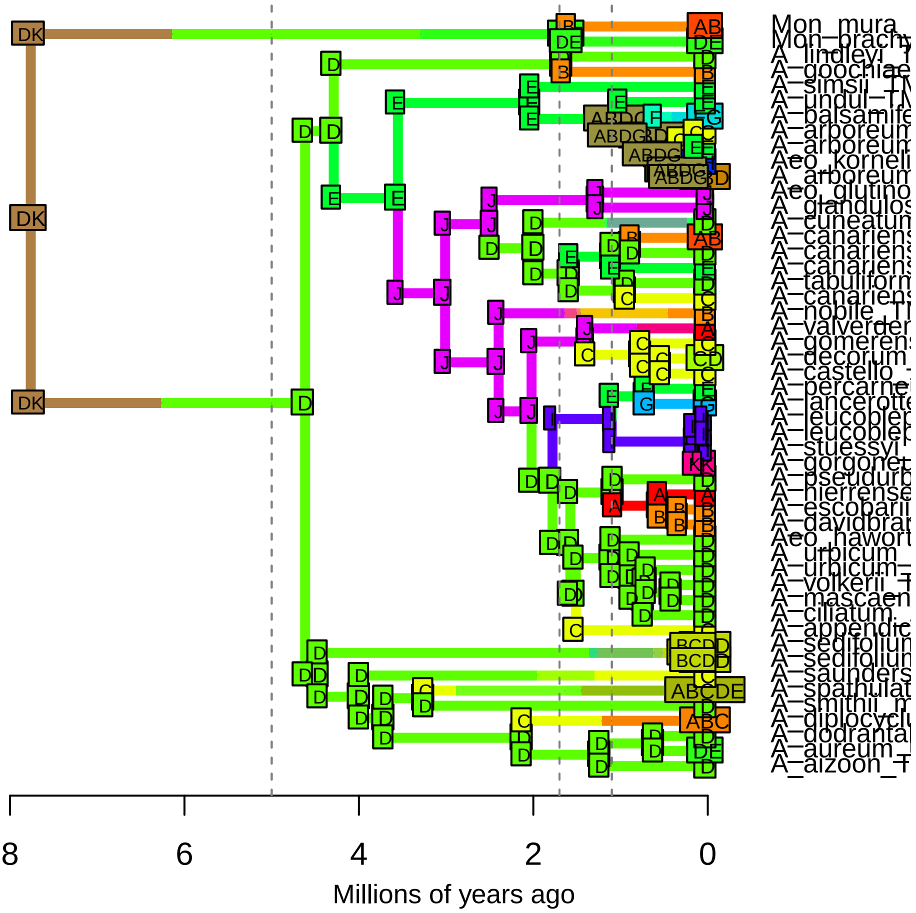

BAYAREALIKE+J − Stochastic Map #44/50
ancstates: global optim, 5 areas max. d=0.0209; e=0.0439; j=0.0505; LnL=−173.39

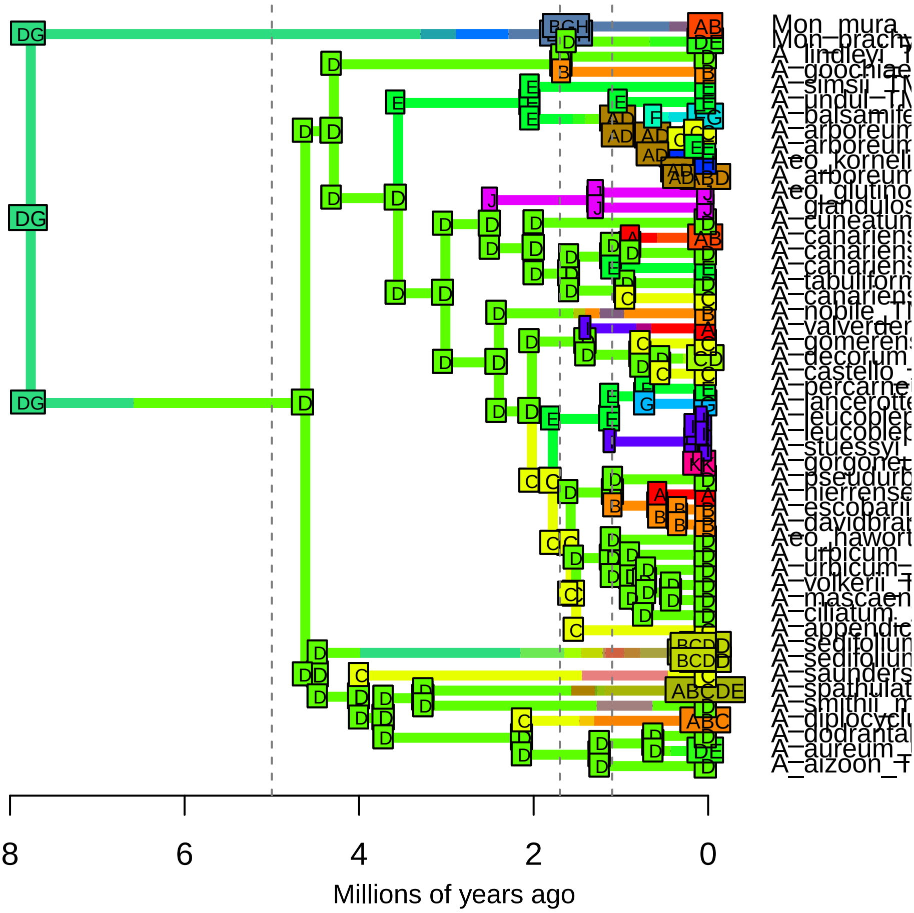

BAYAREALIKE+J − Stochastic Map #45/50

ancstates: global optim, 5 areas max. d=0.0209; e=0.0439; j=0.0505; LnL=−173.39

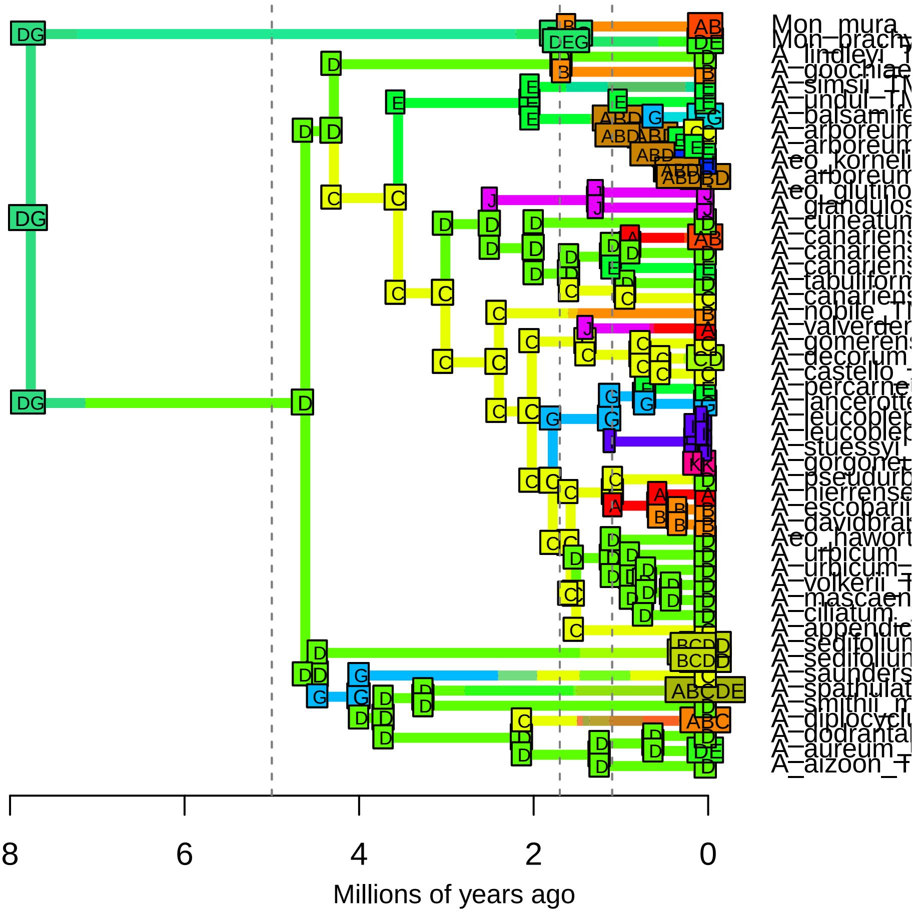

BAYAREALIKE+J − Stochastic Map #46/50
ancstates: global optim, 5 areas max. d=0.0209; e=0.0439; j=0.0505; LnL=−173.39

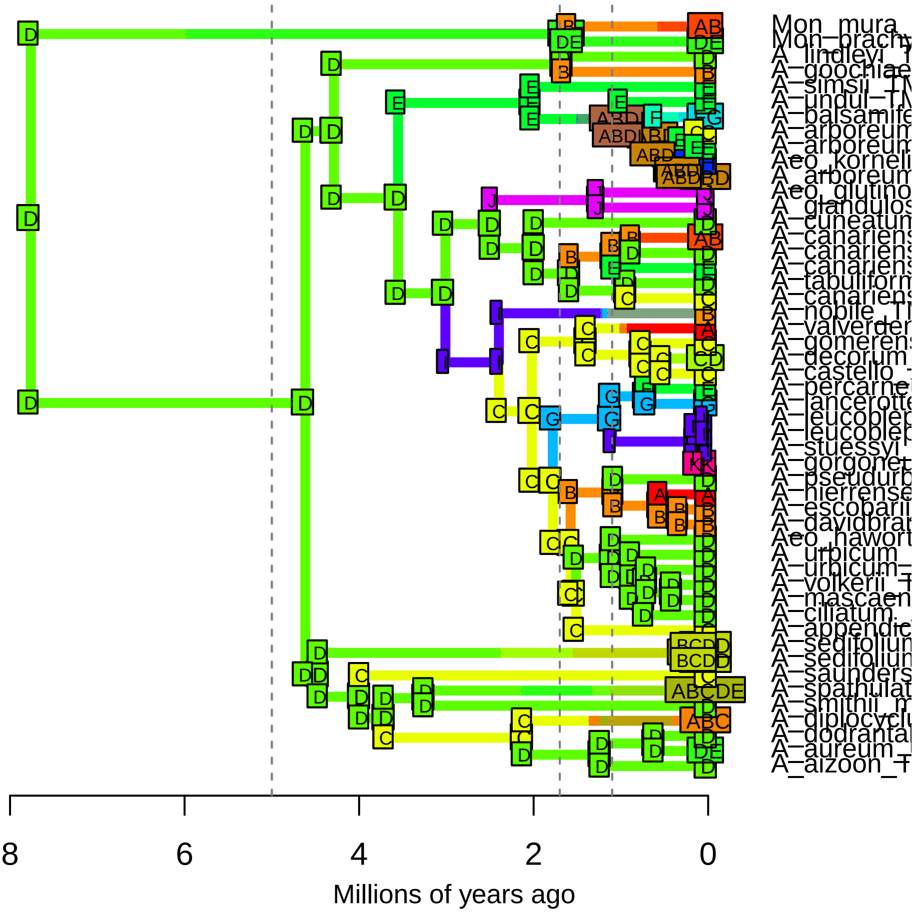

BAYAREALIKE+J − Stochastic Map #47/50
ancstates: global optim, 5 areas max. d=0.0209; e=0.0439; j=0.0505; LnL=−173.39

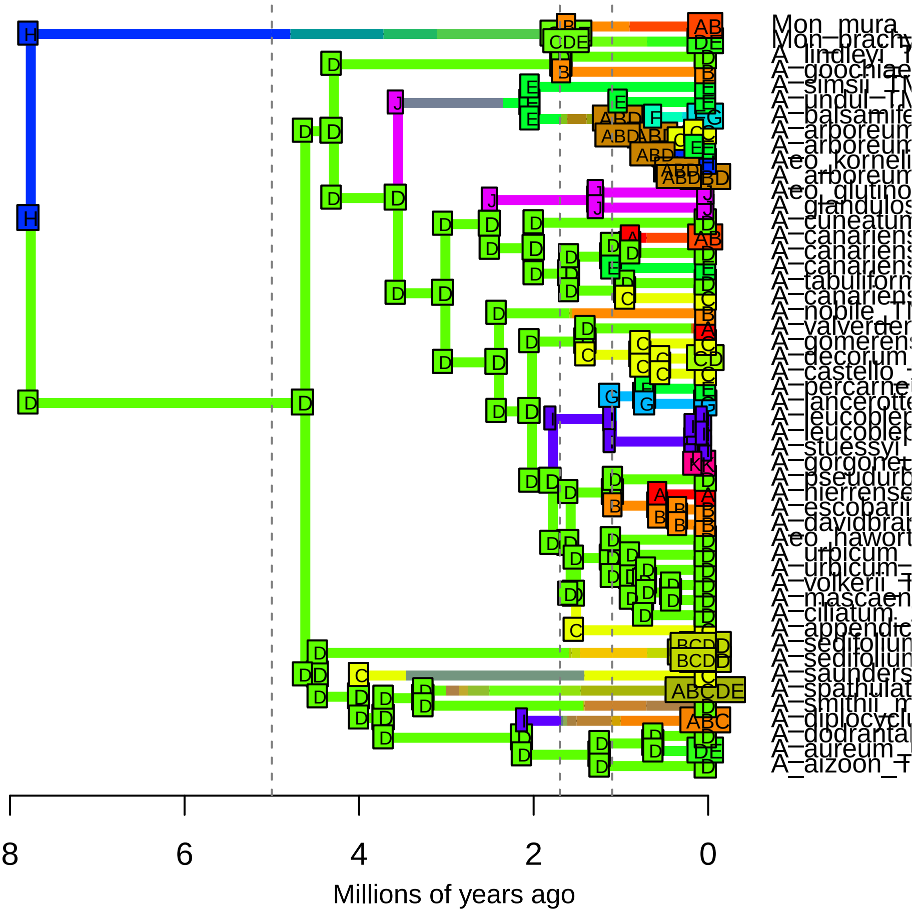

BAYAREALIKE+J − Stochastic Map #48/50
ancstates: global optim, 5 areas max. d=0.0209; e=0.0439; j=0.0505; LnL=−173.39

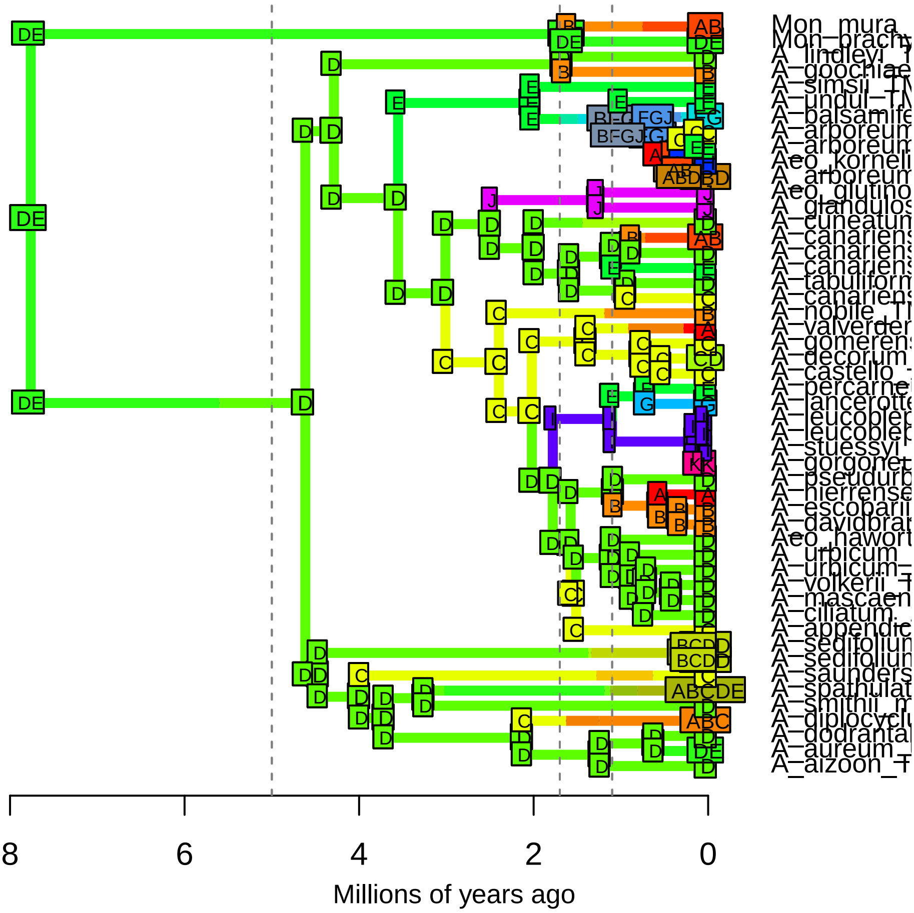

BAYAREALIKE+J − Stochastic Map #49/50
ancstates: global optim, 5 areas max. d=0.0209; e=0.0439; j=0.0505; LnL=−173.39

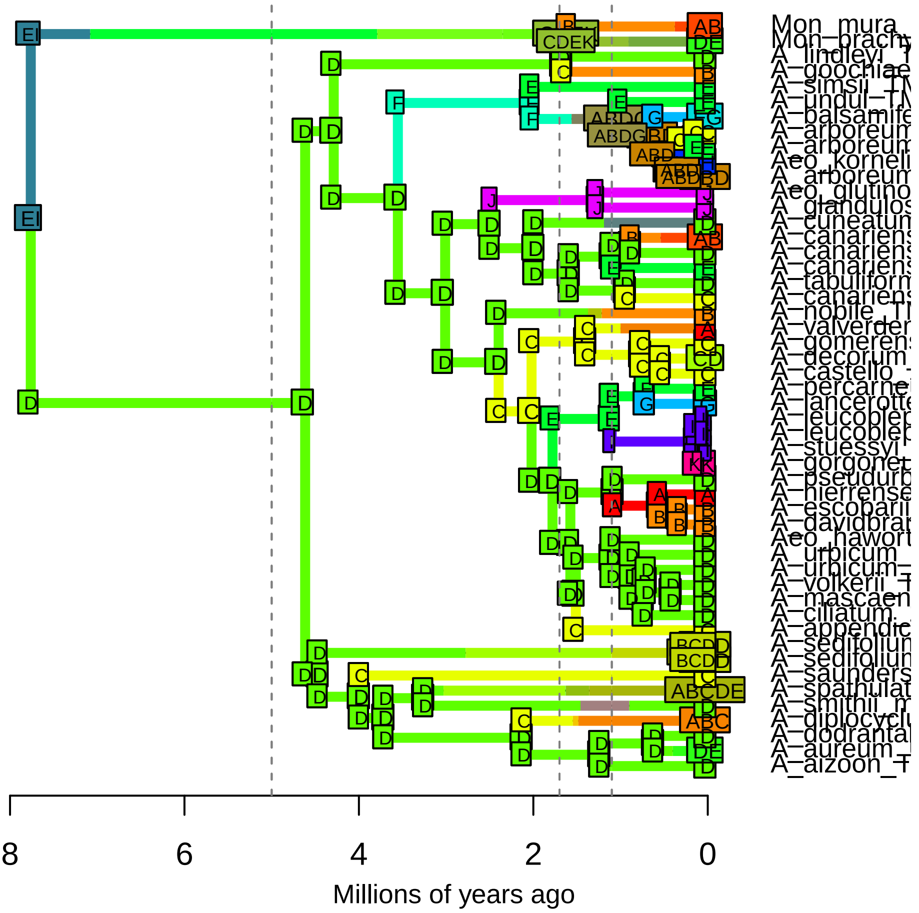

BAYAREALIKE+J − Stochastic Map #50/50
ancstates: global optim, 5 areas max. d=0.0209; e=0.0439; j=0.0505; LnL=−173.39

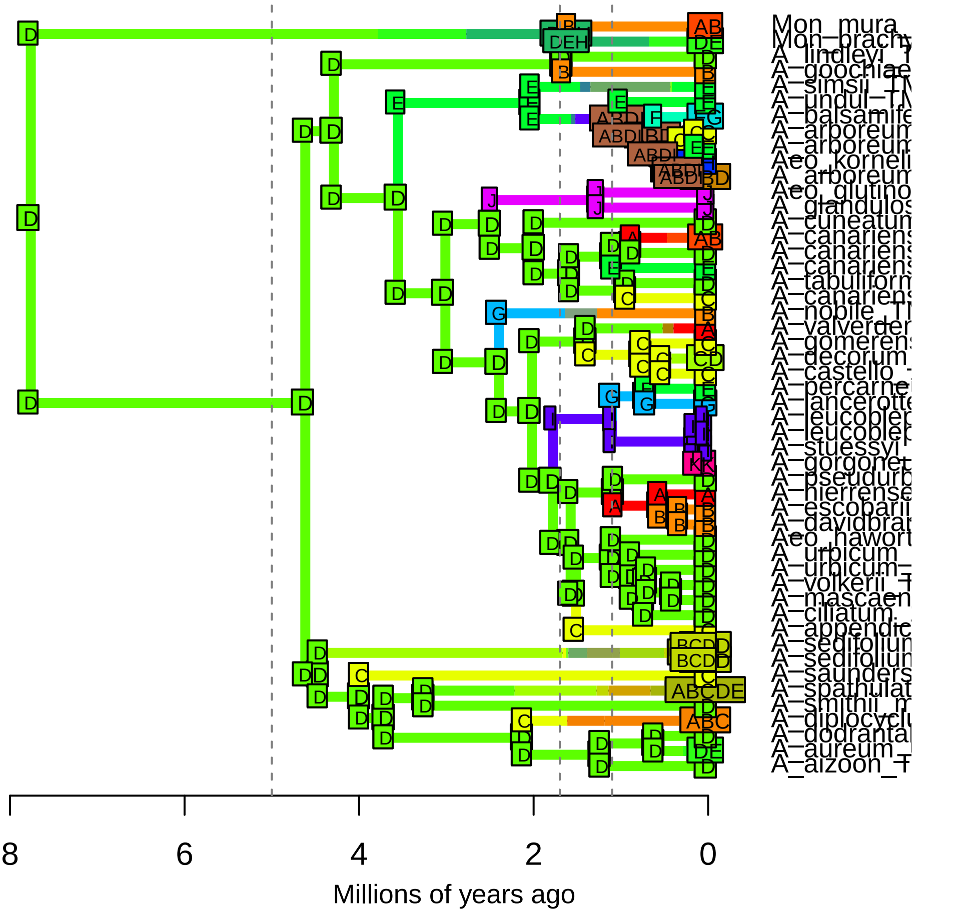

Supplement: mcad033_suppl_Supplementary_Data_S4 [file mcad033_suppl_supplementary_data_s4.pdf]
