# Supplementary figures and images for: Inter- and intra-island speciation and their morphological and ecological correlates in Aeonium (Crassulaceae), a species-rich Macaronesian radiation
Source: Ann Bot. 2023 Feb 23;131(4):697–721. doi: 10.1093/aob/mcad033 (PMC10147336; doi:10.1093/aob/mcad033)

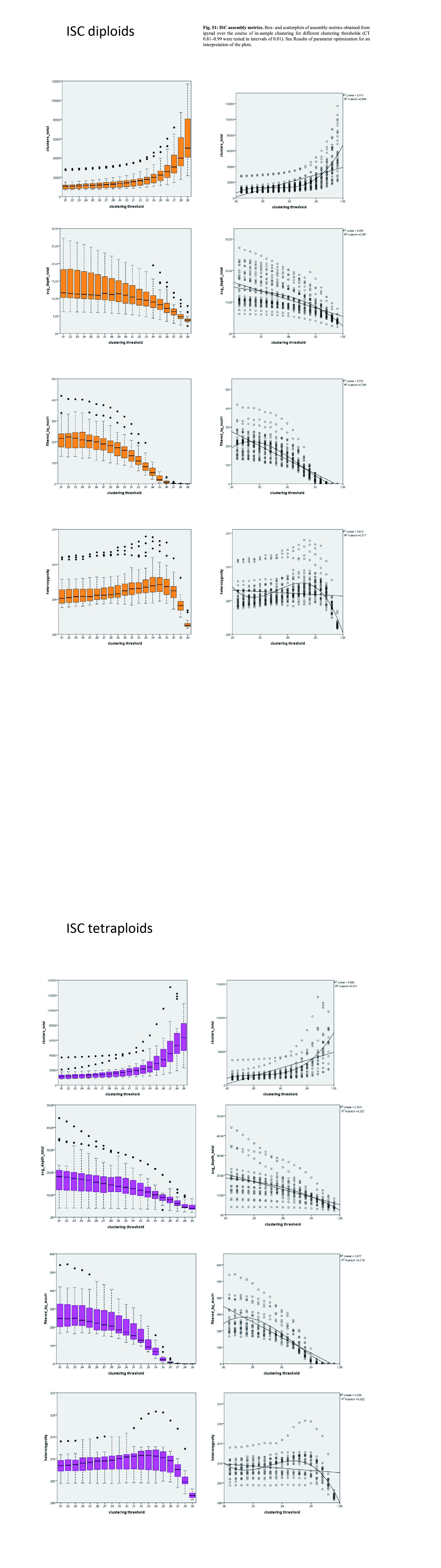

Supplement: mcad033_suppl_Supplementary_Figure_S1 [file mcad033_suppl_supplementary_figure_s1.jpeg]

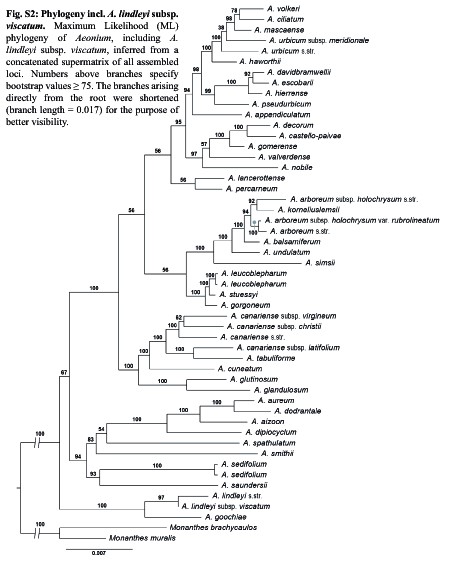

Supplement: mcad033_suppl_Supplementary_Figure_S2 [file mcad033_suppl_supplementary_figure_s2.jpeg]

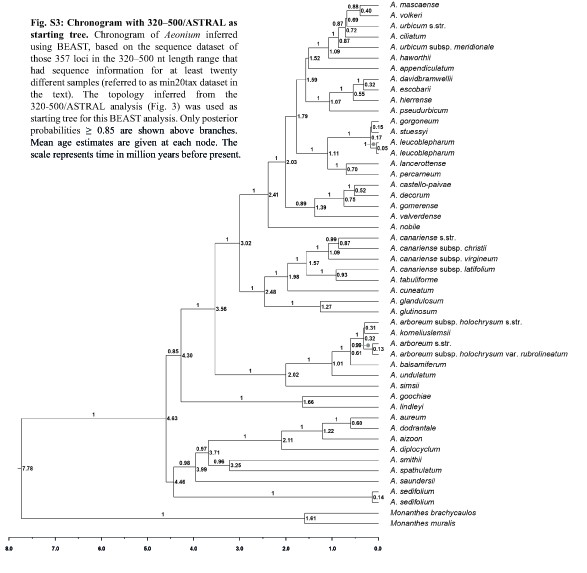

Supplement: mcad033_suppl_Supplementary_Figure_S3 [file mcad033_suppl_supplementary_figure_s3.jpeg]
