# Supplementary material for: Inter- and intra-island speciation and their morphological and ecological correlates in Aeonium (Crassulaceae), a species-rich Macaronesian radiation
Source: Ann Bot. 2023 Feb 23;131(4):697–721. doi: 10.1093/aob/mcad033 (PMC10147336; doi:10.1093/aob/mcad033)
Supplement: mcad033_suppl_Supplementary_Table_S1 [file mcad033_suppl_supplementary_table_s1.docx]

**Table S1:** Detailed information about specimens used in this study.

| Species | Lab code^a^ | GenBank accession number | Material used | Provenience | Voucher |
| --- | --- | --- | --- | --- | --- |
| **Outgroup** | | | | | |
| *Aichryson bituminosum* Bañares | A02_J03^b^ | OM953430 | dried leaves | Spain: Gran Canaria, Roque del Saucillo, 1640 m | *J. Los A02_J03* |
| *Aichryson divaricatum* (Aiton) Praeger | –^b^ | OM953429 | dried leaves | Portugal: Madeira, between Pico do Gato and Macizo de Pico das Torres, 1590–1625 m | *Á. Marrero* (LPA-029204) |
| *Aichryson tortuosum* subsp. *bethencourtianum* (Bolle) Bañares | A37_J50^b^ | OM953428 | dried leaves | Spain: Fuerteventura, Montana de la Muda | *J. Los A37_J50* |
| *Monanthes brachycaulos* (Webb & Berthel.) Lowe | NB73.1 | SRR19745527 | dried leaves | Spain: Gran Canaria, Artanara, 851 m | *N. Bobon M02_N2* |
| *Monanthes minima* (Bolle) Christ | M04_N5^b^ | OM953431 | dried leaves | Spain: Tenerife, Igueste San Andres, 36 m | *N. Bobon M04_N5* |
| *Monanthes muralis* (Webb ex Bolle) Hook.f. | NB111 | SRR19745526 | dried leaves | Spain: La Palma, Monte de Luna, 730 m | *N. Bobon M37_N45* (MJG-030605) |
| *Monanthes wildpretii* Bañares & S.Scholz | M32_N40^b^ | OM953432 | dried leaves | Spain: Tenerife, Chinamada, 711 m | *N. Bobon M32_N40* |
| ***Aeonium* Webb & Berthel.** | | | | | |
| *A. aizoon* (Bolle) T.Mes | TM201.1 | SRR19745515 | flash-frozen leaves | Spain: Tenerife, cliff at the walking path between Barranco de Chacorche and Barranco de Chese, Pinar tinerfeño, E-facing slope, growing together with *Hypericum reflexum* and *Monanthes brachycaulos* | *P. dos Santos, T. Messerschmid & M. Brilhante 2019-21* (MJG-028144) |
| *A. appendiculatum* Bañares | TM246.2 | SRR19745505 | flash-frozen leaves | Spain: La Gomera, near Alajeró, ca. 850 m | *Á. Bañares* 116 (TFC-53764) |
| *A. arboreum* Webb & Berthel. subsp. *arboreum* | TM153.1 | SRR19745502 | dried leaves | Spain: Gran Canaria, at the road GC-704 from San Fernando to Lomo del Peñón | *P. dos Santos & M. Brilhante 37* |
| *A. arboreum* subsp. *holochrysum* (H.Y.Liu) Bañares var. *holochrysum* | TM152.1 | SRR19745504 | dried leaves | Spain: Tenerife, above road TF-38, ca. 1.5 km N of the village Chirche, Lava flows on a WSW-facing slope, growing together with *Pinus canariensis*, *Kleinia neriifolia*, euphorbs, *Aeonium spathulatum* and *A. urbicum* var. *meridionale* | *P. dos Santos, T. Messerschmid & M. Brilhante 2019-12* (MJG-028169) |
| *A. arboreum* subsp. *holochrysum* var. *rubrolineatum* (Svent.) H.Y.Liu | TM211.1 | SRR19745503 | flash-frozen leaves | Spain: La Gomera, near Roque Agando, 1000 m | *Á. Bañares 104* (TFC-53757) |
| *A. aureum* (C.Sm. ex Hornem.) T.Mes | UM2.1 | SRR19745501 | dried flower buds | Spain: Tenerife, Barranco above the road TF-21 from Las Cañadas del Teide to Aguamansa, growing together with *Pterocephalus lasiospermus*, *Silene vulgaris*, *Carlina salicifolia*, *Spartocytisus supranubius*, *Pinus canariensis* and *Aeonium canariense* subsp. *canariense* | *P. dos Santos, T. Messerschmid & M. Brilhante 2019-18* (MJG-028137) |
| *A. balsamiferum* Webb & Berthel. | TM178.1 | SRR19745500 | flash-frozen leaves | Garden origin | *S. Scholz* (MJG-030599) |
| *A. canariense* (L.) Webb & Berthel. subsp. *canariense* | TM169.1 | SRR19745499 | dried leaves | Spain: Tenerife, walking path from Las Bodegas into the laurel forest | *P. dos Santos, T. Messerschmid & M. Brilhante 2019-06* (MJG-028140) |
| *A. canariense* subsp. *christii* (Burchard) Bañares | TM189.1 | SRR19745525 | flash-frozen leaves | Spain: La Palma, Barranco de las Angústias | *P. dos Santos & M. Brilhante 27* |
| *A. canariense* subsp. *latifolium* (Burchard) Bañares | TM133.1 | SRR19745524 | dried flower buds | Spain: La Gomera, walking path between Roque de Agando and La Laja, ca. 900 m W of La Laja | *P. dos Santos, T. Messerschmid & M. Brilhante 2019-22* (MJG-028166) |
| *A. canariense* subsp. *virgineum* (Webb) Bañares | TM165.1 | SRR19745523 | flash-frozen leaves | Spain: Gran Canaria, Barranco de los Tilos, ca. 1.2 km NE of Lomo del Peñón | *P. dos Santos & M. Brilhante s.n.* (MJG-028177) |
| *A. castello-paivae* Bolle | TM137.1 | SRR19745522 | dried flower buds | Spain: La Gomera, above the road GM-1 from Las Rosas to Agulo, ca. 700 m NW of Agulo | *P. dos Santos, T. Messerschmid & M. Brilhante 2019-30* (MJG-028146) |
| *A. ciliatum* Webb & Berthel. | TM135.2 | SRR19745521 | dried leaves | Spain: Tenerife, walking path from Las Bodegas into the laurel forest | *P. dos Santos, T. Messerschmid & M. Brilhante 2019-08* (MJG-028175, MJG-030006) |
| *A. cuneatum* Webb & Berthel. | TM134.1 | SRR19745520 | dried flower buds | Spain: Tenerife, El Bailadero, above the road TF-123 from El Bailadero to Las Bodegas, laurel forest, growing together with *Erica arborea*, *Canarina canariensis*, *Phyllis nobla* and figworts | *P. dos Santos, T. Messerschmid & M. Brilhante 2019-10* (MJG-028154) |
| *A. cuneatum* Webb & Berthel. | FJ11^b^ | OM953427 | dried leaves | Spain: Tenerife, Sierra de Anaga | – |
| *A. davidbramwellii* H.Y.Liu | TM202.1 | SRR19745519 | flash-frozen leaves | Spain: La Palma, Barranco de las Angústias | *P. dos Santos & M. Brilhante 30* (MJG-028176) |
| *A. davidbramwellii* H.Y.Liu | –^b^ | AY082129^c^ | dried leaves | Spain: La Palma | *Mort 1458* (WS) |
| *A. decorum* Webb ex Bolle var. *decorum* | UM3.1 | SRR19745518 | dried flower buds | Spain: La Gomera, ca. 400 m WNW of La Laja | *P. dos Santos, T. Messerschmid & M. Brilhante 2019-24* (MJG-028181) |
| *A. diplocyclum* (Webb ex Bolle) T.Mes | TM195.1 | SRR19745517 | flash-frozen leaves | Spain: La Gomera, walking path between Roque de Agando and La Laja, ca. 900 m W of La Laja | *P. dos Santos, T. Messerschmid & M. Brilhante 2019-23* (MJG-028155) |
| *A. dodrantale* (Willd.) T.Mes | TM196.1 | SRR19745516 | flash-frozen leaves | Spain: Tenerife, slope close to the road TF-436, ca. 600 m WNW of Santiago del Teide | *P. dos Santos, T. Messerschmid & M. Brilhante 2019-17* (MJG-028182) |
| *A. escobarii* Rebmann & Malkm.-Huss. | TM209.1 | SRR19745514 | flash-frozen leaves | Spain: La Palma, near Las Mimbreras, ca. 650 m | *Á. Bañares 115* (TFC-53758) |
| *A. glandulosum* Webb & Berthel. | TM224.2 | SRR19745513 | flash-frozen leaves | Portugal: Madeira, escarpment near Continente supermarket, Ribeira Brava | *M. Benedito s.n.* (MJG-030876) |
| *A. glutinosum* (Aiton) Webb & Berthel. | FJ21 | SRR19745512 | dried leaves | Portugal: Madeira | *R. Lösch s.n.* (MJG-030600) |
| *A. gomerense* (Praeger) Praeger | TM155.1 | SRR19745511 | dried leaves | Spain: La Gomera, at the road CV-14 from Las Poyatas to Benchijigua | *P. dos Santos & M. Brilhante s.n.* |
| *A. goochiae* Webb & Berthel. | TM184.1 | SRR19745510 | flash-frozen leaves | Spain: La Palma, along the new road between the access road to Los Franceses and Barlovento, above the road, 420 m | *U. Eggli & R. Nyffeler 2340* (ZSS-013038) |
| *A. gorgoneum* J.A.Schmidt | TM185.1 | SRR19745509 | flash-frozen leaves | Cape Verde: Santo Antão | *R. Lösch s.n.* (MJG-030602) |
| *A. haworthii* Webb & Berthel. | FJ05 | SRR19745508 | dried leaves | Spain: Tenerife, Tierra de Trigo, 550 m | *K. Esfeld s.n.* (MJG-030601) |
| *A. hierrense* (Murray) J.Pitard & L.Proust | TM210.1 | SRR19745507 | flash-frozen leaf epidermal shavings | Spain: El Hierro, near Tiñor, ca. 900 m | *Á. Bañares 67* (TFC-53760) |
| *A. korneliuslemsii* H.Y.Liu | FJ01 | SRR19745506 | dried leaves | Morocco: Anti-Atlas, Kerdous massif, between Tanout, Igui Ifred and Agni, "Djbel Imzi", on sandstone and siltstone in crevices, moderate amount of humus, probably nutrient rich (and probably containing lime), ca. 600 m | *Erpenbach & Levejohann s.n.* (MJG-030604) |
| *A. lancerottense* (Praeger) Praeger | TM197.1 | SRR19761970 | flash-frozen leaves | Spain: Lanzarote, Famara massif, southwesternmost foothill to the bungalow colony, basalt rocks, ca. 200 m | *E. Royl Ro 384* (MJG-030603) |
| *A. leucoblepharum* Webb ex A.Rich. | TM157.2 | SRR19761969 | dried leaves | Somalia: Puntland, Bari Region, Boosaaso Distr., hills above Galgala | *Lavranos*  *7304* or *9009* (MJG-030608) |
| *A. leucoblepharum* Webb ex A.Rich. | TM238.1 | SRR19761958 | flash-frozen leaves | Yemen: Sumara pass, gorge leading up to Himyasitik fort, 2400 m | *Lavranos & Newton 15996* (ZSS-4877) |
| *A. lindleyi* Webb & Berthel. subsp. *lindleyi* | TM190.1 | SRR19761955 | flash-frozen leaves | Spain: Tenerife, Anaga | *P. dos Santos & M. Brilhante 15* (MJG-028148) |
| *A. lindleyi* subsp. *viscatum* (Bolle) Bañares | –^b^ | AY082154^c^ | dried leaves | Spain: La Gomera | *Mort 1432* (WS) |
| *A. lindleyi* subsp. *viscatum* (Bolle) Bañares | TM198.1 | SRR19761954 | dried leaves | Spain: San Sebastián de La Gomera, Valle San Sebastián, above Barranco Seco, near route from San Sebastián to Tunél de la Cumbre (GM-1) | *W. Rauh 66936* |
| *A. mascaense* Bramwell | TM207.1 | SRR19761953 | flash-frozen leaves | Garden origin | *Á. Bañares 79* (TFC-53761) |
| *A. nobile* (Praeger) Praeger | TM191.1 | SRR19761952 | flash-frozen leaves | Spain: La Palma, Mirador de El Time, above the road LP-1 to Argual | *P. dos Santos & M. Brilhante 28* |
| *A. percarneum* (Murray) J.Pitard & L.Proust | TM192.1 | SRR19761951 | flash-frozen leaves | Spain: Gran Canaria, at the road GC-606 from Carrizal de Tejeda to El Toscón | *P. dos Santos & M. Brilhante 34* (MJG-028171) |
| *A. percarneum* (Murray) J.Pitard & L.Proust | –^b^ | AY082146^c^ | dried leaves | Spain: Gran Canaria | *Mort 1400* (WS) |
| *A.* *pseudurbicum* Bañares | TM160.1 | SRR19761950 | dried flower buds | Spain: Tenerife, plateau above road TF-436, close to the parking lot of the village Masca | *P. dos Santos & T. Messerschmid 2019-15* (MJG-028172) |
| *A. saundersii* Bolle | TM188.1 | SRR19761949 | flash-frozen leaves | Spain: La Gomera | MJG-030606 |
| *A. sedifolium* (Webb ex Bolle) J.Pitard & L.Proust | TM187.1 | SRR19761968 | flash-frozen leaves | Spain: Tenerife, Guía de Isora, S of Morro de los Cerrillos, Parque Nacional del Teide, 2000 m | *W. Rauh KaI/23* (MJG-030607) |
| *A. sedifolium* (Webb ex Bolle) J.Pitard & L.Proust | TM242.1 | SRR19761967 | flash-frozen leaves | Spain: Tenerife, plateau above road TF-436, close to the parking lot of the village Masca | *P. dos Santos & T. Messerschmid 2019-16* (MJG-028162) |
| *A. simsii* (Sweet) Stearn | TM193.1 | SRR19761966 | flash-frozen leaves | Spain: Gran Canaria, Barranco del Palmar, just above the road GC-216 | *P. dos Santos & M. Brilhante s.n.* (MJG-028183) |
| *A. smithii* Webb & Berthel. | TM199.1 | SRR19761965 | flash-frozen leaves | Spain: Tenerife, cliff next to the walking path between Barranco de Chacorche and Barranco de Chese | *P. dos Santos, T. Messerschmid & M. Brilhante 2019-20* (MJG-028174) |
| *A.* *spathulatum* (Hornem.) Praeger | UM1.1 | SRR19761964 | dried flower buds | Spain: Tenerife, above the road TF-21 from Las Cañadas del Teide to Aguamansa, ca. 3.5 km NE from Las Cañadas del Teide | *P. dos Santos, T. Messerschmid & M. Brilhante 2019-19* (MJG-028143) |
| *A. stuessyi* H.Y.Liu | TM203.1 | SRR19761963 | flash-frozen leaves | Ethiopia: Lalibela, 2800-3000 m | *E. Fischer* |
| *A. tabuliforme* Webb & Berthel. | TM212.1 | SRR19761962 | flash-frozen leaf epidermal shavings | Spain: Tenerife, above Los Carrizales, ca. 650 m | *Á. Bañares 128* (TFC-  53762) |
| *A. undulatum* Webb & Berthel. | TM95.1 | SRR19761961 | dried leaves | Spain: Gran Canaria, Tejeda, 900 m | *W. Rauh 66886* (MJG-030610) |
| *A. urbicum* (C.Sm. ex Hornem.) Webb & Berthel. subsp. *urbicum* | TM131.1 | SRR19761960 | dried flower buds | Spain: Tenerife, ca. 1 km S of the village Teno Alto | *P. dos Santos, T. Messerschmid & M. Brilhante 2019-04* (MJG-028157, MJG-028170) |
| *A. urbicum* subsp. *meridionale* Bañares | TM200.1 | SRR19761959 | flash-frozen leaves | Spain: Tenerife, above road TF-38 and Restaurante Boca Tauce, ca. 1.5 km N of the village Chirche | *P. dos Santos, T. Messerschmid & M. Brilhante 2019-13* (MJG-028173) |
| *A. valverdense* (Praeger) Praeger | TM213.1 | SRR19761957 | flash-frozen leaf epidermal shavings | Spain: El Hierro, below Valverde, ca. 450 m | *Á. Bañares 32* (TFC-53763) |
| *A. volkeri* E.Hern. & Bañares | TM194.1 | SRR19761956 | flash-frozen leaves | Spain: Tenerife, Anaga, ca. 1.2 km N of Valle Crispín | *P. dos Santos & M. Brilhante 22* (MJG-028139) |

^a^ Only for accessions with sequences generated by the authors

^b^ Sample only used for the Crassulaceae-wide dating analysis of ITS sequences (see "Molecular dating" in the Materials and Methods)

^c^ Mort et al. (2002)
