# Supplementary material for: Inter- and intra-island speciation and their morphological and ecological correlates in Aeonium (Crassulaceae), a species-rich Macaronesian radiation
Source: Ann Bot. 2023 Feb 23;131(4):697–721. doi: 10.1093/aob/mcad033 (PMC10147336; doi:10.1093/aob/mcad033)
Supplement: mcad033_suppl_Supplementary_Table_S2 [file mcad033_suppl_supplementary_table_s2.docx]

**Table S2:** Counts of sympatric diversification events over the 50 simulations of the Biogeographical Stochastic Mapping analysis (see Supplementary data File S3). Counts are ordered in columns by the geographical areas in which the sympatric diversification events occurred. P: La Palma, G: La Gomera, T: Tenerife, C: Gran Canaria, F: Fuerteventura, L: Lanzarote, Mc: Morocco, EA: East Africa, Md: Madeira, V: Cape Verde.

| Simulation | P | PGT | G | GT | GTC | T | TC | TCMc | TF | TFV | TL | TV | C | CV | L | EA | Md |
| --- | --- | --- | --- | --- | --- | --- | --- | --- | --- | --- | --- | --- | --- | --- | --- | --- | --- |
| 1 | 1 | 1 | 2 | 0 | 0 | 15 | 0 | 0 | 0 | 0 | 0 | 0 | 1 | 0 | 0 | 2 | 1 |
| 2 | 1 | 1 | 5 | 1 | 0 | 13 | 0 | 0 | 0 | 0 | 0 | 0 | 1 | 0 | 0 | 2 | 1 |
| 3 | 1 | 1 | 1 | 5 | 1 | 14 | 0 | 0 | 0 | 0 | 0 | 0 | 1 | 0 | 0 | 2 | 1 |
| 4 | 1 | 1 | 2 | 0 | 0 | 18 | 0 | 0 | 0 | 0 | 0 | 0 | 0 | 0 | 0 | 2 | 1 |
| 5 | 1 | 1 | 2 | 0 | 0 | 18 | 0 | 0 | 0 | 0 | 0 | 0 | 1 | 0 | 0 | 2 | 1 |
| 6 | 1 | 1 | 3 | 0 | 0 | 14 | 0 | 0 | 0 | 0 | 0 | 0 | 3 | 0 | 0 | 2 | 1 |
| 7 | 1 | 1 | 3 | 0 | 0 | 15 | 0 | 0 | 0 | 0 | 0 | 0 | 1 | 0 | 0 | 2 | 1 |
| 8 | 1 | 1 | 3 | 1 | 0 | 15 | 0 | 0 | 0 | 0 | 0 | 0 | 1 | 0 | 1 | 2 | 1 |
| 9 | 1 | 1 | 3 | 0 | 0 | 17 | 0 | 0 | 0 | 0 | 0 | 0 | 1 | 0 | 0 | 2 | 1 |
| 10 | 1 | 1 | 3 | 0 | 0 | 16 | 1 | 0 | 0 | 0 | 0 | 0 | 1 | 0 | 0 | 2 | 1 |
| 11 | 1 | 1 | 2 | 0 | 0 | 18 | 0 | 0 | 0 | 0 | 0 | 0 | 1 | 0 | 0 | 2 | 1 |
| 12 | 1 | 1 | 3 | 0 | 0 | 10 | 2 | 0 | 1 | 0 | 0 | 0 | 2 | 0 | 0 | 2 | 1 |
| 13 | 1 | 1 | 3 | 0 | 0 | 15 | 0 | 0 | 0 | 0 | 0 | 0 | 1 | 0 | 0 | 2 | 1 |
| 14 | 1 | 1 | 2 | 4 | 1 | 10 | 0 | 0 | 0 | 0 | 0 | 0 | 2 | 0 | 0 | 2 | 1 |
| 15 | 1 | 1 | 3 | 0 | 0 | 15 | 1 | 0 | 0 | 0 | 0 | 0 | 0 | 0 | 0 | 1 | 1 |
| 16 | 1 | 1 | 2 | 0 | 0 | 16 | 0 | 0 | 0 | 0 | 1 | 0 | 1 | 0 | 0 | 2 | 1 |
| 17 | 1 | 1 | 2 | 0 | 0 | 18 | 1 | 0 | 0 | 0 | 0 | 0 | 1 | 0 | 0 | 2 | 1 |
| 18 | 1 | 1 | 3 | 0 | 0 | 15 | 1 | 0 | 0 | 0 | 0 | 0 | 0 | 0 | 0 | 2 | 2 |
| 19 | 1 | 1 | 2 | 0 | 0 | 17 | 0 | 0 | 0 | 1 | 0 | 0 | 1 | 0 | 0 | 2 | 1 |
| 20 | 1 | 1 | 3 | 0 | 0 | 15 | 1 | 0 | 0 | 0 | 0 | 0 | 1 | 0 | 0 | 2 | 1 |
| 21 | 1 | 1 | 2 | 0 | 0 | 19 | 1 | 0 | 0 | 0 | 0 | 0 | 1 | 0 | 0 | 2 | 1 |
| 22 | 1 | 1 | 2 | 0 | 0 | 14 | 0 | 0 | 0 | 0 | 0 | 0 | 1 | 0 | 0 | 2 | 1 |
| 23 | 1 | 1 | 3 | 0 | 0 | 17 | 1 | 0 | 0 | 0 | 0 | 0 | 0 | 0 | 0 | 2 | 1 |
| 24 | 1 | 1 | 2 | 0 | 0 | 18 | 0 | 0 | 0 | 0 | 1 | 0 | 1 | 0 | 0 | 1 | 1 |
| 25 | 1 | 1 | 2 | 0 | 1 | 12 | 0 | 0 | 0 | 0 | 0 | 0 | 5 | 0 | 0 | 2 | 1 |
| 26 | 1 | 1 | 4 | 0 | 0 | 13 | 0 | 0 | 0 | 0 | 0 | 0 | 1 | 0 | 0 | 2 | 1 |
| 27 | 1 | 1 | 3 | 0 | 0 | 12 | 0 | 0 | 0 | 0 | 0 | 0 | 1 | 0 | 0 | 2 | 1 |
| 28 | 1 | 1 | 5 | 0 | 0 | 14 | 0 | 0 | 0 | 0 | 0 | 0 | 1 | 0 | 0 | 2 | 1 |
| 29 | 1 | 1 | 3 | 0 | 0 | 17 | 0 | 0 | 0 | 0 | 0 | 0 | 1 | 0 | 0 | 2 | 1 |
| 30 | 1 | 1 | 2 | 0 | 1 | 17 | 0 | 0 | 0 | 0 | 0 | 0 | 1 | 0 | 0 | 2 | 1 |
| 31 | 1 | 1 | 5 | 0 | 0 | 15 | 0 | 0 | 0 | 0 | 0 | 0 | 0 | 0 | 0 | 2 | 1 |
| 32 | 1 | 1 | 5 | 0 | 0 | 10 | 0 | 0 | 0 | 0 | 0 | 0 | 0 | 1 | 0 | 2 | 1 |
| 33 | 1 | 1 | 2 | 0 | 0 | 18 | 0 | 1 | 0 | 0 | 0 | 0 | 1 | 0 | 0 | 2 | 1 |
| 34 | 1 | 1 | 3 | 0 | 0 | 15 | 1 | 0 | 0 | 0 | 0 | 0 | 1 | 0 | 0 | 2 | 1 |
| 35 | 1 | 1 | 4 | 0 | 0 | 10 | 0 | 0 | 1 | 0 | 0 | 0 | 2 | 0 | 0 | 2 | 1 |
| 36 | 1 | 1 | 2 | 0 | 0 | 11 | 3 | 0 | 0 | 0 | 0 | 0 | 1 | 0 | 0 | 2 | 1 |
| 37 | 1 | 1 | 2 | 0 | 0 | 17 | 0 | 0 | 0 | 0 | 0 | 0 | 1 | 0 | 0 | 2 | 1 |
| 38 | 1 | 1 | 4 | 5 | 0 | 12 | 0 | 0 | 0 | 0 | 0 | 0 | 0 | 0 | 0 | 2 | 1 |
| 39 | 1 | 1 | 4 | 0 | 0 | 10 | 4 | 0 | 0 | 0 | 0 | 0 | 1 | 0 | 0 | 2 | 1 |
| 40 | 1 | 1 | 6 | 1 | 0 | 12 | 1 | 0 | 0 | 0 | 0 | 0 | 1 | 0 | 0 | 2 | 1 |
| 41 | 1 | 1 | 2 | 0 | 0 | 18 | 0 | 0 | 0 | 0 | 0 | 0 | 1 | 0 | 0 | 2 | 1 |
| 42 | 1 | 1 | 3 | 0 | 0 | 17 | 0 | 0 | 0 | 0 | 0 | 0 | 1 | 0 | 0 | 2 | 1 |
| 43 | 1 | 1 | 2 | 0 | 0 | 13 | 0 | 0 | 0 | 0 | 0 | 1 | 1 | 0 | 0 | 2 | 3 |
| 44 | 1 | 1 | 0 | 0 | 0 | 16 | 0 | 0 | 0 | 0 | 1 | 0 | 1 | 0 | 0 | 2 | 1 |
| 45 | 1 | 1 | 5 | 0 | 0 | 11 | 0 | 0 | 0 | 0 | 1 | 0 | 1 | 0 | 0 | 2 | 1 |
| 46 | 1 | 1 | 4 | 0 | 0 | 13 | 0 | 0 | 0 | 0 | 0 | 0 | 1 | 0 | 0 | 2 | 1 |
| 47 | 1 | 1 | 2 | 0 | 0 | 18 | 0 | 0 | 0 | 0 | 0 | 0 | 1 | 0 | 0 | 2 | 1 |
| 48 | 1 | 1 | 4 | 0 | 0 | 14 | 1 | 0 | 0 | 0 | 0 | 0 | 1 | 0 | 0 | 2 | 1 |
| 49 | 1 | 1 | 3 | 0 | 0 | 16 | 0 | 0 | 0 | 0 | 0 | 0 | 0 | 0 | 0 | 2 | 1 |
| 50 | 1 | 1 | 2 | 0 | 0 | 18 | 0 | 0 | 0 | 0 | 0 | 0 | 1 | 0 | 0 | 2 | 1 |
| Mean | 1 | 1 | 2.88 | 0.34 | 0.08 | 14.82 | 0.38 | 0.02 | 0.04 | 0.02 | 0.08 | 0.02 | 1.02 | 0.02 | 0.02 | 1.96 | 1.06 |
| Standard deviation | 0 | 0 | 1.17 | 1.14 | 0.27 | 2.64 | 0.81 | 0.14 | 0.20 | 0.14 | 0.27 | 0.14 | 0.80 | 0.14 | 0.14 | 0.20 | 0.31 |
