# Supplementary material for: Inter- and intra-island speciation and their morphological and ecological correlates in Aeonium (Crassulaceae), a species-rich Macaronesian radiation
Source: Ann Bot. 2023 Feb 23;131(4):697–721. doi: 10.1093/aob/mcad033 (PMC10147336; doi:10.1093/aob/mcad033)
Supplement: mcad033_suppl_Supplementary_Table_S4 [file mcad033_suppl_supplementary_table_s4.docx]

**Table S4.** Raw Euclidean Distances (REDs; 0 corresponding to full identity and 1 corresponding to full disparity) in the individual morphological characters (i.e., flower merism and characters k–t in Fig. 3) for those nodes that were recovered in all phylogenetic analyses and received high support in at least one of these analyses. For each node, the biogeographical correlate of diversification (i.e., inter- or intra-island diversification) as reconstructed in the BSM analysis is given, along with the islands on which diversification took place and the percentage of BSMs supporting this scenario. For the sister lineages corresponding to each node, see Table 6.

| Node number^a^ | Biogeographical correlate of diversification | Flower merism | Growth form | Branch indumentum | Leaf scars | Leaf cilia | Leaf pubescence | Leaf tannic stripes | Inflorescence pubescence | Petal colour | Carpel appendages | Nectaries |
| --- | --- | --- | --- | --- | --- | --- | --- | --- | --- | --- | --- | --- |
| 9 | 94 % inter-island (92 % Tenerife - La Gomera) | 0.94 | 0.00 | 0.00 | 0.00 | 0.00 | 0.50 | 0.00 | 0.00 | 0.00 | 0.00 | 0.00 |
| 10 | 100 % intra-island (100 % Tenerife) | 0.94 | 0.00 | 0.00 | 0.00 | 0.00 | 0.50 | 0.00 | 0.00 | 0.00 | 0.00 | 0.00 |
| 11 | 90 % intra-island (90 % Tenerife) | 0.83 | 0.00 | 0.00 | 0.00 | 0.00 | 1.00 | 0.00 | 0.00 | 0.00 | 0.00 | 0.00 |
| 13 | 100 % inter-island (96 % Tenerife - La Palma) | 0.67 | 0.00 | 0.00 | 0.00 | 0.00 | 0.00 | 0.00 | 0.00 | 1.00 | 0.00 | 0.00 |
| 15 | 82 % intra-island (82 % Gran Canaria) | 0.50 | 1.00 | 0.00 | 0.00 | 0.00 | 0.50 | 1.00 | 0.50 | 0.00 | 0.00 | 0.00 |
| 16 | 100 % inter-island (ambiguous) | 0.50 | 0.00 | 0.00 | 0.00 | 0.00 | 0.50 | 0.00 | 0.50 | 0.00 | 0.00 | 0.00 |
| 17 | 100 % inter-island (ambiguous) | 0.40 | 0.00 | 0.00 | 0.00 | 0.00 | 0.50 | 0.00 | 0.50 | 0.00 | 0.00 | 0.00 |
| 20 | 100 % inter-island (100 % Gran Canaria - La Gomera) | 0.00 | 0.00 | 0.00 | 0.00 | 0.00 | 0.00 | 0.00 | 1.00 | 0.00 | 0.00 | 0.00 |
| 22 | 100 % inter-island (98 % Tenerife - Madeira) | 0.50 | 0.50 | 0.00 | 0.00 | 0.50 | 0.00 | 0.00 | 0.00 | 0.00 | 0.00 | 0.00 |
| 23 | 100 % intra-island (100 % Madeira) | 0.50 | 1.00 | 0.00 | 0.00 | 0.00 | 1.00 | 0.00 | 0.00 | 1.00 | 0.00 | 0.00 |
| 24 | 98 % intra-island (98 % Tenerife) | 0.67 | 0.00 | 0.00 | 0.00 | 1.00 | 1.00 | 0.00 | 0.00 | 1.00 | 0.00 | 0.00 |
| 25 | 84 % intra-island (84 % Tenerife) | 0.00 | 0.00 | 0.00 | 0.00 | 0.00 | 0.00 | 0.00 | 0.00 | 0.00 | 0.00 | 0.00 |
| 26 | 100 % inter-island (100 % Tenerife - La Gomera) | 0.50 | 0.00 | 0.00 | 0.00 | 0.00 | 0.00 | 0.00 | 0.00 | 0.00 | 0.00 | 0.00 |
| 27 | 98 % inter-island (92 % Tenerife - Gran Canaria) | 0.33 | 0.00 | 0.00 | 0.00 | 0.00 | 0.00 | 0.00 | 0.00 | 0.00 | 0.00 | 0.00 |
| 28 | 100 % inter-island (ambiguous) | 0.00 | 0.00 | 0.00 | 0.00 | 0.00 | 0.00 | 0.00 | 0.00 | 0.00 | 0.00 | 0.00 |
| 33 | 94 % intra-island (94 % La Gomera) | 0.50 | 0.00 | 1.00 | 1.00 | 0.00 | 0.50 | 0.00 | 0.00 | 0.00 | 0.00 | 0.00 |
| 45 | 100 % intra-island (100 % Tenerife) | 0.40 | 0.50 | 0.00 | 0.50 | 0.00 | 0.50 | 0.00 | 0.50 | 0.00 | 0.50 | 0.00 |
| 46^b^ | 100 % intra-island (100 % Tenerife) | 0.40 | 0.50 | 0.00 | 0.50 | 0.00 | 0.50 | 0.00 | 0.50 | 0.00 | 1.00 | 0.00 |
| 47^b^ | 100 % intra-island (100 % Tenerife) | 0.60 | 0.50 | 0.00 | 0.50 | 0.00 | 0.50 | 0.00 | 0.50 | 0.00 | 0.00 | 0.00 |
| 48^b^ | 100 % intra-island (100 % Tenerife) | 0.60 | 1.00 | 0.00 | 1.00 | 0.00 | 0.50 | 0.00 | 1.00 | 0.00 | 0.00 | 0.00 |
| 49^b^ | 100 % intra-island (100 % Tenerife) | 0.25 | 1.00 | 0.00 | 0.00 | 0.00 | 1.00 | 0.00 | 0.00 | 0.00 | 0.00 | 0.00 |
| Mean of Tenerifean *Leuconium* subclade^c^ | 100 % intra-island (100 % Tenerife) | 0.40 | 0.63 | 0.00 | 0.38 | 0.00 | 0.63 | 0.00 | 0.54 | 0.00 | 0.33 | 0.00 |

^a^ Node numbers as specified in Fig. 4

^b^ These nodes in the Tenerifean subclade of sect. *Leuconium* were retrieved in the dating analysis only.

^c^ These values are REDs averaged across the nodes of the Tenerifean subclade of sect. *Leuconium* above node 45 (i.e., the inconsistently resolved nodes 46–49) as recovered in all our phylogenetic analyses. See paragraph 2.8 (above) for more detailed information
